# Characterizing Digital Communication Device Use Among Young People From 4 European Countries: Cross-Sectional Survey Study

**DOI:** 10.2196/76767

**Published:** 2025-12-23

**Authors:** Matthew L Stamets, Gemma Castaño-Vinyals, Patricia de Llobet Viladoms, Adriana Fernandes Veludo, Arno Thielens, Leanne Martin, Robin Wydaeghe, Sam Aerts, Marta Parazzini, Gabriella Tognola, Joe Wiart, Kinga Polanska, Maja Popovic, Maria-José López, Milena Maule, Wout Joseph, James Grellier, Martin Röösli, Mònica Guxens

**Affiliations:** 1 ISGlobal, Barcelona Biomedical Research Park (PRBB), Doctor Aiguader, 88, Barcelona, 08003, Spain, 34 93 214 7300; 2 Universitat Pompeu Fabra, Barcelona, Spain; 3 Spanish Consortium for Research on Epidemiology and Public Health (CIBERESP), Instituto de Salud Carlos III, Madrid, Spain; 4 Swiss Tropical and Public Health Institute, Allschwil, Switzerland; 5 University of Basel, Basel, Switzerland; 6 Advanced Science Research Center, The Graduate Center of the City University of New York (CUNY), New York, NY, United States; 7 European Centre for Environment and Human Health, University of Exeter Medical School, University of Exeter, Penryn, United Kingdom; 8 Department of Information Technology, Ghent University/IMEC, Ghent, Belgium; 9 Smart Sensor Systems Research Group, The Hague University of Applied Sciences, Delft, The Netherlands; 10 CNR – Istituto di Elettronica e di Ingegneria dell’Informazione e delle Telecomunicazioni, Milan, Italy; 11 LTCI, Telecom Paris, Institut Polytechnique de Paris, Palaiseau, France; 12 Department of Environmental and Occupational Health Hazards, Nofer Institute of Occupational Medicine, Lodz, Poland; 13 Cancer Epidemiology Unit, Department of Medical Sciences, University of Turin and CPO-Piemonte, Turin, Italy; 14 Agencia de Salud Pública de Barcelona, Barcelona, Spain; 15 Institut de Recerca Sant Pau, Barcelona, Spain; 16 Department of Child and Adolescent Psychiatry/Psychology, University Medical Centre, Erasmus MC, Rotterdam, The Netherlands; 17 ICREA, Barcelona, Spain

**Keywords:** smartphone, adolescent, mobile apps, wearable electronic devices, population characteristics, prevalence, wireless technology, Europe

## Abstract

**Background:**

Digital communication device use is changing rapidly among young people, and current research on this topic is limited or outdated.

**Objective:**

We aimed to describe the use of digital communication devices by young people from 4 European countries and investigate their socioeconomic and demographic characteristics.

**Methods:**

In 2023, we administered an online survey to a convenience sample of 4000 young people aged 16 to 25 years in Italy, Poland, Spain, and Switzerland. Participants reported on their regular use of smartphones, tablets, laptops, cordless phones, and smartwatches or activity trackers. Participants answered which activities they regularly engaged in on their devices, the time spent on these devices and activities, and in what position the device was used with respect to their body over the previous 3 months. We also collected information on participant socioeconomic and demographic characteristics, including age, gender, country of birth, employment status, parental educational level, and urbanicity of the place of residence.

**Results:**

Reported prevalence of device use was 90.9% (3635/4000) for smartphones, 33.2% (1329/4000) for tablets, 68.7% (2748/4000) for laptops, 11.6% (462/4000) for cordless phones, and 23.3% (931/4000) for smartwatches or activity trackers. Older age groups and women reported higher use across most devices. The activities reported with the highest engagement for smartphones were voice calls (2553/3635, 70.2%); social media (2693/3635, 74.1%); and texting, emailing, and internet use (2530/3635, 69.6%). For tablets and laptops, they were video streaming (849/1329, 63.9% and 1527/2748, 55.6%, respectively); texting, emailing, and internet use (673/1329, 50.6% and 1218/2748, 44.3%, respectively); and social media (659/1329, 49.6% and 1521/2748, 55.3%, respectively). On average, participants used their smartphones 60.9 (SD 83.1) minutes per day for texting, emailing, and internet use; 85.2 (SD 92.7) minutes per day for social media; 46.9 (SD 70.5) minutes per day for video streaming; and 53.7 (SD 80.3) minutes per day for music streaming. Differences across activities and devices were found among socioeconomic and demographic characteristics. For example, the oldest age groups reported lower duration of smartphone use for voice calls, social media, video streaming, and music streaming compared to the youngest age group but reported higher duration of smartphone use for video calls and texting, emailing, and internet use. Moreover, women reported higher duration of use for most activities on smartphones compared to men, except for online gaming, for which men reported higher duration of use.

**Conclusions:**

Our findings provide novel information on digital communication device use by young people. We identified differences between socioeconomic and demographic characteristics that warrant further investigation. These results can be used as a point of reference for digital communication devices in public health research, including health communication strategies and epidemiological research.

## Introduction

The everyday use of digital communication devices has become a societal norm. Devices used to access the internet are at a historic peak, with European internet users opting to connect 86% of the time using mobile phones or smartphones, 63% of the time using laptops or tablets, and 33% of the time using other mobile devices such as game consoles or smartwatches in 2023 [1]. Young people in particular have grown up with these technologies, in contrast to older generations. In addition, owing to patterns of social behavior changing during the COVID-19 pandemic, especially with regard to academic settings, reliance on technology has been most notably heightened among these younger populations [2,3].

As young people are increasingly using digital communication devices, concerns about their potential impact on health have intensified. In recent years, researchers in many disciplines have attempted to better understand the plausibility and magnitude of potential risks to health. These include studies in fields ranging from psychology and the social sciences investigating how people use social media and the impacts of excessive device use on mental health [4-7] to epidemiological studies on the potential health impacts of radiofrequency electromagnetic fields (RF-EMFs) [8-12].

For researchers to investigate potential health risks associated with digital communication devices, comprehensive data on all aspects of use are required. However, studies that explore digital communication device use do not usually aim to conduct health-related research and, thus, lack critical information about use patterns in socioeconomic and demographic groups or are focused only on late childhood and adolescence. This means that very limited information on device use in different demographics, especially young adults, is available to researchers. However, the data that are currently available have been collected years or a decade ago and may relate to devices that are no longer relevant to young people, hence informing little about estimates of current use. Furthermore, collected information on certain characterizations of digital communication devices is limited, such as the use of wearable technology or the position of the device when in use [13-16]. For example, it is known that the type of device used, the activity being performed with the device, and the position of the device relative to the body during use all determine individual exposure to RF-EMFs [17]. How young people use devices, applications, and communication technologies today varies significantly from use habits a decade ago, and it is crucial to understand how this evolves over time. Investigating use habits extensively and disseminating the resulting insights is essential for advancing research on the relationships between health and digital communication device use. We anticipated that patterns across various characterizations of digital communication device use would differ across socioeconomic and demographic groups rather than exhibiting consistent trends. Therefore, the aim of this study was to characterize the use of digital communication devices in individuals aged 16 to 25 years in 4 European countries and investigate its variation across different socioeconomic and demographic groups.

## Methods

### Study Design and Population

This cross-sectional study was conducted as part of the GOLIAT (“5G exposure, causal effects, and risk perception through citizen engagement”) project. An online panel survey was administered in July 2023 in 4 European countries: Italy, Poland, Spain, and Switzerland. A total of 4000 participants (n=1000, 25% per country) aged 16 to 25 years were recruited through convenience sampling via the survey platform Cint (Cint Group) [18]. Participants were invited to the survey via email by Cint. In the invitation, the survey was described to the participants, along with the GOLIAT project and the research project’s intent to investigate the use of digital communication devices. They were informed that the survey would take roughly 15 to 20 minutes and that monetary compensation would be given upon completion of the survey. The survey was pretested by the Barcelona Institute for Global Health and Cint to ensure usability and technical functionality. An initial screening was performed with 223 participants to resolve any unexpected issues before administering the final version to all participants. Surveys were accessible in the official languages of the countries surveyed or in English. The survey can be found in Multimedia Appendix 1.

To ensure representation of age, gender, and urbanicity within the sample, the survey was administered using noninterlocking quotas. Additional details of the quotas can be found in Multimedia Appendix 2. A total of 85,598 participants were invited, with 6180 (7.2%) initially agreeing to take part. After screening out participants based on noncompletion of the survey, participants having unsubscribed from the platform, or quotas being filled, responses from 4000 participants were obtained. More detailed reporting of the survey can be found in the Checklist for Reporting Results of Internet E-Surveys [19].

### Socioeconomic and Demographic Characteristics

Information was collected on socioeconomic characteristics, namely, employment status, highest parental educational level, and urbanicity of the place of residence. Employment status was categorized as student, working full time, working part time, working and studying, and other (which included being unemployed; being a housewife or househusband; being a carer for children, older adults, or a person with illness or disability; being on long-term sick leave or disability; and other). The participants’ highest maternal or paternal educational level was categorized as university or higher, vocational education, secondary school, and primary school or lower. Urbanicity was self-reported and categorized by the number of inhabitants at the place of residence (city [>100,000 residents], town or suburb [10,000-100,000 residents], and rural [<10,000 residents]).

Participant demographic characteristics were age (16-18 years, 19-22 years, and 23-25 years), gender (man, woman, and nonbinary), and country of birth. Country of birth was categorized as the country in which the survey was carried out versus other.

### Digital Communication Devices

Device use was assessed by asking the participants which of the following devices they used on a regular basis (ie, at least once per week in the previous 3 months): smartphone, tablet, laptop, cordless phone, and smartwatch or activity tracker. Participants who reported regular use of a specific device were then asked follow-up questions about the specific activities they engaged in using these devices on a regular basis. These activities were predefined and specific to each device. For smartphones, activities included voice calls; internet video calls; voice messages; sending videos; texting, sending pictures or emails, or internet browsing (referred to as “texting, emailing, and internet use”); using social media, scrolling through social media, chatting, and uploading pictures or videos (referred to as “social media”); online video streaming; online music streaming or online listening to podcasts (referred to as “music streaming”); online gaming; and use as a hot spot. For tablets and laptops, activities included internet video calls; texting, emailing, and internet use; social media; online video streaming; music streaming; and online gaming.

To assess the duration of time spent engaged in each activity, participants were asked how long they performed the activity separately on weekdays and the weekend. The specific questions asked per device and activity are shown in Table S1 in Multimedia Appendix 2. The response options regarding the duration of use for most activities were 10 minutes or less, 11 to 30 minutes, 31 to 60 minutes, 1 to 2 hours, 2 to 4 hours, and ≥4 hours per day, whereas voice calls and video calls had response options of 5 minutes or less, 6 to 15 minutes, 16 to 30 minutes, 31 to 60 minutes, 1 to 2 hours, and ≥2 hours per day. To assess weekly use for each participant, weekday and weekend responses were combined using median values of the category, multiplied by the number of days in which they engaged in the activity (5 for weekdays and 2 for weekends), and categorized using the previous cutoffs in the scale for time. For example, if participants answered that they engaged in an activity for 31 to 60 minutes on weekdays and 1 to 2 hours on the weekend, we assumed a median value of 45 minutes per day on weekdays (45 × 5 = 225 minutes in total) and 90 minutes per day on the weekend (90 × 2 = 180 minutes in total). After summing the weekday and weekend values together, we divided by the number of days per week ([225 + 180]/7) to obtain an average amount of minutes per day (57.9 minutes), categorizing the value as 31 to 60 minutes per day.

The position of the device during use was assessed for activities that could be performed by participants in multiple ways. These activities included voice calls (smartphone), internet video calls (smartphone and tablet), online video streaming (smartphone and tablet), and music streaming (smartphone and tablet); these are shown in Table S1 in Multimedia Appendix 2. Positions were predefined as “holding phone against my ear” (voice calls only), “holding phone/tablet in front of my eyes,” or “elsewhere.” Item response options to these positions were “never or rarely,” “less than half of the time,” “about half of the time,” “more than half of the time,” and “always or almost always.” In cases in which the answers were not complete or succinct, they were adjusted proportionally. For example, if a participant responded that they used a smartphone for online video streaming in 2 positions (ie, holding the phone or tablet in front of their eyes and elsewhere but reported both as “never or rarely”), answers were adjusted to “about half of the time” for each position.

Participants were also asked open-ended questions about their use of other devices, such as virtual reality glasses, gaming consoles, and augmented reality devices. The duration of time spent using these other devices was also collected, and item response options were 5 minutes or less per week, 6 to 15 minutes per week, 16 to 30 minutes per week, 31 to 60 minutes per week, and more than 60 minutes per week.

### Statistical Analysis

Differences in frequencies of device use and activities for each socioeconomic and demographic factor were assessed using the Pearson chi-square test. As very few participants responded that their gender was neither man nor woman (41/4000, 1%), they were excluded from the analyses only in comparisons by gender. *P* values of <.05 were considered to be statistically significant. Statistical analysis was performed using Stata (version 16.0; StataCorp) and RStudio (version 4.3.3; Posit PBC).

### Ethical Considerations

This study was approved by the Research Ethics Committee of the Mar Health Park (*Parc de Salut Mar*; 2023/10829), Spain. Informed consent was obtained from the participants before enrollment in the study. Anonymized responses were collected, with no access to any personal data following General Data Protection Regulation compliance. Participants were informed that researchers would only have access to their anonymized survey responses. This study was completely voluntary, and consent could be withdrawn through Cint at any point of the survey for any reason without explanation. Upon completion of the survey, participants received monetary compensation.

## Results

### Study Characteristics

The socioeconomic and demographic characteristics of the study participants are presented in Table 1. Most of those who answered were either students (1821/4000, 45.5%) or working full time (1262/4000, 31.5%). Most participants had at least one parent who had either a university education or higher (1490/4000, 37.3%) or vocational education (1418/4000, 35.5%).

**Table 1.** Socioeconomic and demographic characteristics and device use frequency in young adults, stratified by country.

			Overall (n=4000), n (%)			Spain (n=1000), n (%)		Italy (n=1000), n (%)		Poland (n=1000), n (%)		Switzerland (n=1000), n (%)		*P* value	
Age group (y)														<.001	
	16‐18		1090 (27.2)			293 (29.3)		287 (28.7)		298 (29.8)		212 (21.2)			
	19‐22		1647 (41.2)			402 (40.2)		406 (40.6)		401 (40.1)		438 (43.8)			
	23‐25		1263 (31.6)			305 (30.5)		307 (30.7)		301 (30.1)		350 (35)			
Gender														.88	
	Female		1991 (49.8)			500 (50)		500 (50)		500 (50)		491 (49.1)			
	Male		1968 (49.2)			490 (49)		486 (48.6)		492 (49.2)		500 (50)			
	Other		41 (1)			10 (1)		14 (1.4)		8 (0.8)		9 (0.9)			
Employment status														<.001	
	Student		1821 (45.5)			538 (53.8)		587 (58.7)		364 (36.4)		332 (33.2)			
	Working full time		1262 (31.5)			275 (27.5)		211 (21.1)		312 (31.2)		464 (46.4)			
	Working part time		195 (4.9)			55 (5.5)		52 (5.2)		49 (4.9)		39 (3.9)			
	Working and studying		224 (5.6)			44 (4.4)		40 (4)		114 (11.4)		26 (2.6)			
	Other		498 (12.5)			88 (8.8)		110 (11)		161 (16.1)		139 (13.9)			
Country of birth														<.001	
	Country of the study (versus other)		3517 (87.9)			875 (87.5)		923 (92.3)		955 (95.5)		764 (76.4)			
Highest parental education														<.001	
	University or higher		1490 (37.3)			387 (38.7)		300 (30)		451 (45.1)		352 (35.2)			
	Vocational education		1418 (35.5)			214 (21.4)		387 (38.7)		507 (50.7)		310 (31)			
	Secondary school		708 (17.7)			276 (27.6)		167 (16.7)		8 (0.8)		257 (25.7)			
	Primary or lower		384 (9.6)			123 (12.3)		146 (14.6)		34 (3.4)		81 (8.1)			
Urbanicity														.99	
	City		1581 (39.5)			384 (38.4)		400 (40)		400 (40)		397 (39.7)			
	Towns or suburbs		1618 (40.5)			416 (41.6)		400 (40)		400 (40)		402 (40.2)			
	Rural areas		801 (20)			200 (20)		200 (20)		200 (20)		201 (20.1)			
Device use															
	Smartphone		3635 (90.9)			936 (93.6)		937 (93.7)		960 (96)		802 (80.2)		<.001	
	Tablet		1329 (33.2)			329 (32.9)		380 (38)		181 (18.1)		439 (43.9)		<.001	
	Laptop		2748 (68.7)			666 (66.6)		664 (66.4)		702 (70.2)		716 (71.6)		.02	
	Cordless phone		462 (11.6)			129 (12.9)		102 (10.2)		78 (7.8)		153 (15.3)		<.001	
	Smart watches/activity trackers		931 (23.3)			247 (24.7)		220 (22)		261 (26.1)		203 (20.3)		.009	

### Device Use

Overall prevalence of device use reported by participants was 90.9% (3635/4000) for smartphones, 33.2% (1329/4000) for tablets, 68.7% (2748/4000) for laptops, 11.6% (462/4000) for cordless phones, and 23.3% (931/4000) for smartwatches or activity trackers (Table 1).

Differences in the prevalence of use of all devices were observed for most socioeconomic and demographic factors (Table 2). Use of all devices was lower in the age group of 16 to 18 years compared to older age groups except for smartphones, for which use levels were comparable. Women reported higher use of all devices except for cordless phones, for which no differences were observed. Differences by employment status were found for use of all devices, but inconsistent patterns were observed. Participants whose parents had a university- or higher-level education reported the highest use of tablets, laptops, and smartwatches or activity trackers. Those born in the country where the survey was administered reported higher smartphone, laptop, and smartwatch or activity tracker use than those who were not. Smartphone use in rural areas was higher, whereas use of tablets, laptops, and cordless phones was lower.

**Table 2.** Frequencies of device use by socioeconomic and demographic factors in young adults (N=4000).

		Smartphone		Tablet		Laptop		Cordless phone		Smart watches/activity trackers	
		Values, n (%)	*P* value	Values, n (%)	*P* value	Values, n (%)	*P* value	Values, n (%)	*P* value	Values, n (%)	*P* value
Age group			.02		<.001		<.001		<.001		<.001
	16‐18 y	1000 (91.7)		259 (23.8)		660 (60.6)		86 (7.9)		186 (17.1)	
	19‐22 y	1472 (89.4)		609 (37)		1160 (70.4)		206 (12.5)		385 (23.4)	
	23‐25 y	1163 (92.1)		461 (36.5)		928 (73.5)		170 (13.5)		360 (28.5)	
Gender (n=3959)			<.001		.01		<.001		.15		.03
	Female	1855 (93.2)		703 (35.3)		1434 (72)		213 (10.7)		496 (24.9)	
	Male	1746 (88.7)		619 (31.5)		1291 (65.6)		240 (12.2)		430 (21.8)	
Employment status			<.001		<.001		<.001		<.001		<.001
	Student	1697 (93.2)		574 (31.5)		1252 (68.8)		173 (9.5)		375 (20.6)	
	Working full time	1130 (89.5)		501 (39.7)		913 (72.3)		198 (15.7)		372 (29.5)	
	Working part time	188 (96.4)		68 (34.9)		142 (72.8)		14 (7.2)		45 (23.1)	
	Working and studying	212 (94.6)		65 (29)		184 (82.1)		27 (12.1)		69 (30.8)	
	Other	408 (81.9)		121 (24.3)		257 (51.6)		50 (10)		70 (14.1)	
Highest parental education			.09		<.001		<.001		.14		<.001
	University or higher	1355 (90.9)		560 (37.6)		1120 (75.2)		182 (12.2)		385 (25.8)	
	Vocational education	1306 (92.1)		450 (31.7)		958 (67.6)		174 (12.3)		345 (24.3)	
	Secondary school	630 (89)		211 (29.8)		463 (65.4)		72 (10.2)		135 (19.1)	
	Primary or lower	344 (89.6)		108 (28.1)		207 (53.9)		34 (8.9)		66 (17.2)	
Country of birth			<.001		.61		<.001		.34		.04
	Country of the study	3253 (92.5)		1174 (33.4)		2465 (70.1)		413 (11.7)		837 (23.8)	
	Other	382 (79.1)		155 (32.1)		283 (58.6)		49 (10.1)		94 (19.5)	
Urbanicity			.008		.02		.048		.001		.11
	City	1438 (91)		555 (35.1)		1112 (70.3)		202 (12.8)		394 (24.9)	
	Towns or suburbs	1448 (89.5)		540 (33.4)		1111 (68.7)		197 (12.2)		366 (22.6)	
	Rural areas	749 (93.5)		234 (29.2)		525 (65.5)		63 (7.9)		171 (21.3)	

### Activity Engagement on Each Device

Frequencies of activity engagement by device can be found in Table 3. The activities most frequently engaged in on smartphones were social media (2693/3635, 74.1%); voice calls (2553/3635, 70.2%); and texting, emailing, and internet use (2530/3635, 69.6%). For tablets and laptops, these were video streaming (849/1329, 63.9% and 1527/2748, 55.6%, respectively); texting, emailing, and internet use (673/1329, 50.6% and 1218/2748, 44.3%, respectively); and social media (659/1329, 49.6% and 1521/2748, 55.3%, respectively).

**Table 3.** Frequencies of total activity engagement by device in young adults. Percentages are calculated only for respondents who indicated that they had used the particular device.

Activity	Smartphone (n=3635), n (%)	Tablet (n=1329), n (%)	Laptop (n=2748), n (%)
Voice calls	2553 (70.2)	N/A^a^	N/A
Voice messages	2182 (60)	N/A	N/A
Sending videos	1554 (42.8)	N/A	N/A
Video calls	1679 (46.2)	360 (27.1)	2748 (25.3)
Texting, emailing, and internet use	2530 (69.6)	673 (50.6)	1218 (44.3)
Social media use	2693 (74.1)	659 (49.6)	1521 (55.3)
Video streaming	2017 (55.5)	849 (63.9)	1527 (55.6)
Music streaming	2074 (57.1)	390 (29.3)	876 (31.9)
Online gaming	1492 (41)	297 (22.3)	641 (23.3)
Hot spot	707 (19.4)	N/A	N/A

a N/A: not applicable.

### Duration of Use by Device and Activity

**Figure 1.**
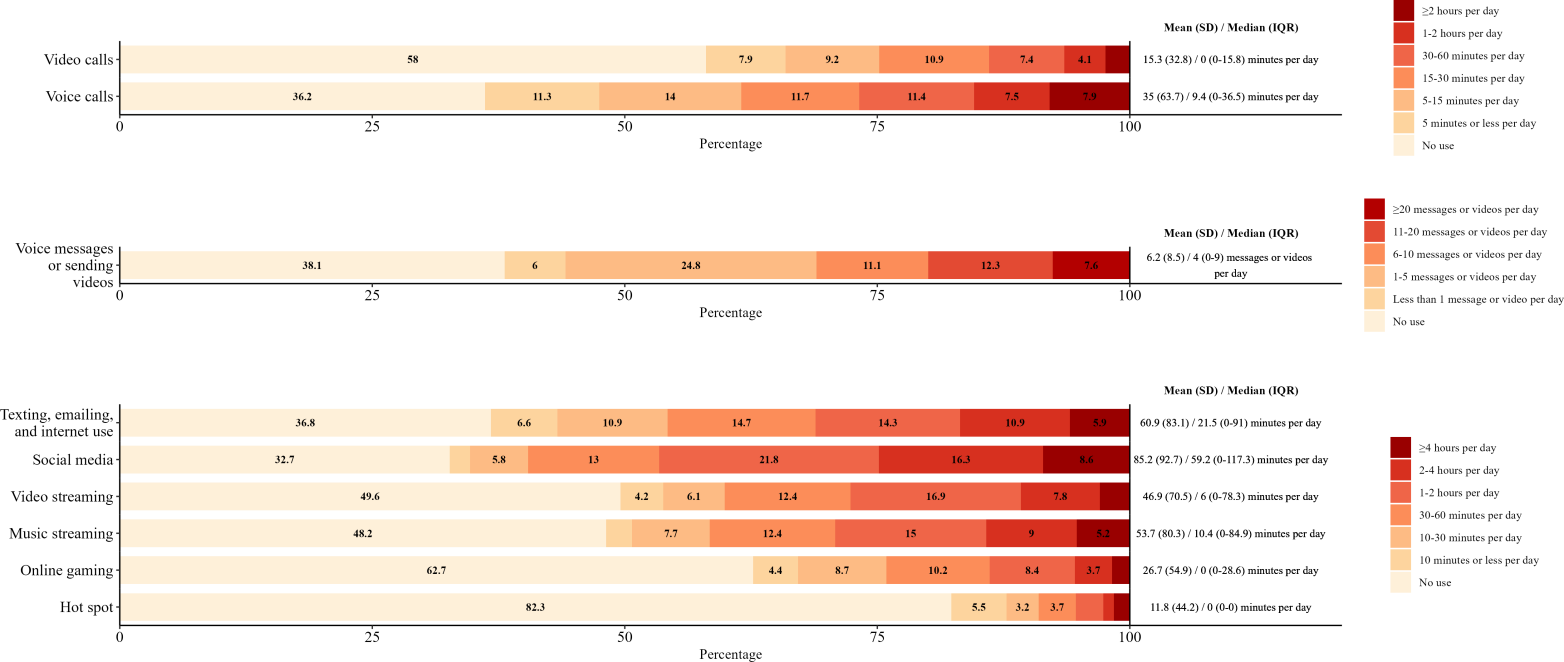
Frequencies of smartphone activity duration per day by activity in young adults. Frequencies of less than 3% are not reported.

**Figure 2.**
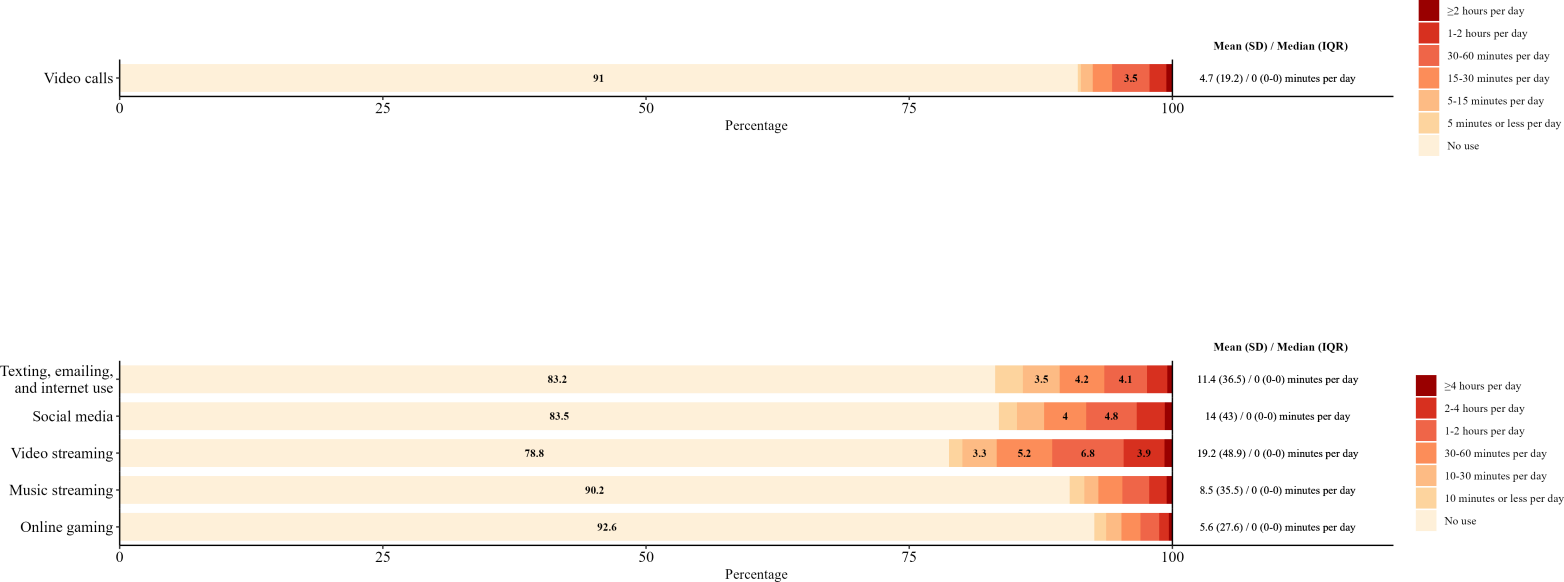
Frequencies of tablet activity duration per day by activity in young adults. Frequencies of less than 3% are not reported.

**Figure 3.**
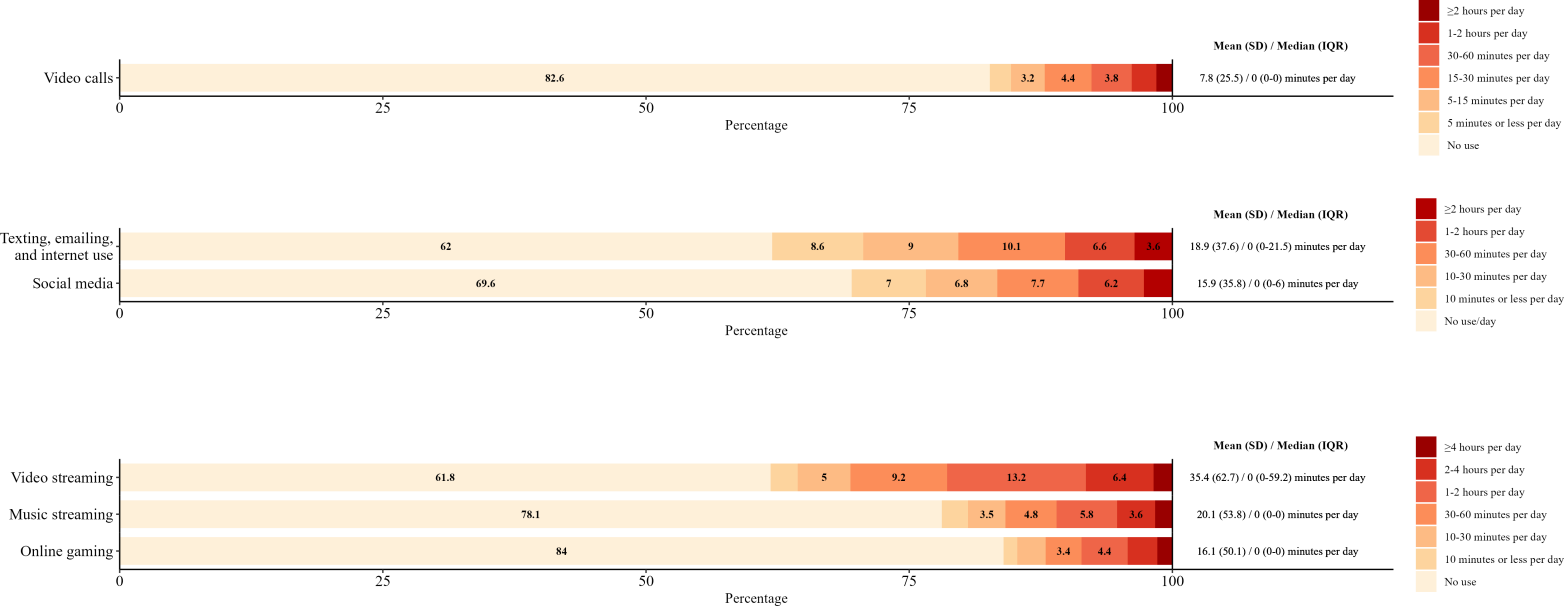
Frequencies of laptop activity duration per day by activity in young adults. Frequencies of less than 3% are not reported.

**Figure 4.**
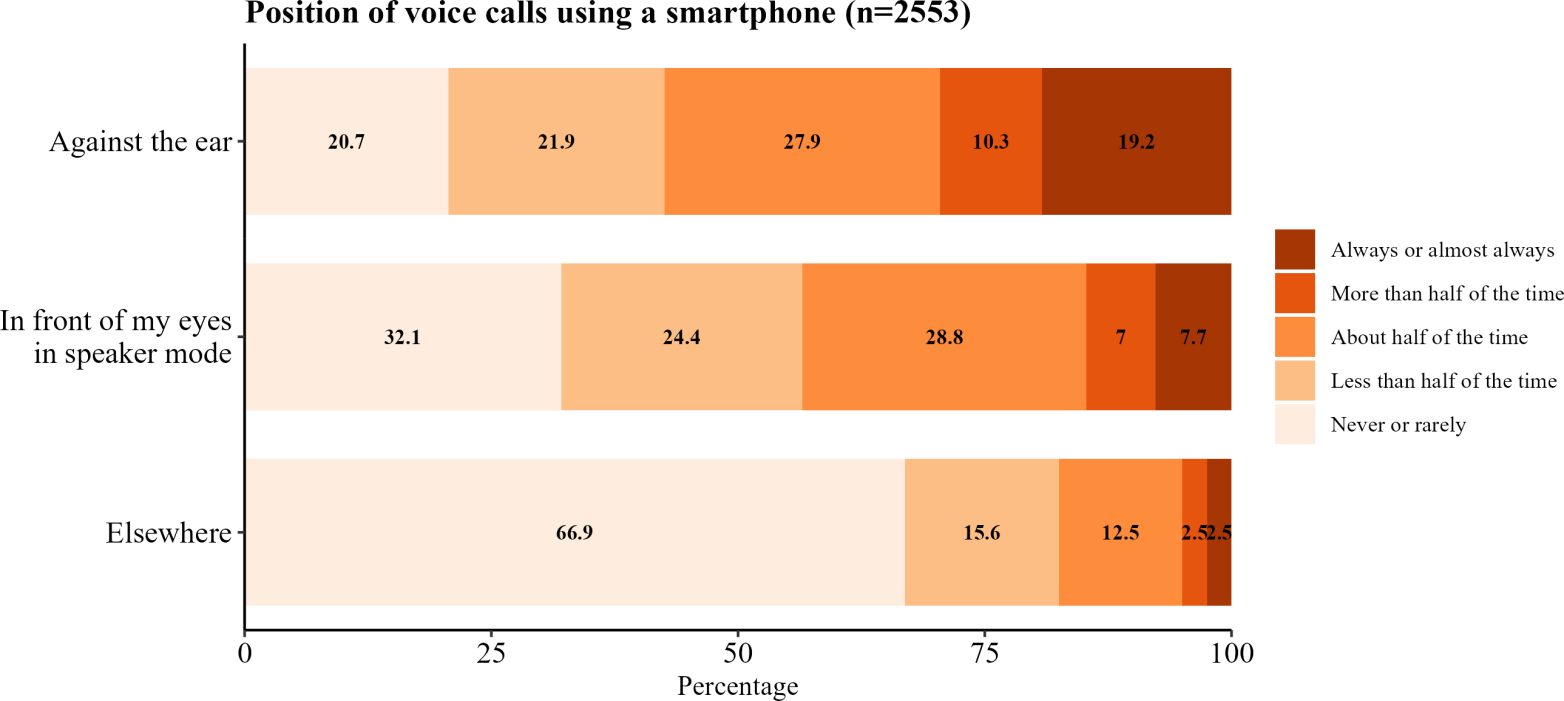
Frequencies of device position for voice calls using a smartphone in young adults.

Duration of use by device and activity can be found in Figures 2-4. For smartphones, texting, emailing, and internet use (mean 60.9, SD 83.1 minutes per day); social media (mean 85.2, SD 92.7 minutes per day); video streaming (mean 46.9, SD 70.5 minutes per day); and music streaming (mean 51.6, SD 80.3 minutes per day) were the activities reported with the highest duration of use (Figure 1). For tablets, video streaming (mean 19.2, SD 48.9 minutes per day); texting, emailing, and internet use (mean 11.4, SD 36.5 minutes per day); and social media (mean 14, SD 43 minutes per day) were reported with the highest duration of use (Figure 2). For laptops, video streaming (mean 35.4, SD 62.7 minutes per day); music streaming (mean 20.1, SD 53.8 minutes per day); and texting, emailing, and internet use (mean 18.9, SD 37.6 minutes per day) were reported with the highest duration of use (Figure 3).

We observed differences in the duration of use for the different activities on each device across socioeconomic and demographic characteristics (Figures S1-S21 in Multimedia Appendix 2). However, inconsistent patterns were observed. For example, the 2 oldest age groups reported lower duration of use of smartphones for voice calls, social media, video streaming, and music streaming compared to the youngest age group but reported higher duration of use of smartphones related to video calls and texting, emailing, and internet use (Figure S1 in Multimedia Appendix 2). Regarding tablets and laptops, the oldest age group reported higher duration of use for video calls; texting, emailing, and internet use; social media; video streaming; and music streaming but lower duration of use for online gaming compared to the youngest age group (Figures S2 and S3 in Multimedia Appendix 2). Furthermore, women reported higher duration of use for almost all activities on smartphones compared to men, except for online gaming, for which men reported higher duration of use (Figure S4 in Multimedia Appendix 2). Men also reported higher duration of use for online gaming on tablets and laptops compared to women (Figures S5 and S6 in Multimedia Appendix 2). Men also reported higher duration of use for texting, emailing, and accessing the internet on tablets and social media on laptops, whereas women reported higher duration of use for texting, emailing, and internet use and video streaming on laptops. Further differences in duration of use for the different activities on each device by employment status, parental educational level, country of birth, and urbanicity of place of residence can be found in Figures S7 to S21 in Multimedia Appendix 2.

### Device Position

Device position near the eyes during use for both smartphones and tablets is shown in Figures4  5. Smartphone voice calls were performed primarily against the ear or in speaker mode. For smartphones and tablets, video calls and video streaming were performed predominantly in front of the eyes. However, music streaming was performed distinctly more in front of the eyes on tablets compared to smartphones, on which most of the time it was never or rarely performed in front of the eyes.

**Figure 5.**
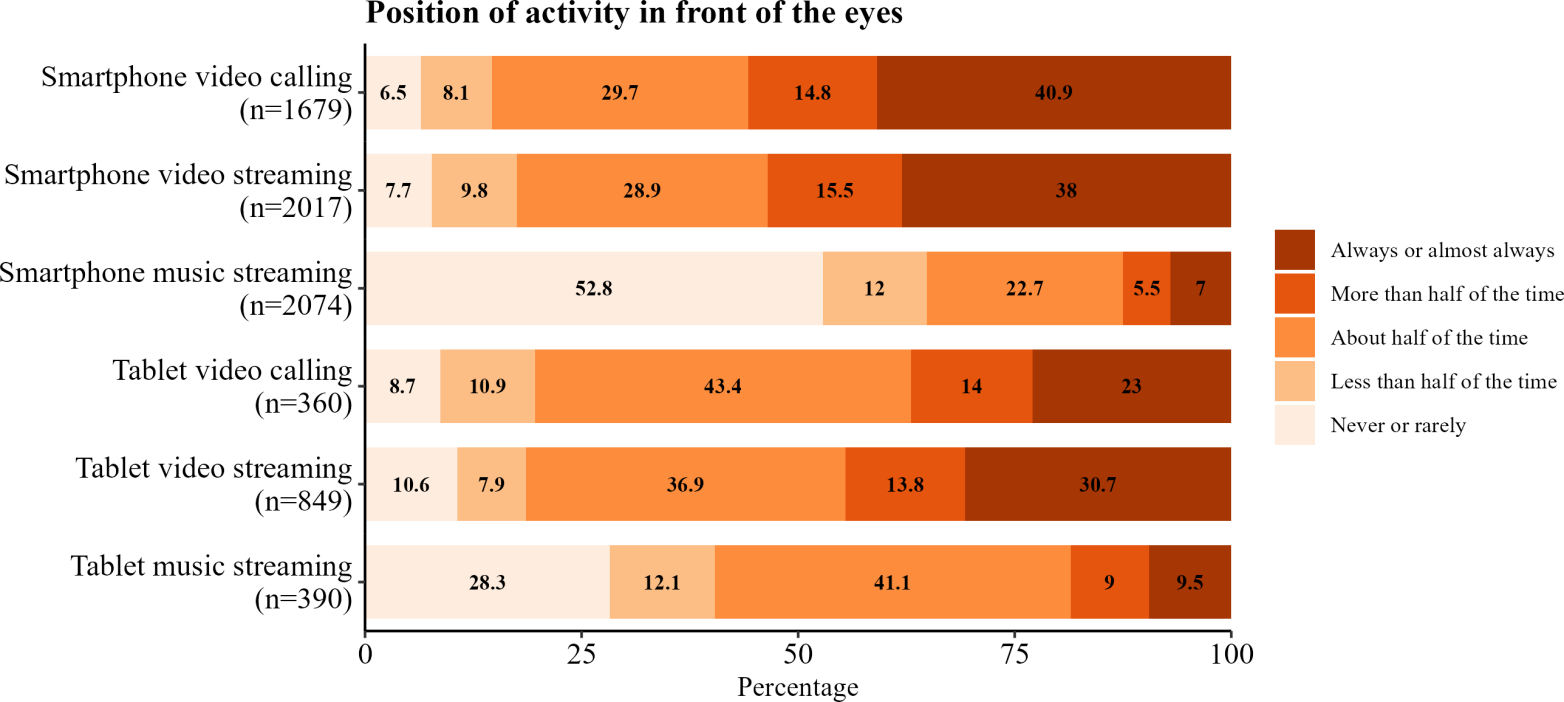
Frequencies of activities for the device position in front of the eyes using a smartphone and tablet by activity in young adults.

### Other Devices

Prevalence of use of other devices was 12.8% (510/4000) for gaming consoles and 3.5% (141/4000) for virtual reality glasses or augmented reality devices. Reported mean duration of use was 8.0 (SD 26.9) minutes per week for gaming consoles and 1.8 (SD 13.6) minutes per week for virtual reality glasses or augmented reality devices.

## Discussion

### Principal Findings

In this paper, we present novel data on the digital communication device use of young people in 4 European countries. Approximately 90% of the participants (3635/4000) reported regularly using smartphones, followed by almost 70% using laptops (2748/4000), 33% (1329/4000) using tablets, 23% (931/4000) using smartwatches or activity trackers, 12% (462/4000) using cordless phones, 12% (510/4000) using gaming consoles, and 3% (141/4000) using virtual reality glasses or augmented reality devices. Older age groups and women reported higher use across most devices. Device use also varied by country of residence. The most prevalent activities that participants reported engaging in on smartphones were social media use; voice calls; and texting, emailing, and internet use. Similar activities were observed on both tablets and laptops, with the most prevalent being video streaming; texting, emailing, and internet use; and social media. While we found variations in the duration of use across different socioeconomic and demographic characteristics, inconsistent patterns were observed across activities.

Our findings corroborate the ubiquitous use of smartphones by young people, with other similar studies reporting smartphone use prevalence above 90% [1,15,20-22]. Prevalence of tablet use was similar to that in previous findings of 32% for individuals aged 16 to 24 years in Europe [1]. However, at the same time, prevalences between countries appear to be remarkably different for tablet use. For example, in Germany, tablet use was reported at 42% compared to only 6% in Croatia for individuals aged 16 to 24 years in 2023 [1]. We observed similar laptop use in our participants compared to other findings on young people in Europe of 70% among individuals aged 18 to 29 years [1]. Other studies have reported regular use of cordless phones among 18% of young people in the Netherlands [23] and 15% of young people in Japan [24], and we found similar or slightly lower results. Regarding smartwatches and activity trackers, previous market studies have found similar results on wearable technology use of 27% in individuals aged 18 to 34 years [25] and 28% in individuals aged 18 to 24 years [26]. For gaming consoles, we found lower use compared to previous findings of 42% for individuals aged 16 to 24 years in Europe [27].

Previous research has shown that social media use among young people through digital communication devices is significantly higher than that among older adults [15,16,28,29]. Many studies have also focused on the public health impact of social media use either as the exposure itself (due to RF-EMF exposure or problematic use) or as a tool for the dissemination of health information and disease prevention [30]. In our study, social media was regularly engaged with on all types of devices. This reinforces the reality of how expansive social media is within the digital communication world, corroborating the fact that social media use is an activity that is imperative to investigate when conducting digital health research. Nonetheless, smartphones were still the device on which most participants in our study engaged with social media for longer periods, in accordance with previous findings [15,16], whereas participants spent more time using tablets and laptops for video streaming or music streaming.

Our results indicated that the ways in which young people use digital communication devices vary across certain demographic and socioeconomic variables. Previous literature has found that adolescents exhibit higher reliance on persistent and addictive smartphone use behavior and that, as they grow older, this reliance decreases [31,32]. In line with this, we observed that the youngest age group had higher duration of use of smartphones for voice calls, social media, and video streaming. However, the 2 oldest age groups reported the highest device use for tablets, laptops, and smartwatches or activity trackers. This could be due to more potential parental restrictions placed on those between the ages of 16 and 18 years.

In our study, women reported higher duration of use for almost all activities on smartphones, whereas men reported higher levels of online gaming. These findings appear to be in line with those of previous research that found excessive smartphone use in women and internet gaming disorder in men [31,33-36]. As gender plays a significant role as a social determinant of health, a considerable amount of research has been conducted surrounding the use of digital communication devices by gender. Results from these studies show differences in terms of which genders make greater use of digital communication devices and what activities they engage in. For example, some studies have reported that men have similar or higher levels of device use compared to women [32,37], whereas others report higher levels among women [1,15,25,38]. These inconsistent results are likely due to largely unexplained cultural factors that vary across countries or in certain groups.

While certain differences were found regarding the activities performed on each device according to employment status, inconsistent patterns were observed. We observed higher laptop use among those working or studying, which is likely the result of the uptake of technology use in both educational institutions and the workplace [39,40]. People born in the same country where the study took place also reported higher use of smartphones, laptops, and smartwatches or activity trackers. This could be related to income differences, as has been observed in previous studies [37,41], as migrant individual pay gaps in high-income countries can be significant [42]. Unfortunately, we did not collect information on participants’ income in this study. With increasing attention to the use of digital health services to support migrant groups and reduce health inequalities [43], the lower uptake of digital communication device use in this population still represents an impediment to accessing digital health services that needs to be addressed. Potential income differences could also be related to our results regarding parental educational level. Previous studies have found very little contribution of parental educational level to technology and phone use characteristics [13,44]. However, our results show that participants whose parents had the highest educational level reported the highest use of tablets, laptops, and smartwatches or activity trackers. These devices are expensive and might be perceived as less necessary outside school or work environments compared to smartphones, in line with previous research findings [21].

Interestingly, the country of residence was the only variable that differed in almost all digital communication device measures we explored. This potentially points toward a broader scope of the digital divide and cultural differences in information and communications technology [45,46]. It has been reported that this divide has been exacerbated by the COVID-19 pandemic [47] and that it has affected the patterns of use in digital devices [48,49]. It is likely that commonalities present in our data are supported by this underlying framework. Previous research has shown that the urban-rural divide in certain European Union and non–European Union countries can be significant in terms of internet access [50,51]. Place of residence showed little differences in terms of device use and duration of use among our participants.

We also reported the position of activities with respect to the participants’ body during use for smartphones and tablets. Smartphone position of use tended to be nearer the eyes compared to tablets for the activities we evaluated, although the overall results were still very similar. These data are of importance and particular use in epidemiological studies exploring RF-EMF exposure as the position of the device influences the level of RF-EMF exposure [52,53].

Due to the rapid evolution of technologies and their uses, one limitation of this study is that it only captured current technology, potentially restricting this study’s future applicability. In addition, we included a variety of activities that were relevant at the time of the survey but may have missed other applicable activities for each device. For future surveys, it will be important to update the activities or potentially use pilot surveys to ascertain the most current internet behaviors. Moreover, while we grouped together the activities of texting, sending pictures or emails, and internet browsing because they all produce a similar RF-EMF exposure to the brain and the body [54], this limits the specific interpretations of each activity from a general perspective. Participation in the survey was through an online platform, which may have introduced biases of self-selection based on interest in the topic and led to low response rates [55]. The method of data collection required that the individual answering the survey have access to the internet, missing out on potential participants who have limited or no access to the internet or devices. Additionally, convenience sampling was used, which may introduce selection bias. Comparing the distribution of parental educational level and country of birth with that of Eurostat, our population was slightly biased toward higher parental educational levels (37.3% in our study vs 32.7% in Eurostat with a higher educational level and 18.5% in our study vs 23.7% in Eurostat with a lower educational level) and local origin (87.9% in our study vs 84.2% in Eurostat) [56,57]. Furthermore, recall bias could be present in participants’ answers for duration of use and device position in the previous 3 months. However, recent research findings have shown a moderate correlation between the reported and measured smartphone use of young people [58,59] and that low use is usually underestimated, whereas high use is overestimated.

While we acknowledge that our study has some limitations, it also has numerous strengths. We performed this survey on a large sample of 4000 people, allowing for better representation of the younger population. In addition, participants of this study lived in 4 different countries in Europe, enabling a better understanding of country-level effects. Very few studies to date have also collected information on detailed activity engagement, duration of use, and position of activity, in particular in this age range, accentuating the novelty of our study. As young people have been described as hard to reach for health promotion and disease prevention strategies [15], identifying the avenues through which to engage these individuals, especially from a technological perspective, may prove useful for public health researchers.

### Conclusions

We have characterized the use of digital communication devices and identified differences by socioeconomic and demographic characteristics. Prevalence of device use by age, gender, and country of residence varied among our population. Through incorporating detailed information about specific digital devices, we were able to present a comprehensive assessment of how young people use their devices by frequency of use, activity engagement, and duration. As current research is fragmented by device or is out of date, establishing a basis for more up-to-date information on digital communication devices that can be used by public health researchers is of great importance. Technology is rapidly evolving (eg, the 5G rollout), making it difficult to anticipate what is around the corner, both as an exposure and as a tool for our health. It is essential for researchers to regularly assess technology and digital device use to understand their current states. Future research will consist of repeating this study after 2 years and investigating changes of use during this time.

## Supplementary material

10.2196/76767 Multimedia Appendix 1 GOLIAT (“5G exposure, causal effects, and risk perception through citizen engagement”) project questionnaire.

10.2196/76767 Multimedia Appendix 2 Overview of the collected information and frequencies of digital communication device use by socioeconomic and demographic characteristics.

10.2196/76767 Checklist 1 CHERRIES checklist.

## Abbreviations

CHERRIES: Checklist for Reporting Results of Internet E-Surveys
GOLIAT: 5G exposure, causal effects, and risk perception through citizen engagement
RF-EMF: radiofrequency electromagnetic field
