# Supplementary material for: Characterizing Digital Communication Device Use Among Young People From 4 European Countries: Cross-Sectional Survey Study
Source: J Med Internet Res. 2025 Dec 23;27:e76767. doi: 10.2196/76767 (PMC12724067; doi:10.2196/76767)
Supplement: Multimedia Appendix 1 [file jmir-v27-e76767-s001.docx]

**Questionnaire – Task 1.1 GOLIAT**

**A. Personal Information**

1. **What is your age?**|__|__|__|
2. **What is your gender?**

- Female
- Male
- Other

1. **What is your country of birth?** Country dropdown
2. **What is the country of birth of your mother?** Country dropdown
3. **What is the highest level of formal education your mother completed? Country specific**

- Primary school
- Secondary school
- Vocational education
- College
- University
- Postgraduate degree

1. **What is the country of birth of your father?** Country dropdown
2. **What is the highest level of formal education your father completed? Country specific**

- Primary school
- Secondary school
- Vocational education
- College
- University
- Postgraduate degree

1. **How many rooms do you have in your house?** |__|__|__|
2. **How many people live in your house?** |__|__|__|
3. **Which one of the following best describes your current employment status**? More than one option

- Student
- Working full time
- Working part-time/Seasonal/occasional work: % time over a year _______________
- Currently not employed
- Housewife/houseman (unpaid)
- Carer for children, elderly or person with illness or disability (unpaid)
- Long-term sick leave/disability
- Other, please specify:____________________________
  1. **If working, what describes best your working situation?**
- Office
- Mostly outdoor working
- Office and outdoor
- Home-base job
- Industrial setting (e.g. factory)
- Hospital
- Public transport
- Transport industry
- Craftsman/craftswoman
- Hospitality (restaurant/bar/hotel)
- Research/laboratory
- Shop, sales
- Working with mobile phone base stations and other RF-EMF transmitters
- Other

1. **How would you describe your place of residence?**

- Village (< 5.000 residents)
- Town or suburbs of a large city (5.000-100.000 residents)
- City (> 100.000 residents)

1. **In a typical week (outside of extraordinary periods such as holidays, illness, COVID-19 quarantine, etc.) how many days a week do you engage in vigorous physical activity for at least 60 minutes a day?**Consider the total! For example: if on the same day your child runs for 30 minutes and cycles twice for 15 minutes, the total amount of physical activity done that day will be 60 minutes.

|__| Days per week

1. **Which transport(s) do you use typically on a school day/working day and for how long in total per day? In the table below, select the transport and time range that fits best for you** (You can select more than one if you take multiple transports)

|  | 5 minutes or less | 6-10 minutes | 11-20 minutes | 21-30 minutes | More than 30 minutes |
| --- | --- | --- | --- | --- | --- |
| Walking |  |  |  |  |  |
| Bicycle or scooter (traditional or electric) |  |  |  |  |  |
| Bus |  |  |  |  |  |
| Metro |  |  |  |  |  |
| Train |  |  |  |  |  |
| Tram |  |  |  |  |  |
| Car |  |  |  |  |  |
| Other (e.g. motorbike) |  |  |  |  |  |

**14. How many days per week are you typically commuting to work/school?**

|__| Days per week

**B. Use of mobile devices**

1. **Which of the following devices do you use on a regular basis? (**By regular basis we mean at least once a week in the past 3 months) You can choose more than one option from the list below:

- Smartphone
- Tablet
- Laptop
- Cordless phone [Cordless phones are portable phones you use at home (that is, on a landline)]
- Smart watches/Activity trackers (e.g., Fitbit, Apple watch, Samsung Galaxy watch)
- Virtual glasses
- Other mobile devices (e.g., Virtual glasses, Portable gaming consoles, Augmented reality)
- None

**B.1. Smartphone use**

This group of questions should only appear if selected “Smartphone” on question 1 section B. Use of mobile devices

1. **Regarding the mobile phone that you use the most**:
   1. **Which is the brand and the model (e.g. Brand: Samsung Galaxy, Model: S21)?**

Brand __________________________________ Model____________________________________

- 1. **Is the phone 5G compatible?**
- No
- Yes
- I don’t know
  1. **Which is the mobile phone operator?** _______________________________________________
  2. **Do you have 5G Network services included in your contract?**
- No
- Yes
- I don’t know

**The following questions refer to how you handle your smartphone when you are NOT using it during the day**

1. **Where do you usually carry/store the smartphone when you are not using it?**

|  | Public transports | Car | Walking | Elsewhere outdoors (e.g., doing sports) |
| --- | --- | --- | --- | --- |
| In my hand |  |  |  |  |
| In my shirt/jacket’s pocket |  |  |  |  |
| In my trousers’ pocket |  |  |  |  |
| In a neck pouch for phones |  |  |  |  |
| Arm phone holder |  |  |  |  |
| Somewhere else, not in contact with my body |  |  |  |  |

1. **When you are not using your smartphone, it is usually** (more than one option can be selected):

- Wi-Fi turned on
- Mobile phone data turned on
- Bluetooth turned on
- Wi-Fi, mobile phone data, AND Bluetooth turned OFF
- I turn my phone off or put it in flight mode

**The following questions refer to your smartphone usage**

1. **Which of the following activities do you regularly perform using a smartphone?** (By regularly we mean at least once a week in the past 3 months). *You can choose more than one option from the list below*

- Voice calls (with or without an app – e.g., WhatsApp, Hangouts, Skype, etc.)
- Internet video calls (e.g., WhatsApp, FaceTime, Skype, Zoom)
- Voice messages (i.e., voice notes sent via an App)
- Send Videos (NOT sending a link to a video)
- Texting or sending pictures (via SMS or via an app WhatsApp, Hangouts, iMessage, Viber, Telegram, etc.), Sending e-mails and/or Internet browsing (including online shopping)
- Using social media –watching videos (e.g., Instagram, TikTok), scrolling social media, chatting (Facebook/Instagram, commenting posts) and uploading pictures/videos (e.g., Facebook/Instagram)
- Online video streaming (e.g., Netflix, HBO, Disney+, YouTube)
- Online music streaming/online listening to podcasts
- Online gaming (i.e., games/apps that require an internet connexion)
- As an hotspot (i.e., sharing internet connection)

1. **Voice calls (with or without an app, WhatsApp, Hangouts, Skype, etc.) (**This group of questions should only appear if the function was selected in Q4 group B1)
   1. **For how long do you call on average per day (incoming and outgoing calls)?**

|  | During the week  (Monday-Friday) | During the weekend  (Saturday-Sunday) |
| --- | --- | --- |
| 5 minutes or less |  |  |
| 6-15 minutes per day |  |  |
| 16-30 minutes per day |  |  |
| 31-60 minutes per day |  |  |
| 1-2 hours per day |  |  |
| 2-4 hours per day |  |  |
| More than 4 hours per day |  |  |

- 1. **How do you perform your calls?**

|  | Never or rarely | Less than half of the time | About half of the time | More than half of the time | Always or almost always |
| --- | --- | --- | --- | --- | --- |
| Holding my phone against my ear |  |  |  |  |  |
| Holding my phone in front of my eyes, in speaker mode. |  |  |  |  |  |
| Elsewhere |  |  |  |  |  |

- 1. **Now, take your smartphone and pretend to make a call by holding the phone against your ear, as you usually do. To which ear have you pressed the phone?**
- Left ear
- Right ear

1. **Internet video calls (**This group of questions should only appear if the function was selected in Q4 group B1)
   1. **For how long do you video call using an app (e.g., WhatsApp, Skype, FaceTime, etc.) on average per day?**

|  | During the week  (Monday-Friday) | During the weekend  (Saturday-Sunday) |
| --- | --- | --- |
| 5 minutes or less |  |  |
| 6-15 minutes per day |  |  |
| 16-30 minutes per day |  |  |
| 31-60 minutes per day |  |  |
| 1-2 hours per day |  |  |
| More than 2 hours per day |  |  |

- 1. **How do you perform your video calls?**

|  | Never or rarely | Less than half of the time | About half of the time | More than half of the time | Always or almost always |
| --- | --- | --- | --- | --- | --- |
| Holding my phone in front of my eyes |  |  |  |  |  |
| Elsewhere |  |  |  |  |  |

1. **Voice messages or Send Videos** (This group of questions should only appear if the function was selected in Q4 group B1)
   1. **How many voice messages or videos do you send on average per day?**

|  | During the week  (Monday - Friday) | During the weekend  (Saturday-Sunday) |
| --- | --- | --- |
| Less than one message/video per day |  |  |
| 1-5 messages/videos per day |  |  |
| 6-10 messages/videos per day |  |  |
| 11-20 message/video per day |  |  |
| More than 20 message/video per day |  |  |

1. **Texting, send e-mails or Internet browsing** (This group of questions should only appear if the function was selected in Q4 group B1)
   1. **How much time on average per day do you spend sending text messages (SMS and/or WhatsApp, Hangouts, iMessage, Viber, Telegram, etc.), browsing on the internet (e.g., News websites or apps, online shopping, Google, Wikipedia) or sending e-mails, using your smartphone?**

|  | During the week  (Monday-Friday) | During the weekend  (Saturday-Sunday) |
| --- | --- | --- |
| 10 minutes or less per day |  |  |
| 11-30 minutes per day |  |  |
| 31-60 minutes per day |  |  |
| 1-2 hours per day |  |  |
| 2-4 hours per day |  |  |
| More than 4 hours per day |  |  |

1. **Use of social media** (This group of questions should only appear if the function was selected in Q4 group B1)

**NOTE**: by Social Media we mean websites and applications that enable users to create and share content or to participate in social networking (Facebook, TikTok, Instagram, Pinterest, Snapchat, Twitter, LinkedIn).

**DO NOT** consider YouTube as this is included as online video streaming function; **DO NOT** consider message Apps like WhatsApp, Hangouts, iMessage, Viber or Telegram, as these are included in the Voice/video messages and the texting functions.

- 1. **How much time on average per day do you spend on social media, using a smartphone? This includes watching videos (e.g., Instagram, TikTok), chatting (e.g., Facebook/Instagram online chats, commenting posts), scrolling through social media and/or uploading pictures/videos on social media.**

**Hint**: You can consult your smartphone. Some apps, e.g. Instagram, give you an average usage time per day

|  | During the week  (Monday-Friday) | During the weekend  (Saturday-Sunday) |
| --- | --- | --- |
| 10 minutes or less per day |  |  |
| 11-30 minutes per day |  |  |
| 31-60 minutes per day |  |  |
| 1-2 hours per day |  |  |
| 2-4 hours per day |  |  |
| More than 4 hours per day |  |  |

- 1. **When using social media, how often do you perform the following activities?**

|  | Never or Rarely | Less than half of the time | About half of the time | More than half of the time | Always or almost always |
| --- | --- | --- | --- | --- | --- |
| Scrolling on social media (e.g., Instagram/Facebook/Pinterest) |  |  |  |  |  |
| Watching videos/Reels (e.g., TikTok, Instagram) |  |  |  |  |  |
| Chatting (e.g. online chat, commenting posts, etc.) |  |  |  |  |  |
| Uploading videos/pictures |  |  |  |  |  |

1. **Online video streaming** (This group of questions should only appear if the function was selected in Q4 group B1

**Hint:** Video streaming is a continuous transmission of video files from a server to a client (Examples: Netflix, HBO, AppleTV, Disney+, YouTube, etc.)

- 1. **How much time on average per day do you spend online video streaming using a smartphone?**

|  | During the week  (Monday-Friday) | During the weekend  (Saturday-Sunday) |
| --- | --- | --- |
| 10 minutes or less per day |  |  |
| 11-30 minutes per day |  |  |
| 31-60 minutes per day |  |  |
| 1-2 hours per day |  |  |
| 2-4 hours per day |  |  |
| More than 4 hours per day |  |  |

- 1. **Where do you place the smartphone when online video streaming?**

|  | Never or Rarely | Less than half of the time | About half of the time | More than half of the time | Always or almost always |
| --- | --- | --- | --- | --- | --- |
| In front of my eyes |  |  |  |  |  |
| Elsewhere |  |  |  |  |  |

1. **Online music streaming/Online listening to podcasts** (This group of questions should only appear if the function was selected in Q4 group B1)
   1. **How much time on average per day do you spend streaming music or podcasts online using a smartphone?**

|  | During the week  (Monday-Friday) | During the weekend  (Saturday-Sunday) |
| --- | --- | --- |
| 10 minutes or less per day |  |  |
| 11-30 minutes per day |  |  |
| 31-60 minutes per day |  |  |
| 1-2 hours per day |  |  |
| 2-4 hours per day |  |  |
| More than 4 hours per day |  |  |

- 1. **Where do you place the smartphone when streaming music or podcasts online?**

|  | Never or Rarely | Less than half of the time | About half of the time | More than half of the time | Always or almost always |
| --- | --- | --- | --- | --- | --- |
| In front of my eyes |  |  |  |  |  |
| Elsewhere |  |  |  |  |  |

1. **Online gaming on your mobile phone** (This group of questions should only appear if the function was selected in Q4 group B1)

**Hint:** By online gaming we refer to any website or mobile phone App that requires an internet connection

- 1. **How much time do you spend on average per day playing online games on your smartphone?**

|  | During the week  (Monday - Friday) | During the weekend  (Saturday-Sunday) |
| --- | --- | --- |
| 10 minutes or less per day |  |  |
| 11-30 minutes per day |  |  |
| 30-60 minutes per day |  |  |
| 1-2 hours per day |  |  |
| 2-4 hours per day |  |  |
| More than 4 hours per day |  |  |

- 1. **Name the top 3 games you currently play on your smartphone?** ______________________

1. **Wi-Fi connection**

**12.1 How often do you usually connect your smartphone to the Internet via Wi-Fi (vs. mobile phone data) when you are…**

|  | Never or rarely | Less than half of the time | About half of the time | More than half of the time | Always or almost always |
| --- | --- | --- | --- | --- | --- |
| At home |  |  |  |  |  |
| Commuting |  |  |  |  |  |
| At school/work |  |  |  |  |  |

1. **Hotspot (i.e., sharing internet connection)** (This group of questions should only appear if the function was selected in Q4 group B1)
   1. **For how long is your smartphone sharing an internet connection with other devices (i.e., serving as a hotspot)?** Select the most appropriate average per week.

- 10 minutes or less per week
- 11-30 minutes per week
- 30-60 minutes per week
- 1-2 hours per week
- 2-4 hours per week
- More than 4h per week

1. **The following questions refer to the use of Bluetooth connected HEADPHONES while using your smartphone** (This question appear if any smartphone use is selected)
   1. **When using your smartphone either to call, stream videos, listen to music/podcasts or play online games, how often do you use headphones or AirPods connected via Bluetooth?**

- Always or almost always
- More than half of the time
- About half of the time
- Less than half of the time
- Never or Rarely
  1. **In each ear do you mostly use your Bluetooth connected phones/AirPods?**
- Right ear
- Left year
- Both ears, simultaneously

**B.2. Tablet use**

This group of questions should only appear if selected “Tablet” on question 1 section B. Use of mobile devices

1. **Which of the following activities do you perform regularly using a tablet?** By regularly we mean at least once a week in the past 3 months.

- Internet video calls (e.g., Skype, Zoom)
- Sending emails and/or Internet browsing (including online shopping)
- Using social media – This includes watching videos (e.g., Instagram, TikTok), chatting (e.g., Facebook/Instagram online chat, commenting posts), scrolling through social media, uploading pictures/videos on social media
- Online video streaming (e.g., Netflix, HBO, Disney+, YouTube)
- Online music streaming/online listening to podcasts
- Online gaming (i.e., games/apps that require an internet connexion)

1. **Internet video calls** (This group of questions should only appear if the function was selected in Q1 group B2)
   1. **For how long, on average per day, do you video call using a tablet?**

|  | During the week  (Monday-Friday) | During the weekend  (Saturday-Sunday) |
| --- | --- | --- |
| 5 minutes or less per day |  |  |
| 6-15 minutes per day |  |  |
| 16-30 minutes per day |  |  |
| 31-60 minutes per day |  |  |
| 1-2 hours per day |  |  |
| More than 2 hours per day |  |  |

- 1. **Where do you place the tablet when video calling?**

|  | Never or rarely | Less than half of the time | About half of the time | More than half of the time | Always or almost always |
| --- | --- | --- | --- | --- | --- |
| I hold it in front of my eyes |  |  |  |  |  |
| Elsewhere |  |  |  |  |  |

1. **Send e-mails or Internet browsing** (This group of questions should only appear if the function was selected in Q1 group B2)
   1. **How much time on average per day do you spend sending text messages (WhatsApp, Hangouts, Viber, Telegram, etc.), browsing on the internet (e.g., News websites or apps, online shopping, Google, Wikipedia) or sending e-mails, using a tablet?**

|  | During the week  (Monday-Friday) | During the weekend  (Saturday-Sunday) |
| --- | --- | --- |
| 10 minutes or less per day |  |  |
| 11-30 minutes per day |  |  |
| 31-60 minutes per day |  |  |
| 1-2 hours per day |  |  |
| 2-4 hours per day |  |  |
| More than 4 hours per day |  |  |

1. **Use of social media** (This group of questions should only appear if the function was selected in Q1 group B2)
   1. **How much time on average per day do you spend on social media, using a tablet?**

This includes watching videos (e.g., Instagram, TikTok), chatting (e.g., Facebook/Instagram online chat, commenting posts), scrolling through social media and/or uploading pictures/videos on social media.

|  | During the week  (Monday-Friday) | During the weekend  (Saturday-Sunday) |
| --- | --- | --- |
| 10 minutes or less per day |  |  |
| 11-30 minutes per day |  |  |
| 31-60 minutes per day |  |  |
| 1-2 hours per day |  |  |
| 2-4 hours per day |  |  |
| More than 4 hours per day |  |  |

- 1. **When using social media on a tablet, how often do you perform the following activities?**

|  | Never or Rarely | Less than half of the time | About half of the time | More than half of the time | Always or almost always |
| --- | --- | --- | --- | --- | --- |
| Scrolling on social media (e.g., Instagram/Facebook/ Pinterest) |  |  |  |  |  |
| Watching videos/Reels (e.g., TikTok, Instagram) |  |  |  |  |  |
| Chatting (e.g., online chat, commenting posts, etc.) |  |  |  |  |  |
| Uploading videos/pictures |  |  |  |  |  |

1. **Online video streaming** (This group of questions should only appear if the function was selected in Q1 group B2)

**Hint:** Video streaming is a continuous transmission of video files from a server to a client (Examples: Netflix, HBO, AppleTV, Disney+, YouTube, etc.)

- 1. **How much time on average per day do you spend online video streaming using a tablet?**

|  | During the week  (Monday-Friday) | During the weekend  (Saturday-Sunday) |
| --- | --- | --- |
| 10 minutes or less per day |  |  |
| 11-30 minutes per day |  |  |
| 31-60 minutes per day |  |  |
| 1-2 hours per day |  |  |
| 2-4 hours per day |  |  |
| More than 4 hours per day |  |  |

- 1. **Where do you place the tablet when online video streaming?**

|  | Never or Rarely | Less than half of the time | About half of the time | More than half of the time | Always or almost always |
| --- | --- | --- | --- | --- | --- |
| In front of my eyes |  |  |  |  |  |
| Elsewhere |  |  |  |  |  |

1. **Online music streaming/Online listening to podcasts** (This group of questions should only appear if the function was selected in Q1 group B2)
   1. **How much time on average per day do you spend streaming music or podcasts online using a tablet?**

|  | During the week  (Monday-Friday) | During the weekend  (Saturday-Sunday) |
| --- | --- | --- |
| 10 minutes or less per day |  |  |
| 11-30 minutes per day |  |  |
| 31-60 minutes per day |  |  |
| 1-2 hours per day |  |  |
| 2-4 hours per day |  |  |
| More than 4 hours per day |  |  |

- 1. **Where do you place your tablet when streaming music or podcasts online?**

|  | Never or Rarely | Less than half of the time | About half of the time | More than half of the time | Always or almost always |
| --- | --- | --- | --- | --- | --- |
| In front of my eyes |  |  |  |  |  |
| Elsewhere |  |  |  |  |  |

1. **Online gaming** (This group of questions should only appear if the function was selected in Q1 group B2)

**Hint:** By online gaming we refer to any website or mobile phone App that requires an internet connection

- 1. **How much time do you spend on average per day playing online games on a tablet?**

|  | During the week  (Monday - Friday) | During the weekend  (Saturday-Sunday) |
| --- | --- | --- |
| 10 minutes or less per day |  |  |
| 11-30 minutes per day |  |  |
| 30-60 minutes per day |  |  |
| 1-2 hours per day |  |  |
| 2-4 hours per day |  |  |
| More than 4 hours per day |  |  |

- 1. **Name the top 3 games you currently play on your tablet?** ___________________________________

1. **The following questions refer to the use of Bluetooth connected HEADPHONES while using the tablet** (This question appear if any tablet use is selected)
   1. **When using your tablet to either video call, stream videos, listen to music/podcasts or play online games, how often do you use headphones or AirPods connected via Bluetooth?**

- Always or almost always
- More than half of the time
- About half of the time
- Less than half of the time
- Never or Rarely
  1. **In each ear do you mostly use your Bluetooth connected phones/AirPods?**
- Right ear
- Left year
- Both ears, simultaneously

**B.3. Laptop**

This group of questions should only appear if selected “Laptop” on question 1 section B. Use of mobile devices

1. **How often are you connected to Wi-Fi when using your laptop?**

- Never or Rarely – I use an Ethernet cable for internet access, go to next section B4
- Sometimes, go to question 2
- Always or almost always, go to question 2

1. **Which of the following activities do you perform on a regular basis when using a laptop?** (By regular basis, we mean at least once a week in the past 3 months).

- Internet video calls (e.g., Skype, Zoom, Google Meets)
- Using social media – Watching videos (e.g., Instagram, TikTok), chatting (e.g., Facebook/Instagram, commenting posts), scrolling social media, uploading pictures/videos
- Send e-mails and/or Internet browsing (including online shopping)
- Online video streaming (e.g., Netflix, HBO, Disney+, YouTube)
- Online music streaming/online listening to podcasts
- Online gaming (i.e., games/apps that require an internet connexion)

1. **Internet video calls** (This group of questions should only appear if the function was selected in Q2 group B3)
   1. **For how long on average per day do you video call using a laptop?**

|  | During the week  (Monday-Friday) | During the weekend  (Saturday-Sunday) |
| --- | --- | --- |
| 5 minutes or less per day |  |  |
| 6-15 minutes per day |  |  |
| 16-30 minutes per day |  |  |
| 31-60 minutes per day |  |  |
| 1-2 hours per day |  |  |
| More than 2 hours per day |  |  |

1. **Sending e-mails or Internet browsing** (This group of questions should only appear if the function was selected in Q2 group B3)
   1. **How much time do you spend on average per day sending-emails or/and browsing on the internet using a laptop?**

|  | During the week  (Monday - Friday) | During the weekend  (Saturday-Sunday) |
| --- | --- | --- |
| 10 minutes or less per day |  |  |
| 10-30 minutes per day |  |  |
| 30-60 minutes per day |  |  |
| 1-2 hours per day |  |  |
| More than 2 hours per day |  |  |

1. **Use of Social Media** (This group of questions should only appear if the function was selected in Q2 group B3)
   1. **How much time do you spend on average per day on social media using a laptop?**

This includes watching videos (e.g., Instagram, TikTok), chatting (e.g., Facebook/Instagram chat, commenting posts), scrolling through social media and/or uploading pictures/videos on social media.

|  | During the week  (Monday - Friday) | During the weekend  (Saturday-Sunday) |
| --- | --- | --- |
| 10 minutes or less per day |  |  |
| 11-30 minutes per day |  |  |
| 30-60 minutes per day |  |  |
| 1-2 hours per day |  |  |
| 2-4 hours per day |  |  |

- 1. **When using social media on a laptop, how often do you perform the following activities?**

|  | Never or Rarely | Less than half of the time | About half of the time | More than half of the time | Always or almost always |
| --- | --- | --- | --- | --- | --- |
| Scrolling on social media (e.g., Instagram/ Facebook/ Pinterest) |  |  |  |  |  |
| Watching videos/Reels (e.g., TikTok, Instagram) |  |  |  |  |  |
| Chatting (e.g., online chat, commenting posts, etc.) |  |  |  |  |  |
| Uploading videos/pictures |  |  |  |  |  |

1. **Online Video Streaming** (This group of questions should only appear if the function was selected in Q2 group B3)
   1. **How much time on average per day do you spend online video streaming (e.g., Netflix, HBO, Diney+) using a laptop?**

|  | During the week  (Monday-Friday) | During the weekend  (Saturday-Sunday) |
| --- | --- | --- |
| 10 minutes or less per day |  |  |
| 11-30 minutes per day |  |  |
| 31-60 minutes per day |  |  |
| 1-2 hours per day |  |  |
| 2-4 hours per day |  |  |
| More than 4 hours per day |  |  |

1. **Online Music Streaming** (This group of questions should only appear if the function was selected in Q2 group B3)
   1. **How much time on average per day do you spend streaming music or podcasts online using a laptop?**

|  | During the week  (Monday-Friday) | During the weekend  (Saturday-Sunday) |
| --- | --- | --- |
| 10 minutes or less per day |  |  |
| 11-30 minutes per day |  |  |
| 31-60 minutes per day |  |  |
| 1-2 hours per day |  |  |
| 2-4 hours per day |  |  |
| More than 4 hours per day |  |  |

- 1. **Name the top 3 games you currently play on your laptop**? ________________________________

1. **Online Gaming** (This group of questions should only appear if the function was selected in Q2 group B3)
   1. **How much time do you spend on average per day playing online games on a laptop?**

|  | During the week  (Monday - Friday) | During the weekend  (Saturday-Sunday) |
| --- | --- | --- |
| 10 minutes or less per day |  |  |
| 11-30 minutes per day |  |  |
| 30-60 minutes per day |  |  |
| 1-2 hours per day |  |  |
| 2-4 hours per day |  |  |
| More than 4 hours per day |  |  |

- 1. **Name the top 3 games you currently play on your laptop?** ________________________________
  2. **How often are you connected to Wi-Fi when playing online games on a laptop?**
- Never or Rarely
- Less than half of the time
- About half of the time
- More than half of the time
- Always or almost always

1. **The following questions refer to the use of Bluetooth connected HEADPHONES while using the laptop** (This question appear if any tablet use is selected)
   1. **When using your laptop either to video call, stream videos, listen to music/podcasts or play online games, how often do you use headphones or AirPods connected via Bluetooth?**

- Always or almost always
- More than half of the time
- About half of the time
- Less than half of the time
- Never or Rarely
  1. **In which ear do you mostly use your Bluetooth connected phones/AirPods?**
- Right ear
- Left year
- Both ears, simultaneously

**B4. Cordless phones**

This group of questions should only appear if selected “Cordless phones” on question 1 section B. Use of mobile devices

1. **For how long do you call on average per week?**

|  | During the week  (Monday-Friday) | During the weekend  (Saturday-Sunday) |
| --- | --- | --- |
| 5 minutes or less per day |  |  |
| 6-15 minutes per day |  |  |
| 16-30 minutes per day |  |  |
| 31-60 minutes per day |  |  |
| 1-2 hours per day |  |  |
| More than 2 hours per day |  |  |

1. **How do you perform your calls?**

|  | Never or rarely | Less than half of the time | About half of the time | More than half of the time | Always or almost always |
| --- | --- | --- | --- | --- | --- |
| Holding my phone against my ear |  |  |  |  |  |
| Holding my phone in front of my eyes, in speaker mode. |  |  |  |  |  |
| Elsewhere, in speaker mode |  |  |  |  |  |

**B5. Smart watch/Activity tracker**

This group of questions should only appear if selected “Smart watch/Activity tracker” on question B

**How often do you use your smart watch?**

- Once per week
- 1-3 days per week
- 4-5 days per week
- Every day

1. **How often is your smart watch connected to your phone via Bluetooth?**

- Never or Rarely
- Less than half of the time
- About half of the time
- More than half of the time
- Always or almost always

1. **In which wrist do you use your smart watch?**

- Right wrist
- Left wrist

1. **Do you sleep with your smart watch/activity tracker?**

- Yes
- No

**B7. Other devices**

This group of questions should only appear selected “Other mobile devices” (e.g., Virtual glasses, Portable gaming consoles, Augmented reality) on question 1 section B. Use of mobile devices

1. **Do you use another device?**

- No
- Yes
  1. **If yes, which one?** ____________________________________________________
  2. **For how long do you use this device?**
  - 5 minutes or less per week
  - 6-15 minutes per week
  - 16-30 minutes per week
  - 31-60 minutes per week
  - More than 60 minutes per week

1. **Do you use another device?**

- No
- Yes
  1. **If yes, which one?** ______________________________________________________________
  2. **For how long do you use this device?**
  - 5 minutes or less per week
  - 6-15 minutes per week
  - 16-30 minutes per week
  - 31-60 minutes per week
  - More than 60 minutes per week

1. **Do you use another device?**

- No
- Yes
  1. **If yes, which one?** _____________________________________________________________
  2. **For how long do you use this device?**
  - 5 minutes or less per week
  - 6-15 minutes per week
  - 16-30 minutes per week
  - 31-60 minutes per week
  - More than 60 minutes per week
