# Supplementary material for: Characterizing Digital Communication Device Use Among Young People From 4 European Countries: Cross-Sectional Survey Study
Source: J Med Internet Res. 2025 Dec 23;27:e76767. doi: 10.2196/76767 (PMC12724067; doi:10.2196/76767)
Supplement: Multimedia Appendix 2 [file jmir-v27-e76767-s002.docx]

## Table of Contents

[Table S1. Overview of collected information by device and activity 2](#_Toc192586707)

[Text S1. Quota Information 2](#_Toc216258876)

[Figure S1. Frequencies of smartphone activity duration per day by age group and activity in young adults. 3](#_Toc192586914)-5

[Figure S2. Frequencies of tablet activity duration per day by age group and activity in young adults. 6](#_Toc192586915)-7

[Figure S3. Frequencies of laptop activity duration per day by age group and activity in young adults. 8](#_Toc192586916)-9

[Figure S4. Frequencies of smartphone activity duration per day by gender and activity in young adults. 10](#_Toc192586917)-12

[Figure S5. Frequencies of tablet activity duration per day by gender and activity in young adults. 13](#_Toc192586918)-14

[Figure S6. Frequencies of laptop activity duration per day by gender and activity in young adults. 15](#_Toc192586919)-16

[Figure S7. Frequencies of smartphone activity duration per day by employment status and activity in young adults. 17](#_Toc192586920)-19

[Figure S8. Frequencies of tablet activity duration per day by employment status and activity in young adults. 20](#_Toc192586921)-21

[Figure S9. Frequencies of laptop activity duration per day by employment status and activity in young adults. 22](#_Toc192586922)-23

[Figure S10. Frequencies of smartphone activity duration per day by highest parental education and activity in young adults. 24](#_Toc192586923)-26

[Figure S11. Frequencies of tablet activity duration per day by highest parental education and activity in young adults. 27](#_Toc192586924)-28

[Figure S12. Frequencies of laptop activity duration per day by highest parental education and activity in young adults. 29](#_Toc192586925)-30

[Figure S13. Frequencies of smartphone activity duration per day by country of birth and activity in young adults. 31](#_Toc192586926)-33

[Figure S14. Frequencies of tablet activity duration per day by country of birth and activity in young adults. 34](#_Toc192586927)-35

[Figure S15. Frequencies of laptop activity duration per day by country of birth and activity in young adults. 36](#_Toc192586928)-37

[Figure S16. Frequencies of smartphone activity duration per day by country of residence and activity in young adults. 38](#_Toc192586929)-40

[Figure S17. Frequencies of tablet activity duration per day by country of residence and activity in young adults. 41](#_Toc192586930)-42

[Figure S18. Frequencies of laptop activity duration per day by country of residence and activity in young adults. 43](#_Toc192586931)-44

[Figure S19. Frequencies of smartphone activity duration per day by urbanicity and activity in young adults. 45](#_Toc192586932)-47

[Figure S20. Frequencies of tablet activity duration per day by urbanicity and activity in young adults. 48](#_Toc192586933)-49

[Figure S21. Frequencies of laptop activity duration per day by urbanicity and activity in young adults. 50](#_Toc192586934)-51

**Table S1. Overview of collected information by device and activity.**

|  | **Smartphone** | | **Tablet** | | **Laptop** | |
| --- | --- | --- | --- | --- | --- | --- |
| Activity | Time | Position | Time | Position | Time | Position |
| Voice calls | x | x |  |  |  |  |
| Internet video calls | x | x | x | x | x |  |
| Voice messages | x |  |  |  |  |  |
| Send Videos | x |  |  |  |  |  |
| Texting or sending pictures | x |  | x |  | x |  |
| Social media | x |  | x |  | x |  |
| Online video streaming | x | x | x | x | x |  |
| Online music streaming/podcasts | x | x | x | x | x |  |
| Online gaming | x |  | x |  | x |  |
| Hotspot | x |  |  |  |  |  |

Text S1. Quota Information

Noninterlocking quotes were administered for gender, age group, and place of residence. Gender quota percentages were 50% for men and 50% for women. For age group, quotas percentages were 30% for 16-18 years, 40% for 19-22 years, and 30% for 23-25 years. For place of residence, quota percentages were 40% for city [> 100,000 residents], 40% for towns or suburbs [10,000-100,000 residents], and 20% for rural [< 10,000 residents].

**
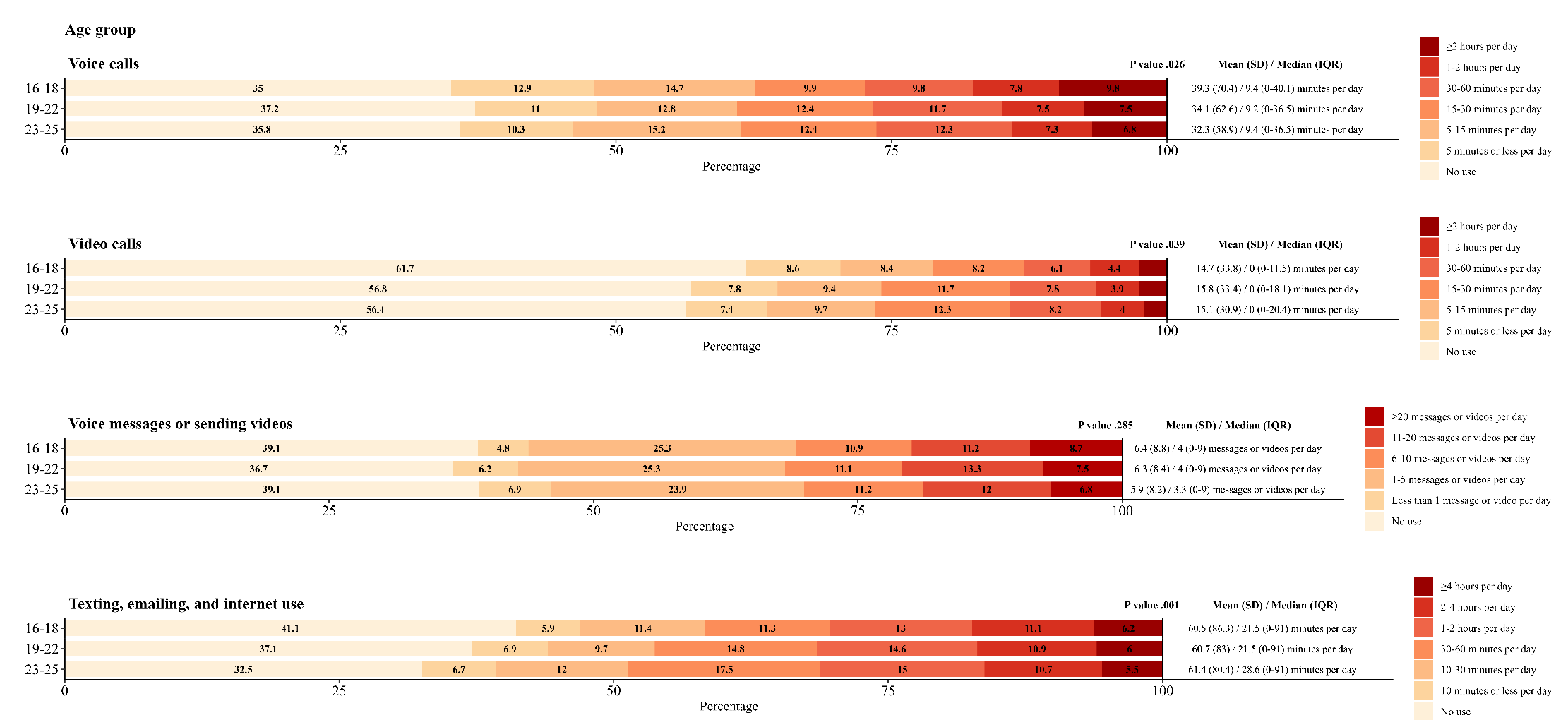
**

Figure S1. Frequencies of smartphone activity duration per day by age group and activity in young adults.

**
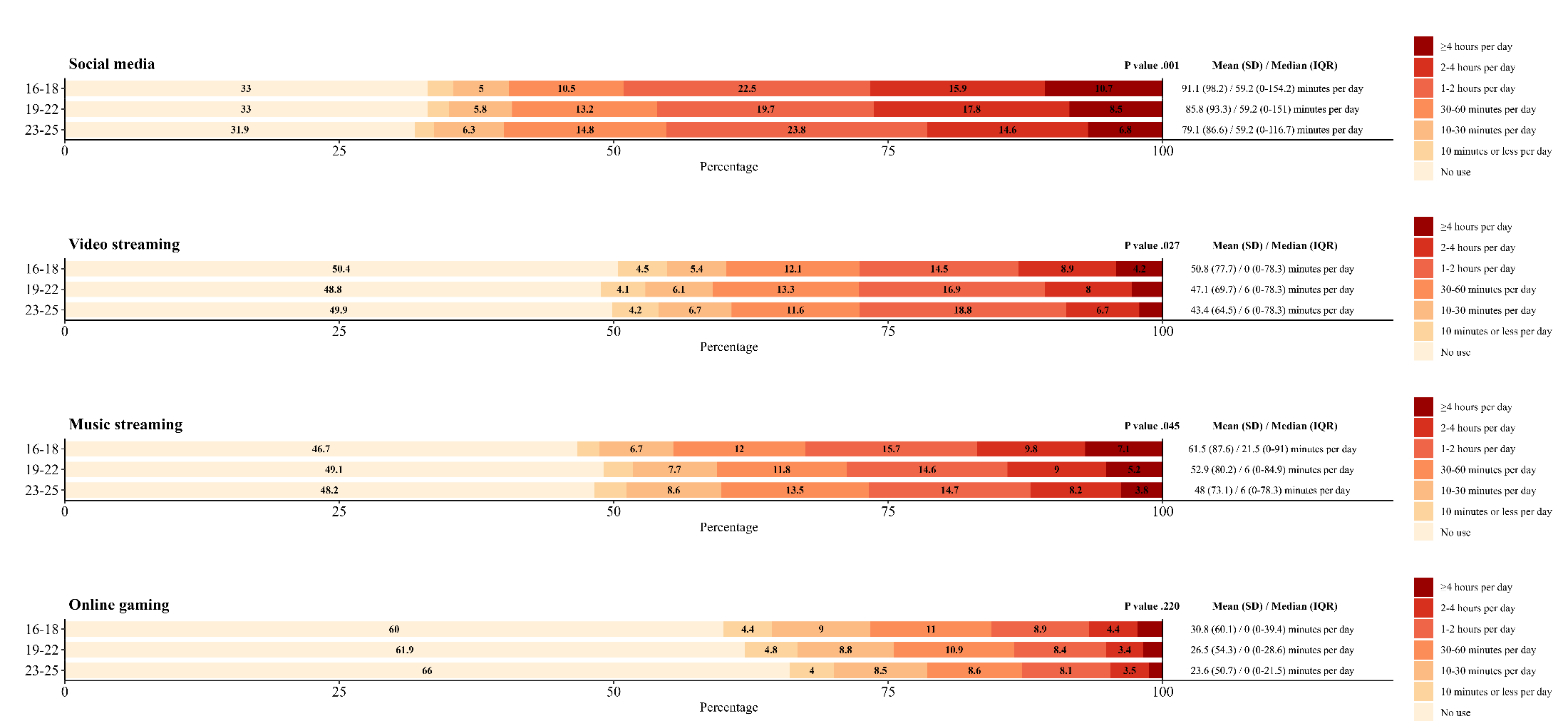
**

Figure S1. (Continuation).


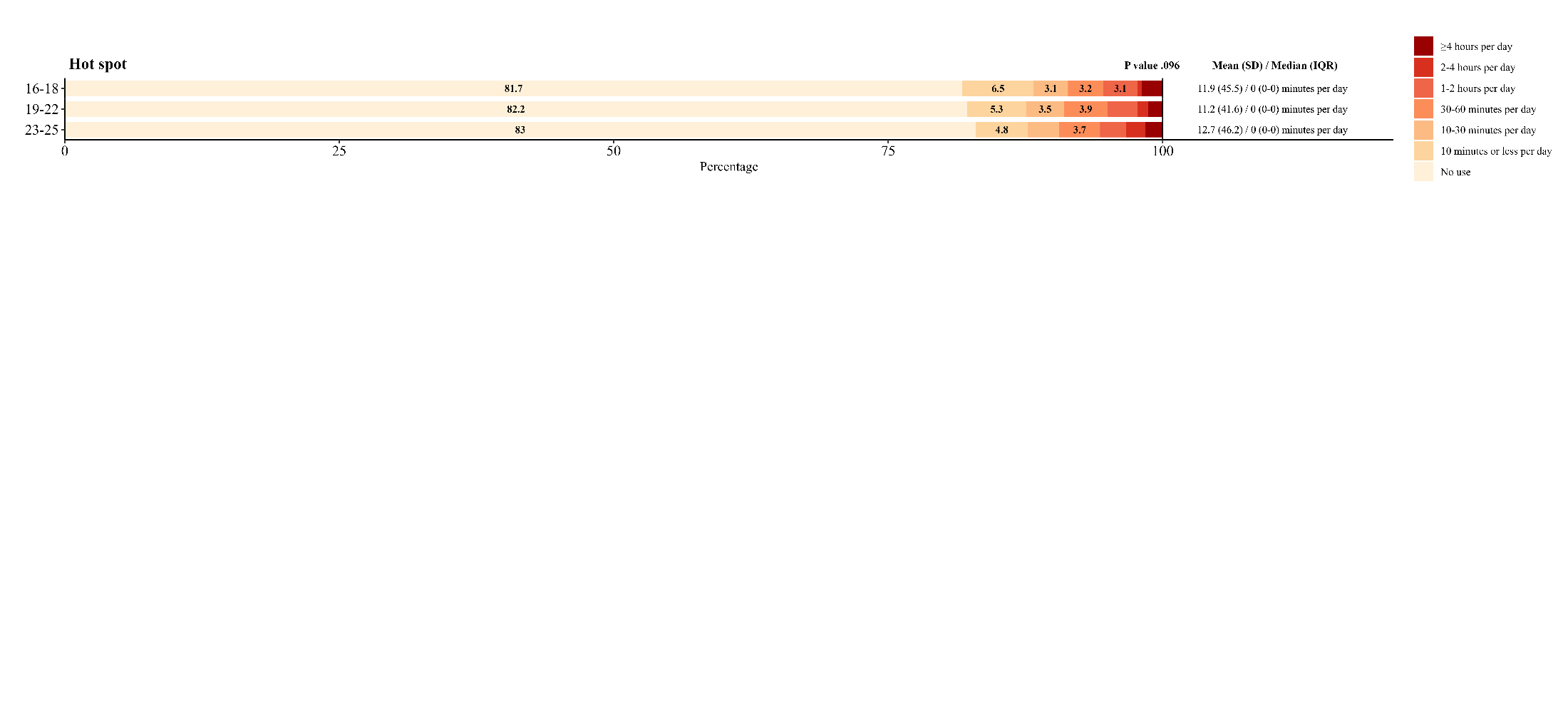


Figure S1. (Continuation).


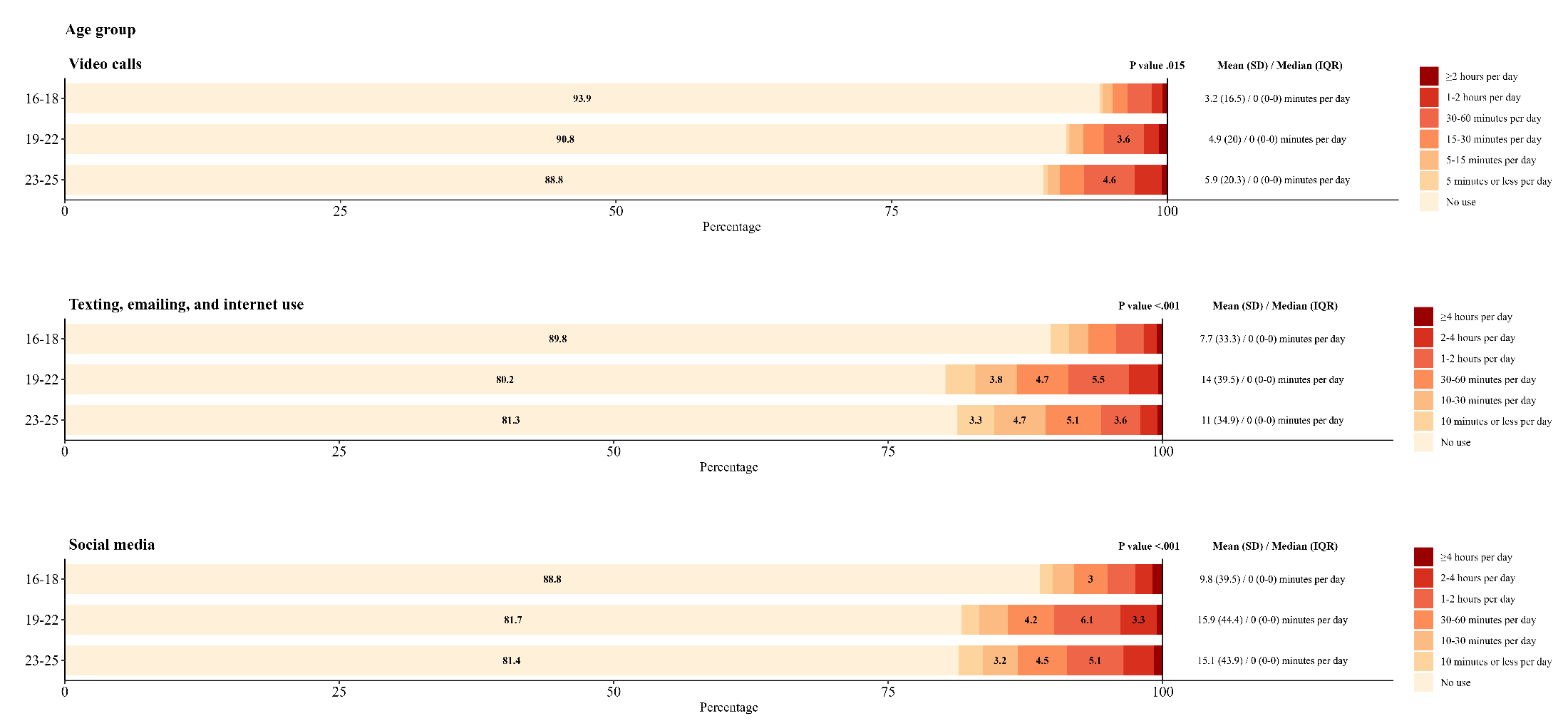


**Figure S2. Frequencies of tablet activity duration per day by age group and activity in young adults.**

**
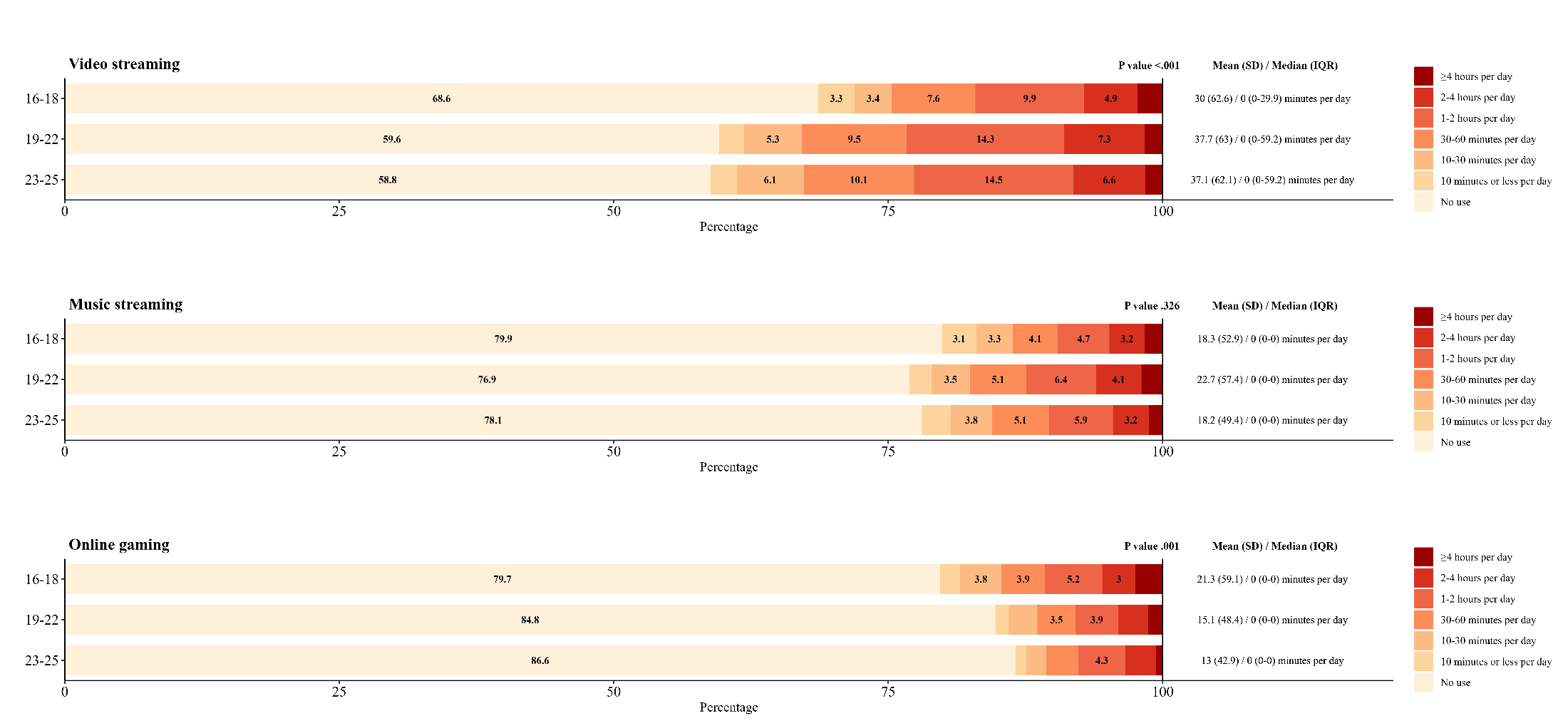
**

**Figure S2. (Continuation).**


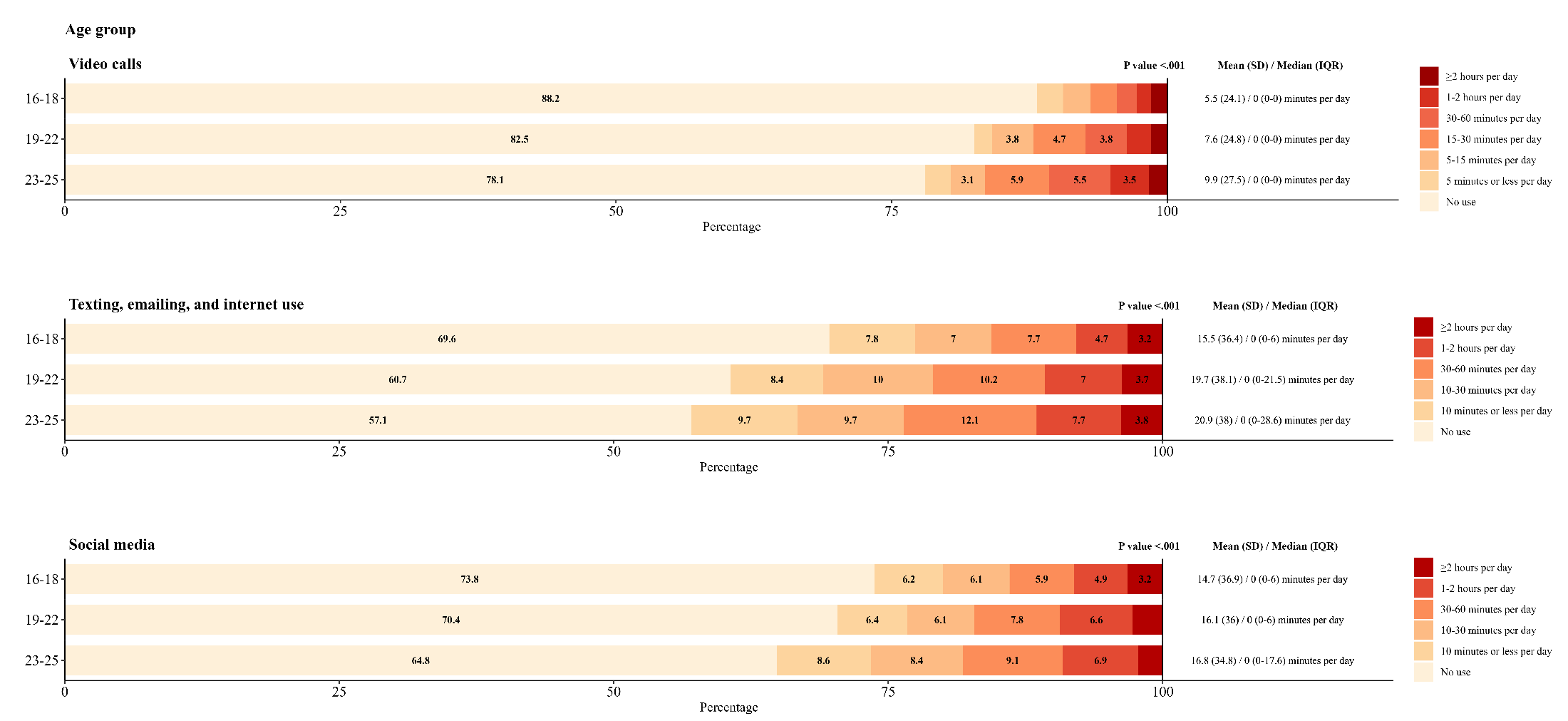


Figure S3. Frequencies of laptop activity duration per day by age group and activity in young adults.

**
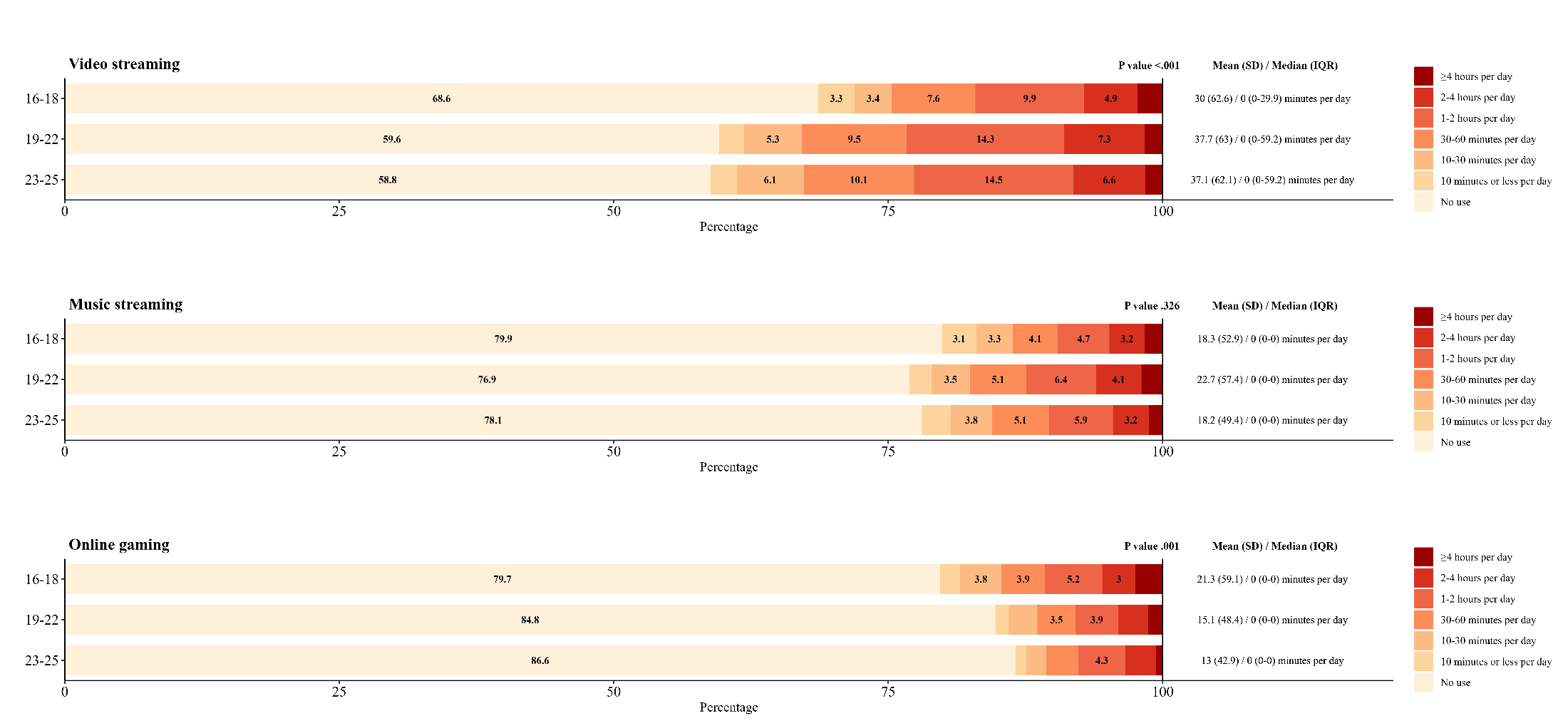
**

Figure S3. (Continuation).

**
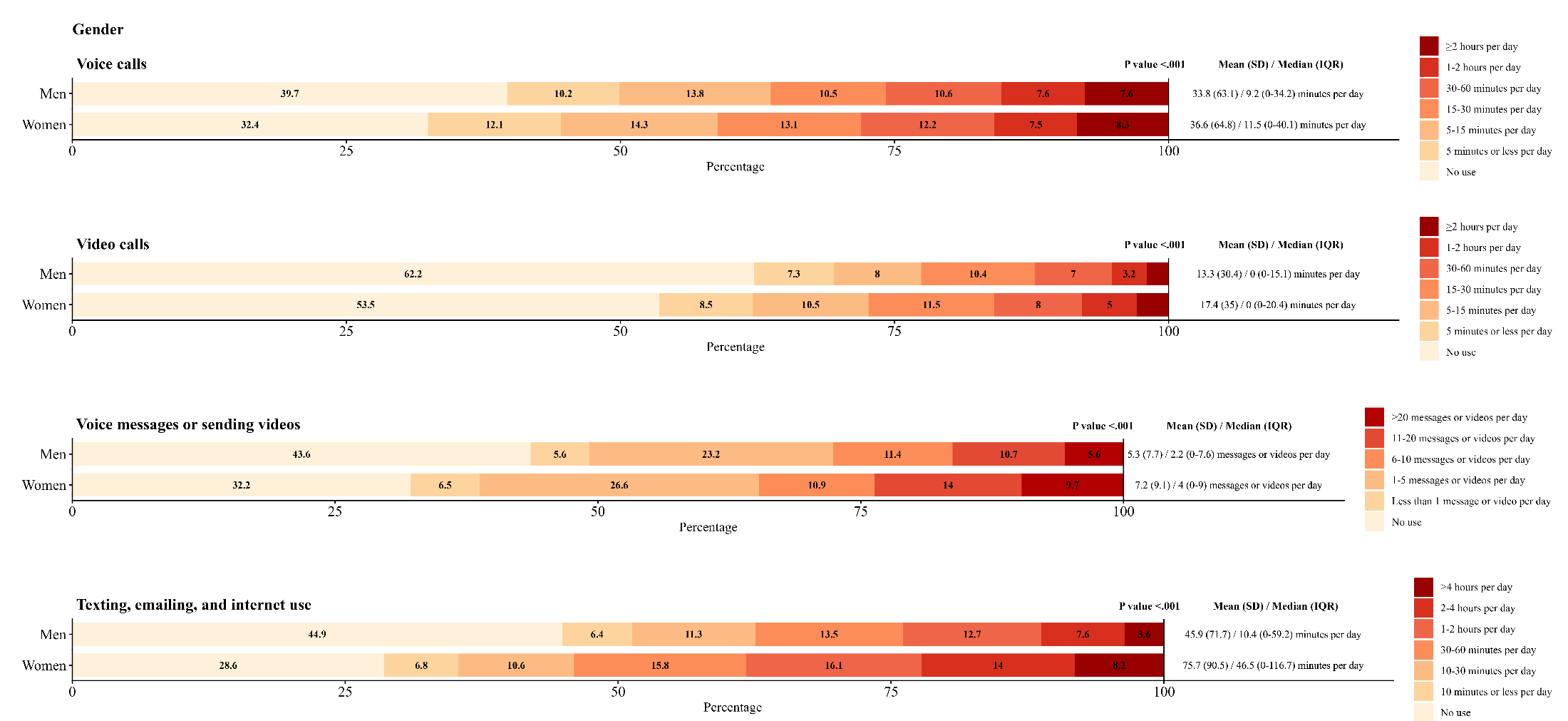
**

Figure S4. Frequencies of smartphone activity duration per day by gender and activity in young adults.

**
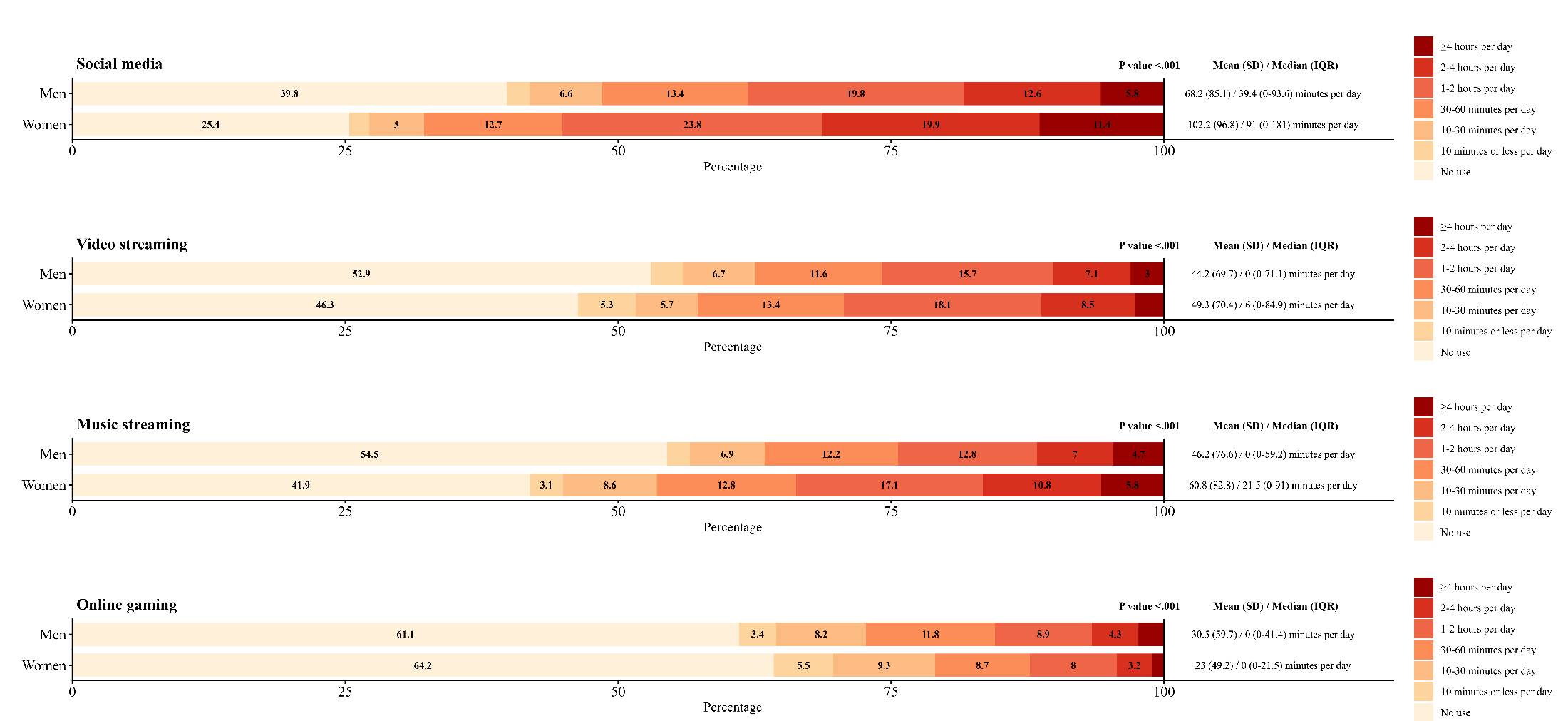
**

Figure S4. (Continuation).


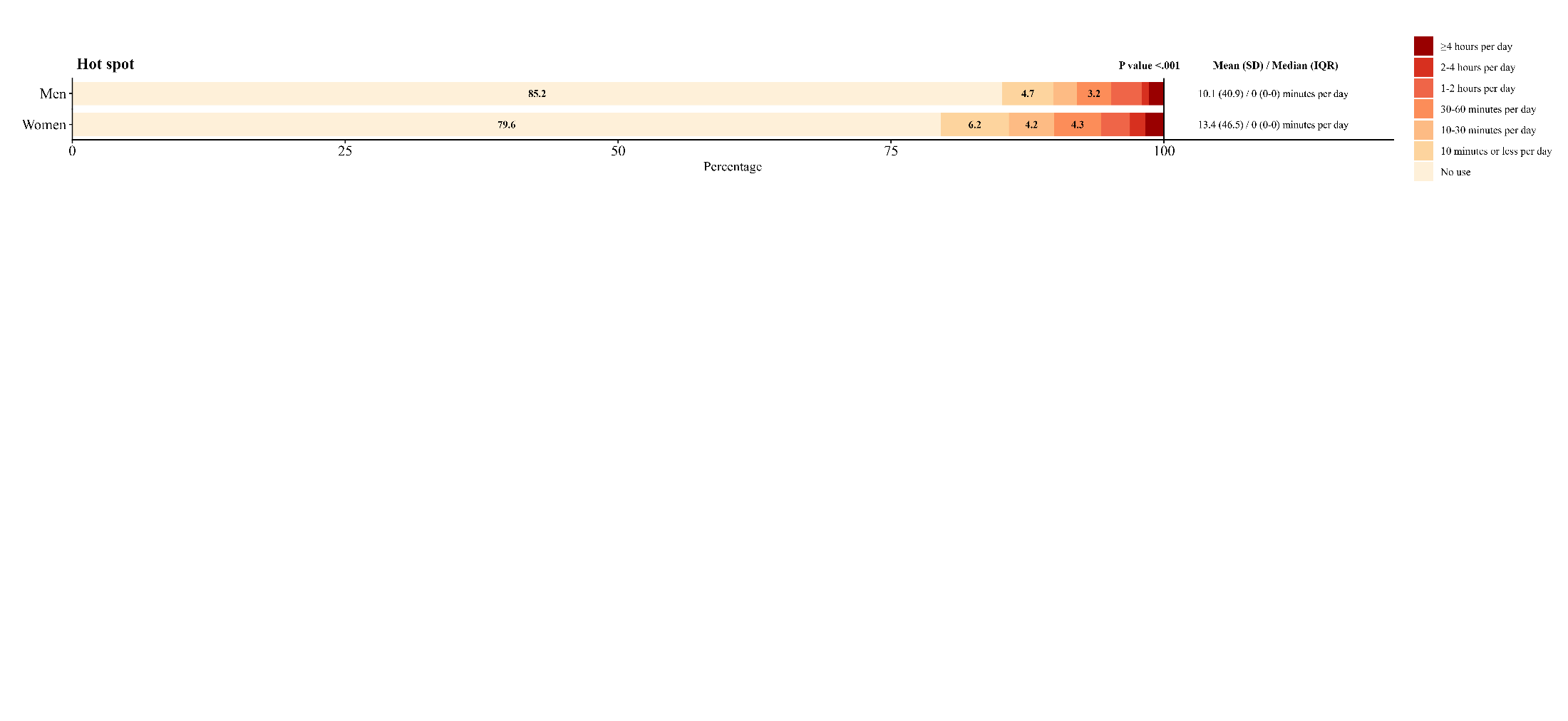


Figure S4. (Continuation).


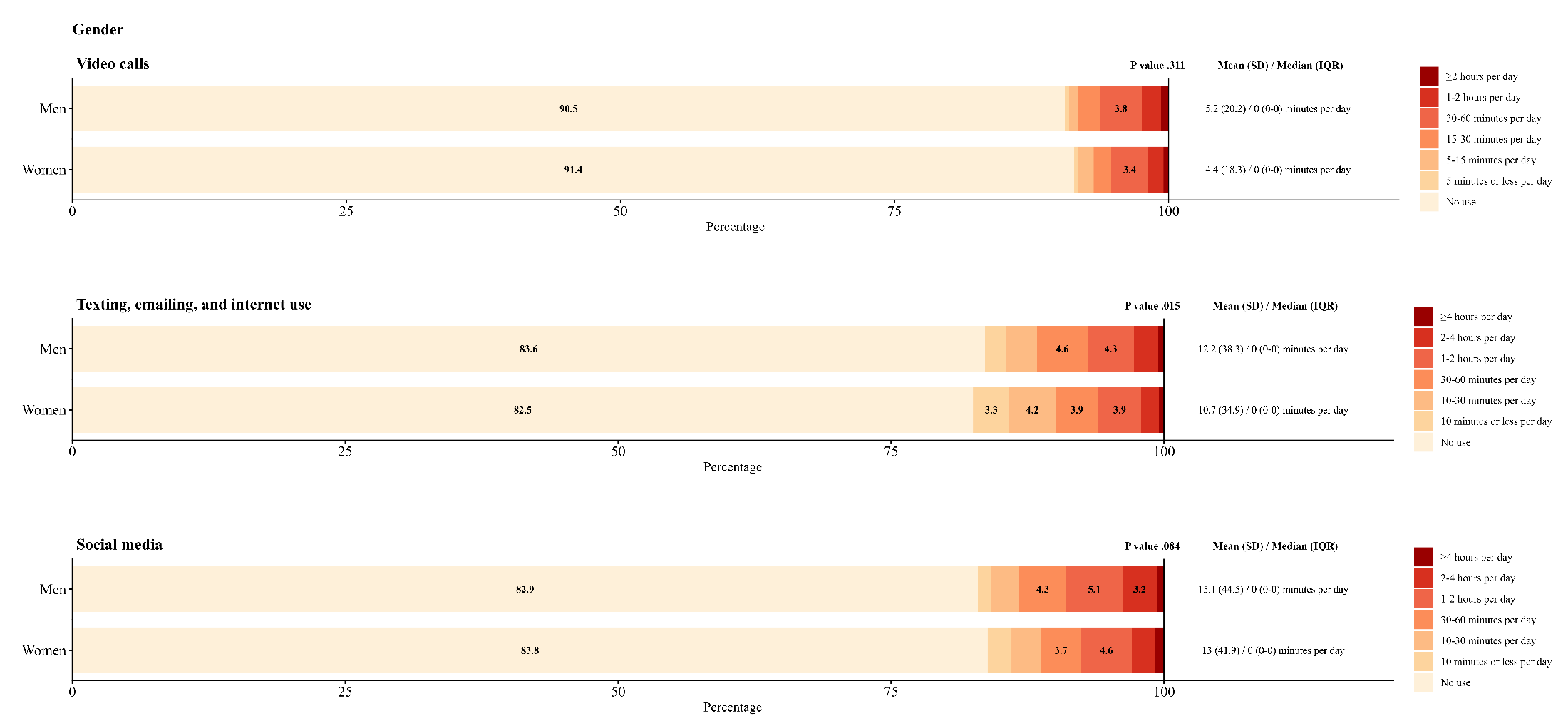


Figure S5. Frequencies of tablet activity duration per day by gender and activity in young adults.

**
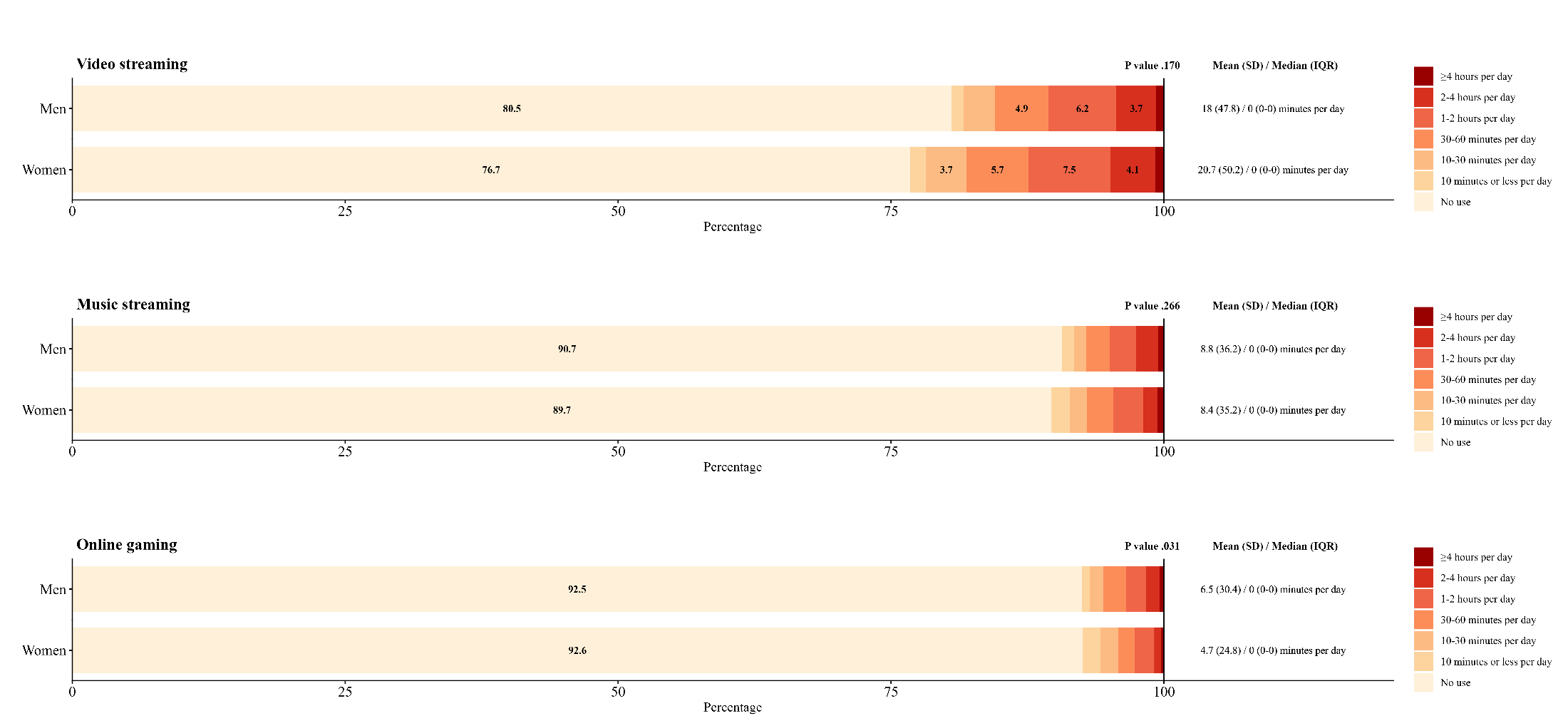
**

Figure S5. (Continuation).


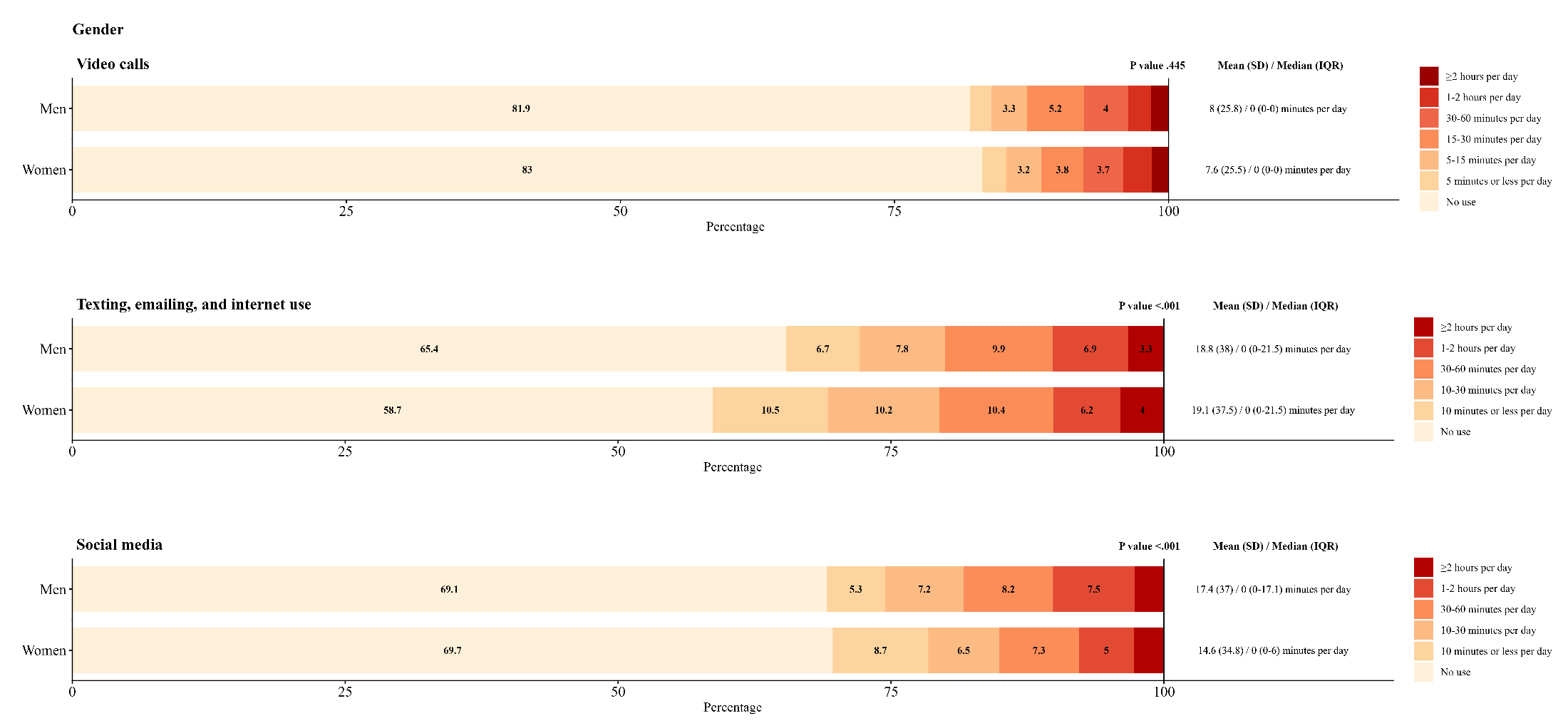


Figure S6. Frequencies of laptop activity duration per day by gender and activity in young adults.

**
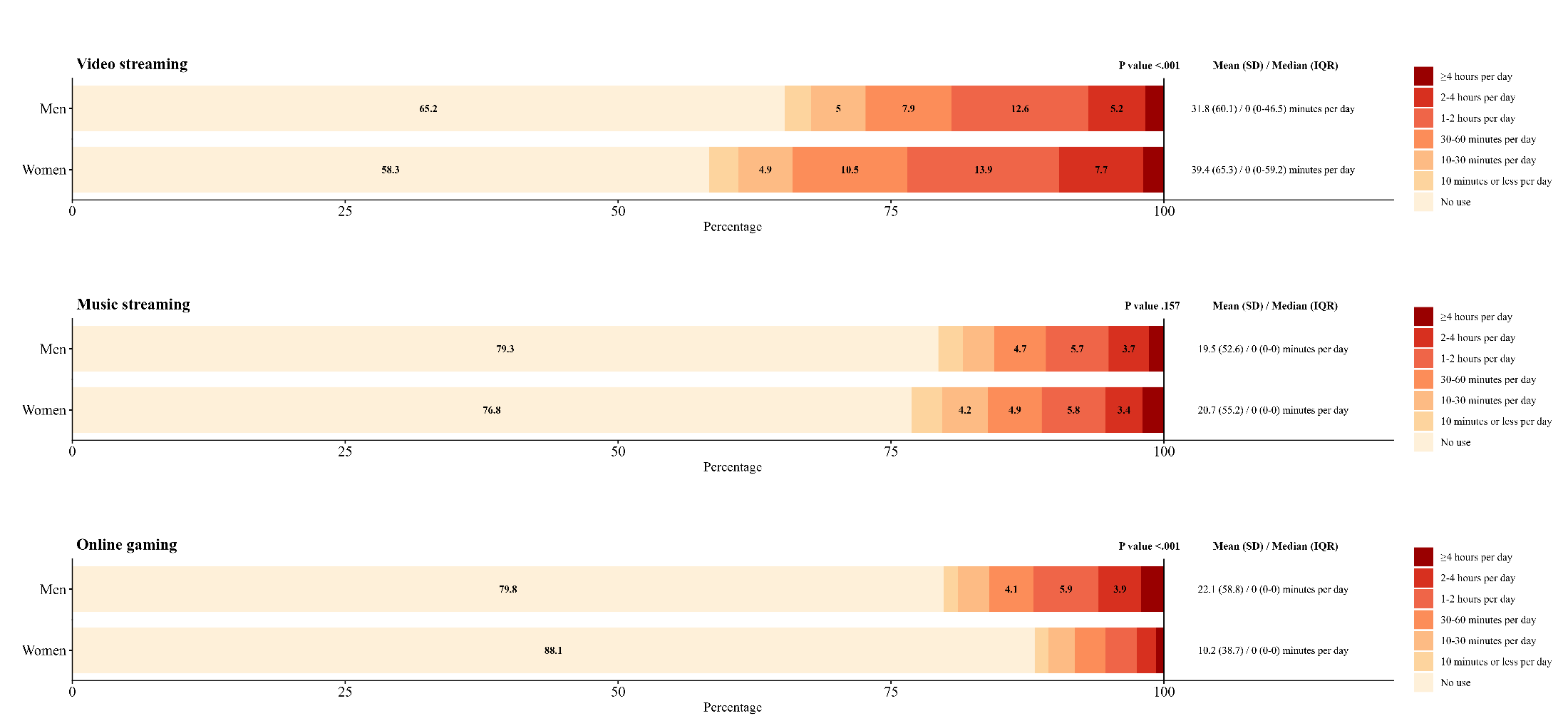
**

Figure S6. (Continuation).


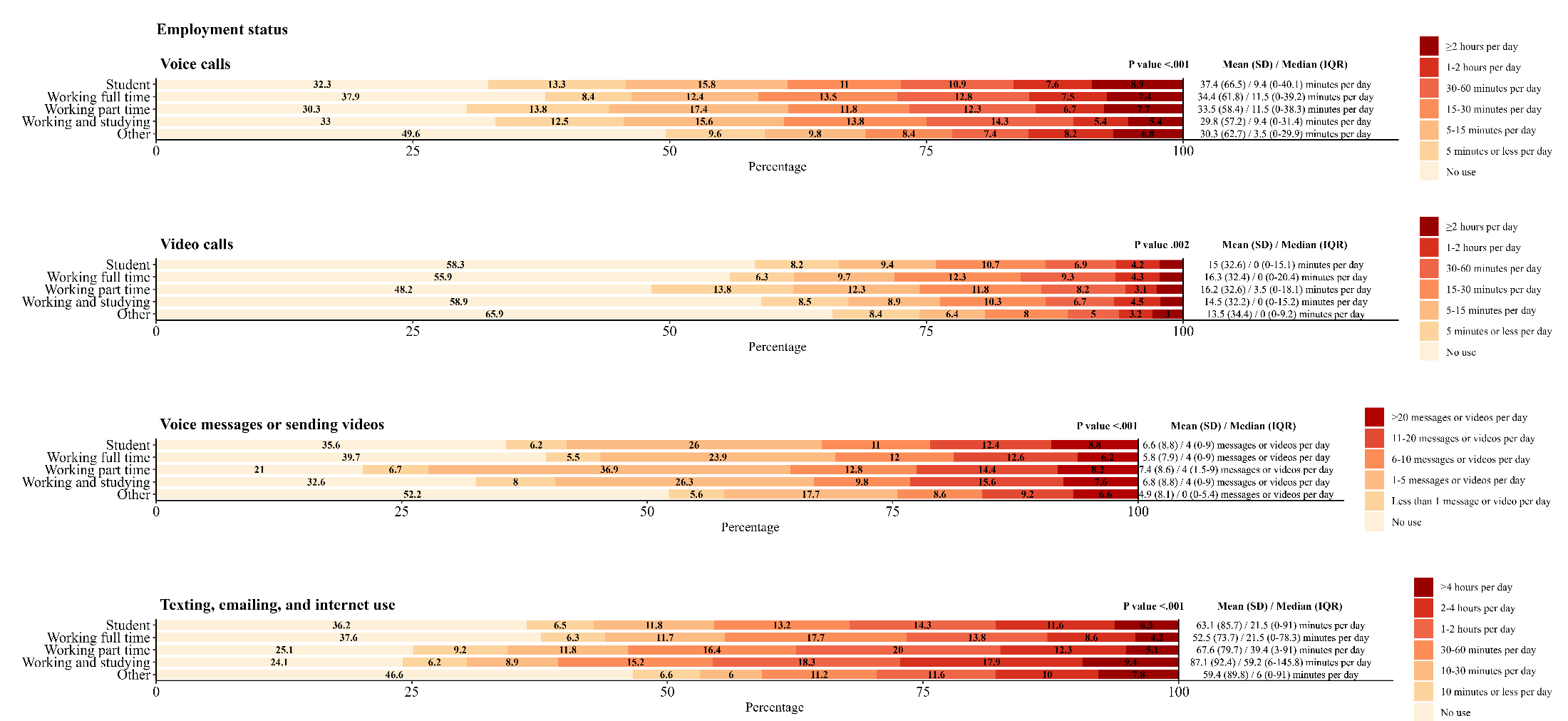


Figure S7. Frequencies of smartphone activity duration per day by employment status and activity in young adults.

**
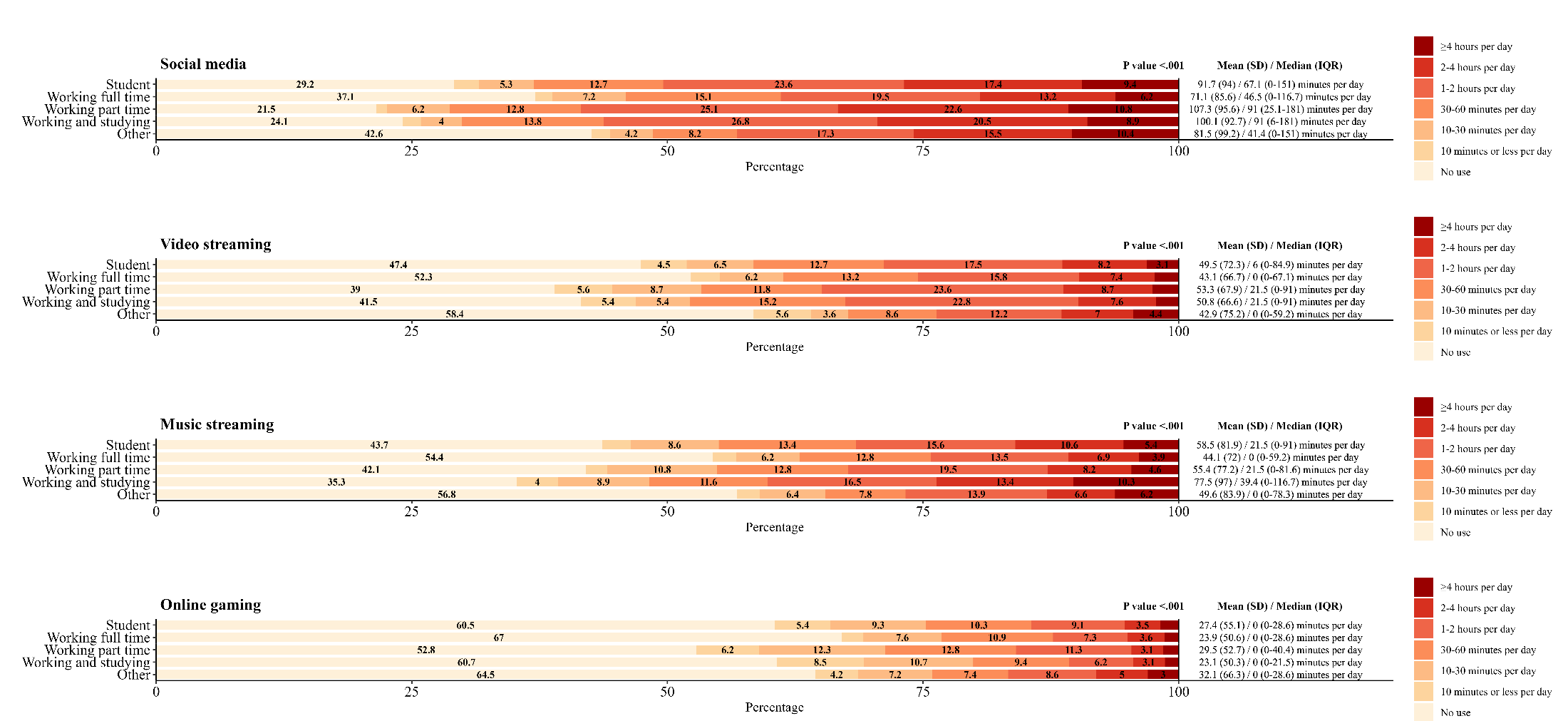
**

Figure S7. (Continuation).


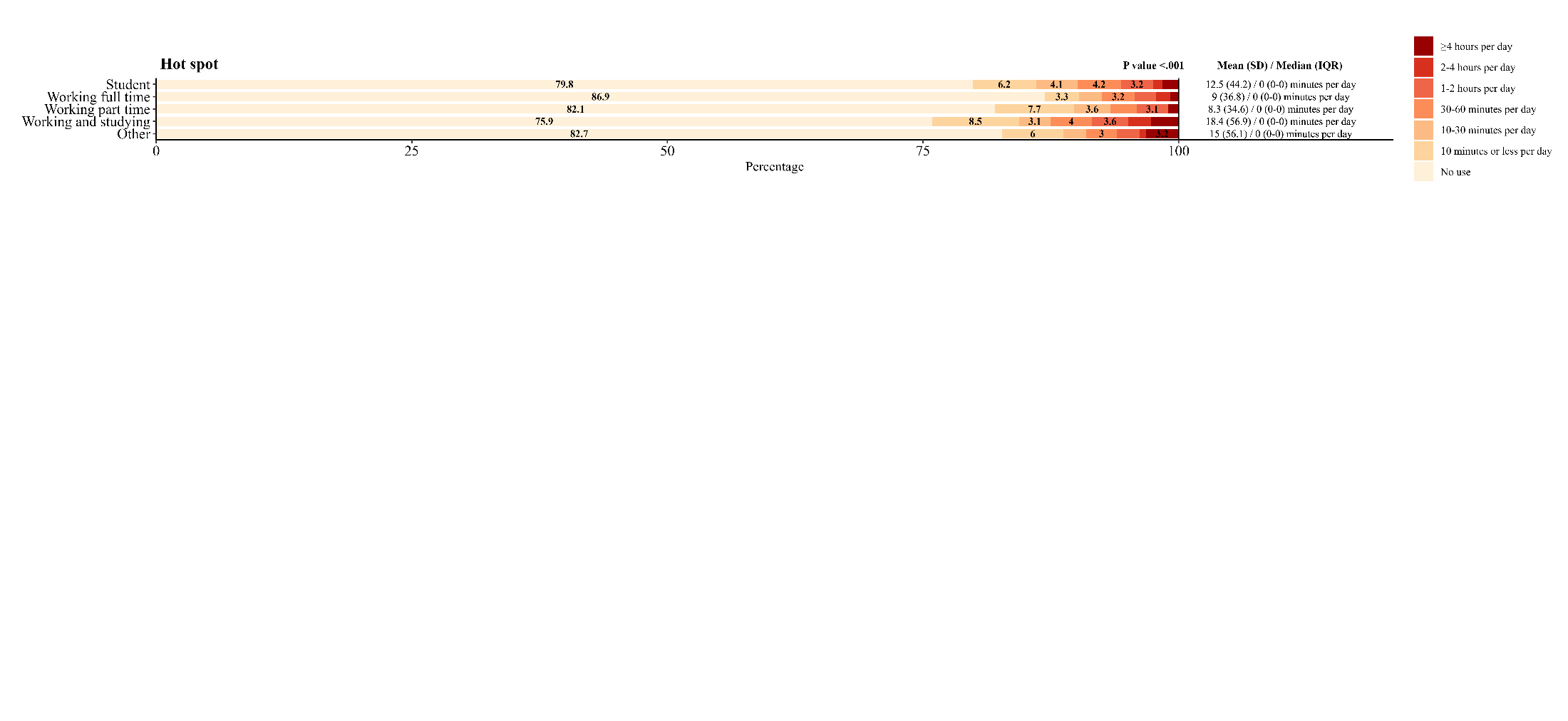


Figure S7. (Continuation).


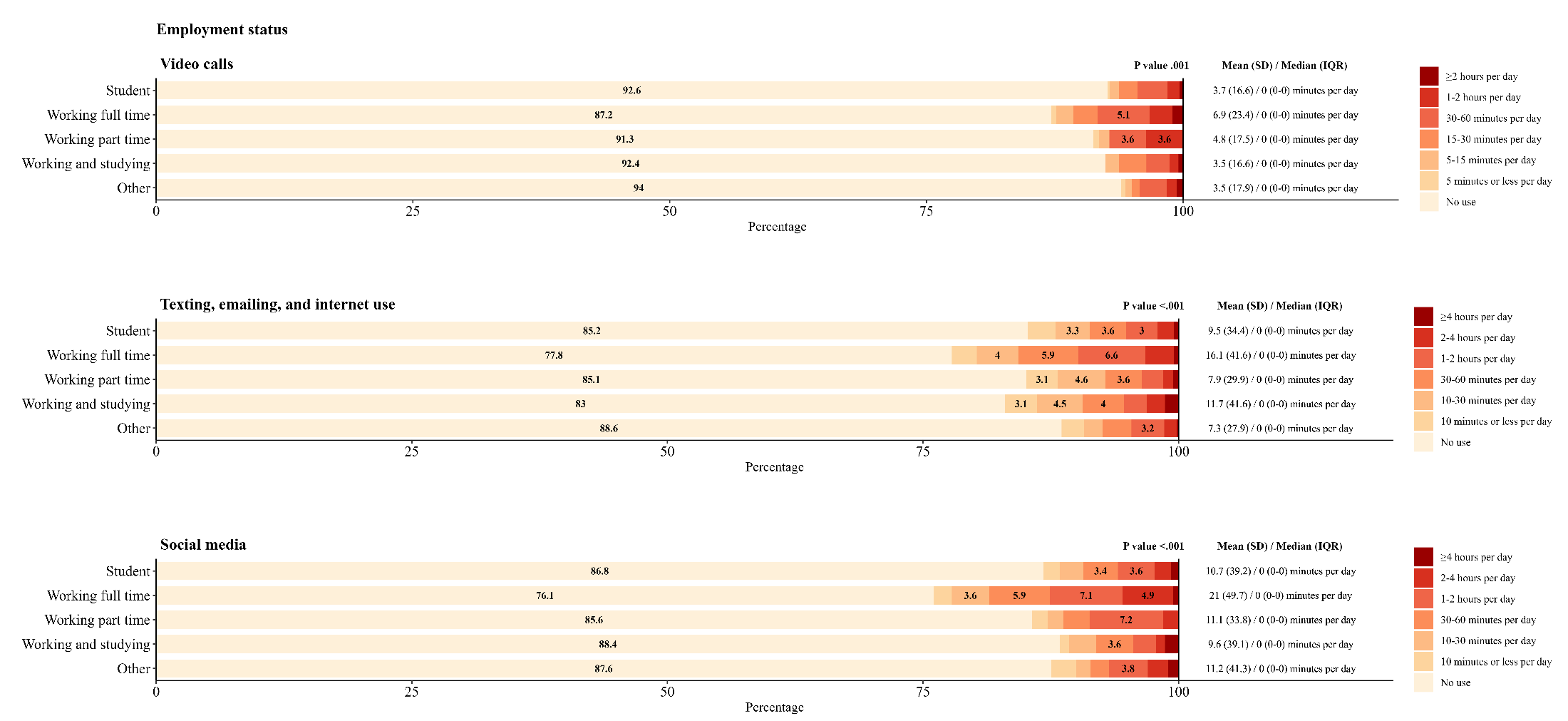


Figure S8. Frequencies of tablet activity duration per day by employment status and activity in young adults.

**
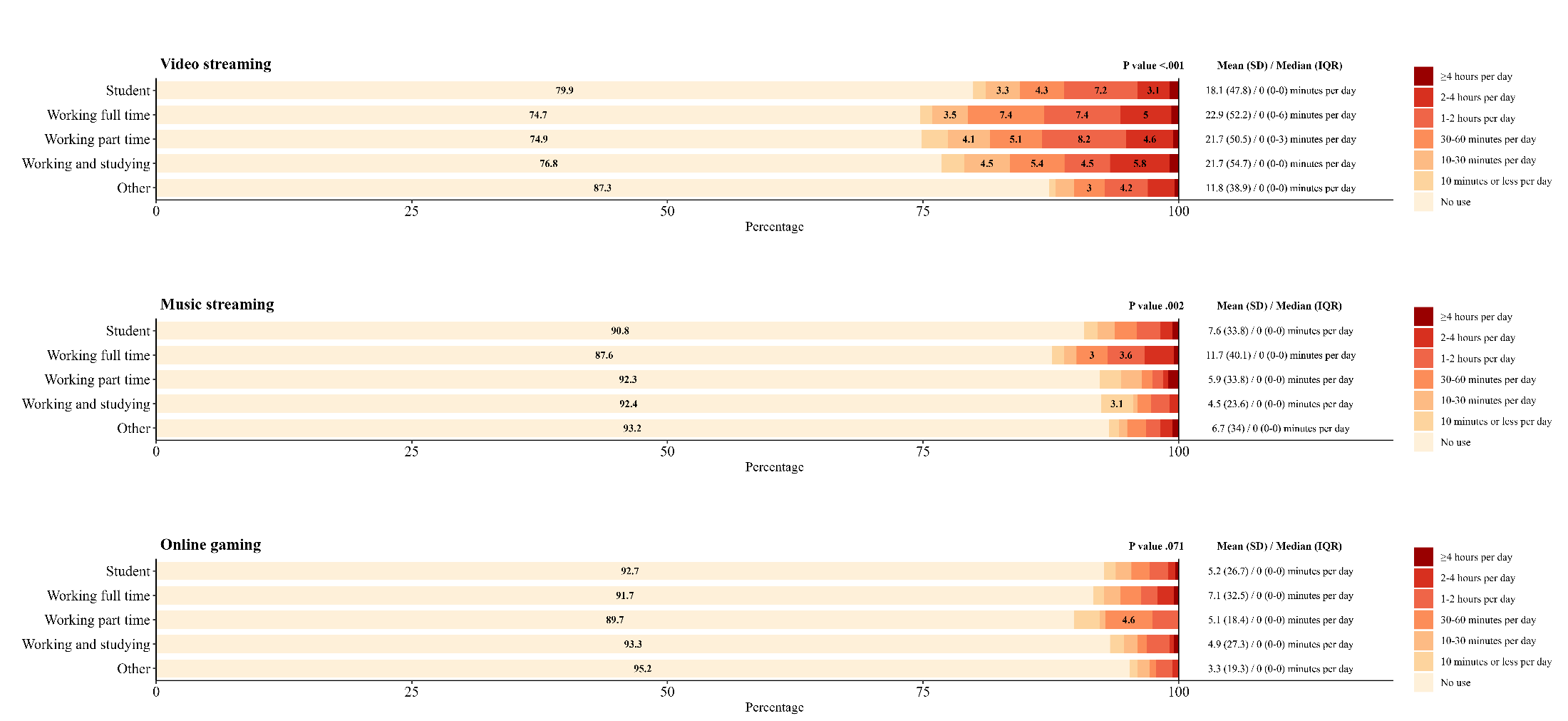
**

**Figure S8. (Continuation).**


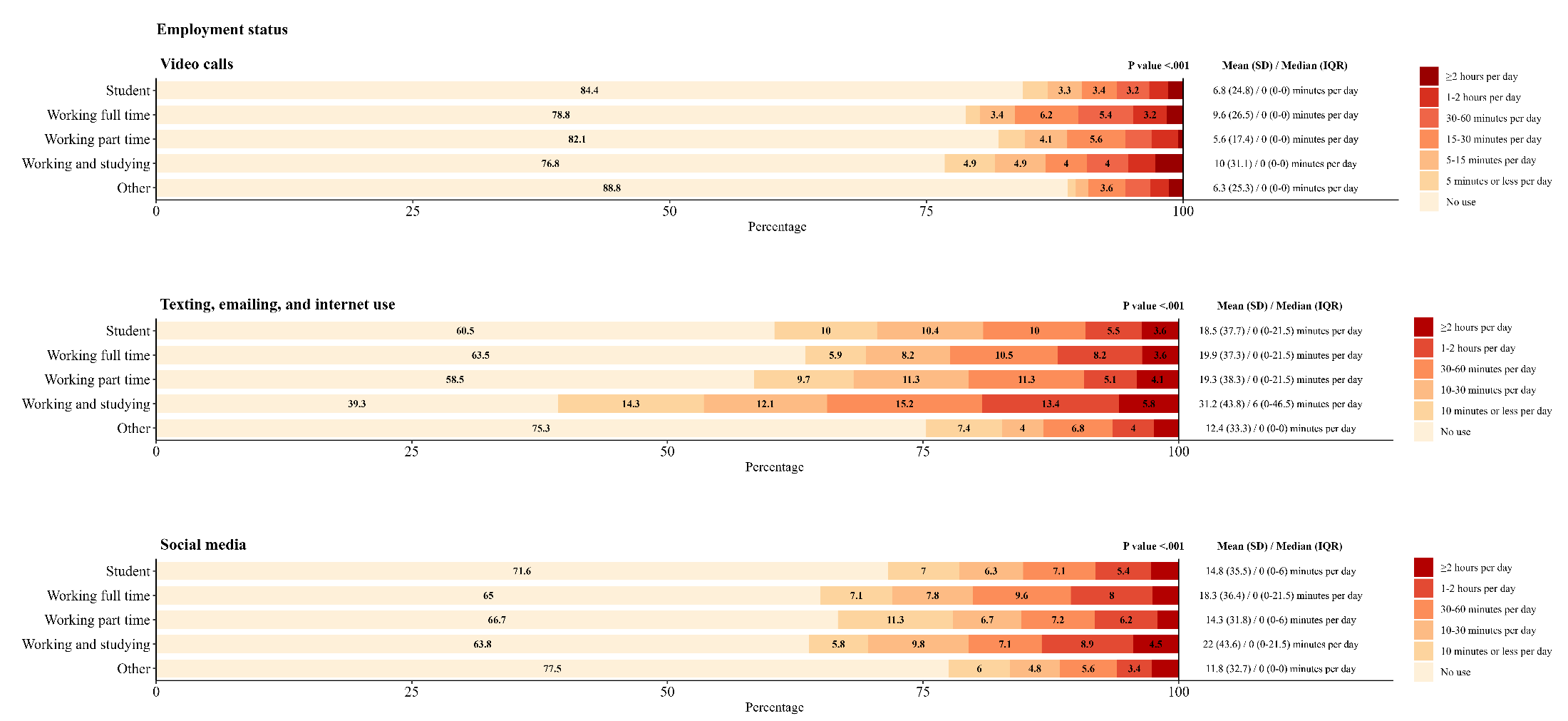


Figure S9. Frequencies of laptop activity duration per day by employment status and activity in young adults.

**
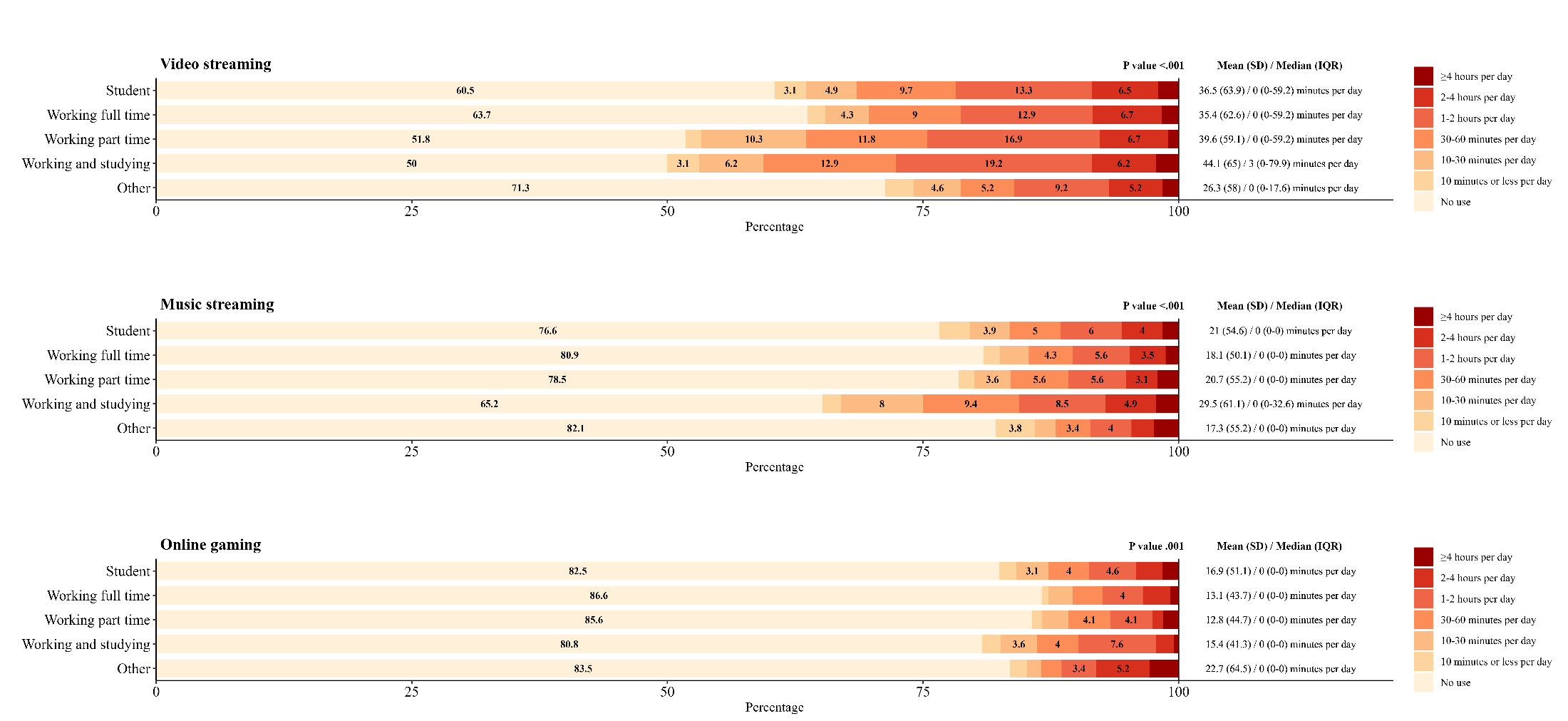
**

**Figure S9. (Continuation).**


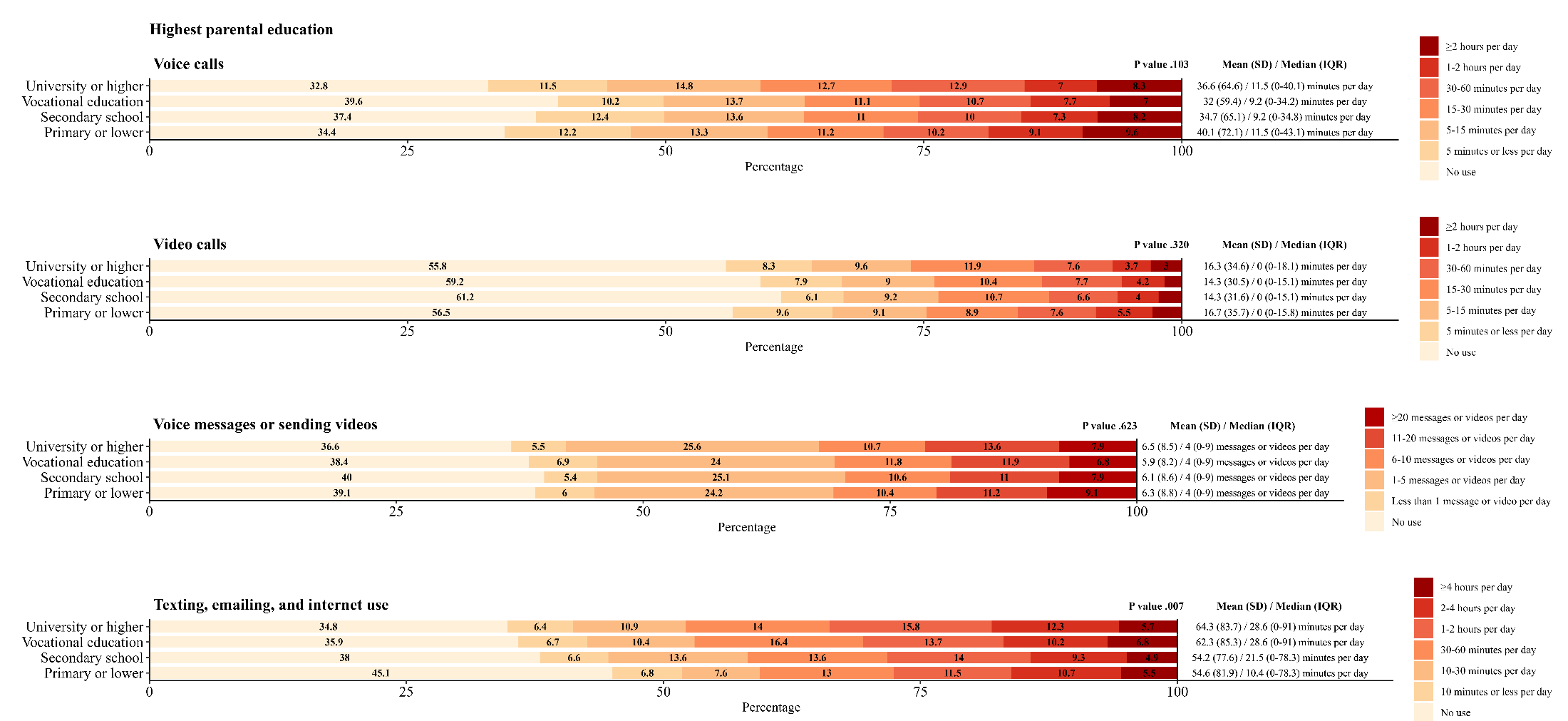


Figure S10. Frequencies of smartphone activity duration per day by highest parental education and activity in young adults.

**
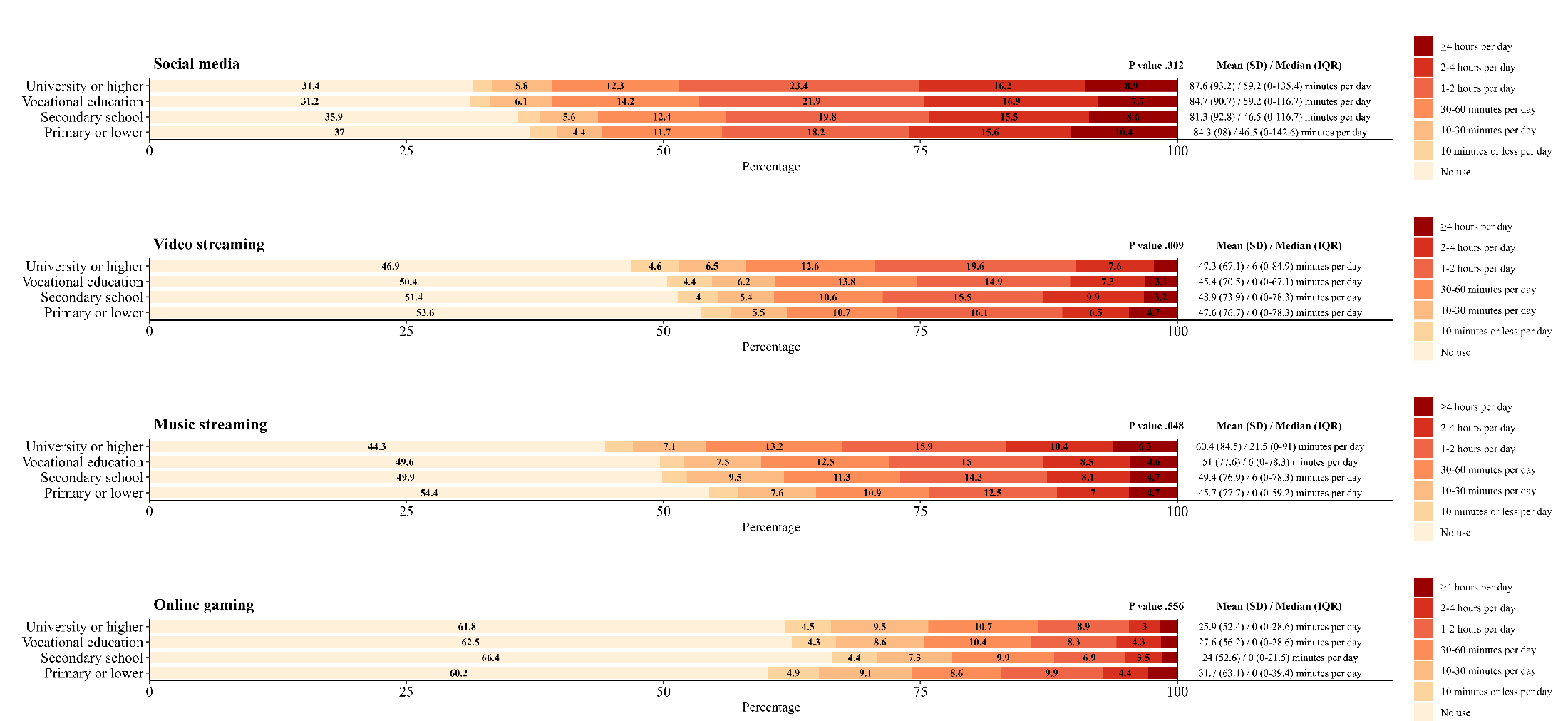
**

Figure S10. (Continuation).


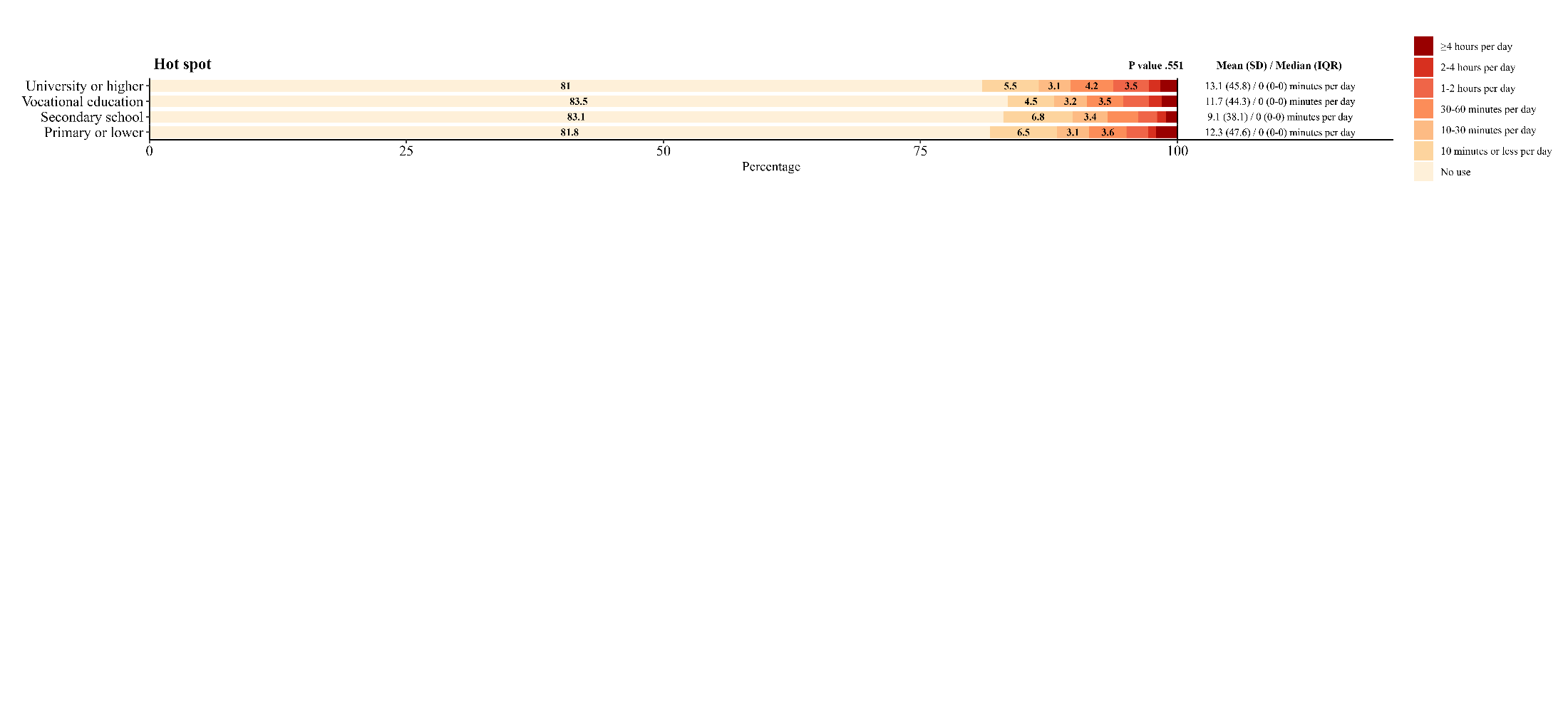


Figure S10. (Continuation).


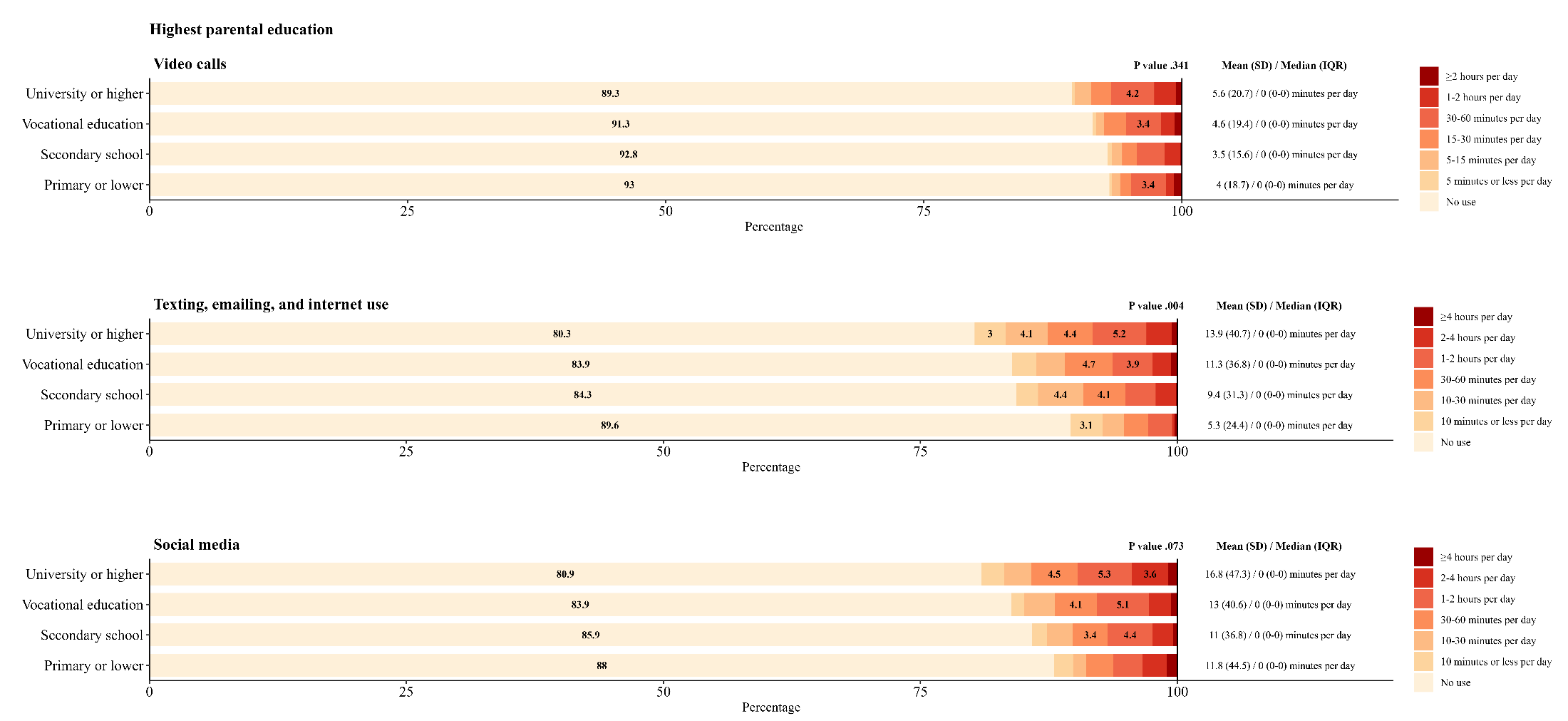


Figure S11. Frequencies of tablet activity duration per day by highest parental education and activity in young adults.

**
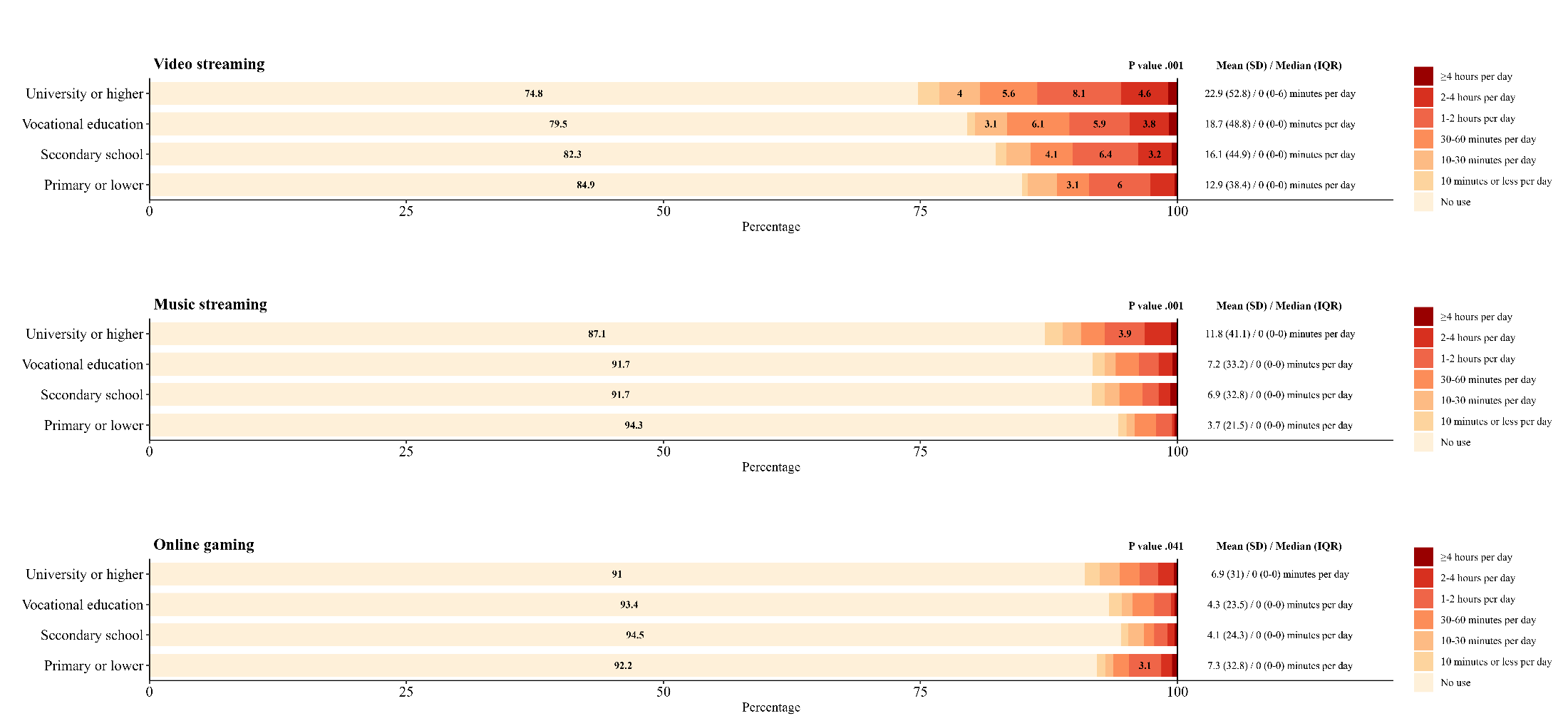
**

Figure S11. (Continuation).


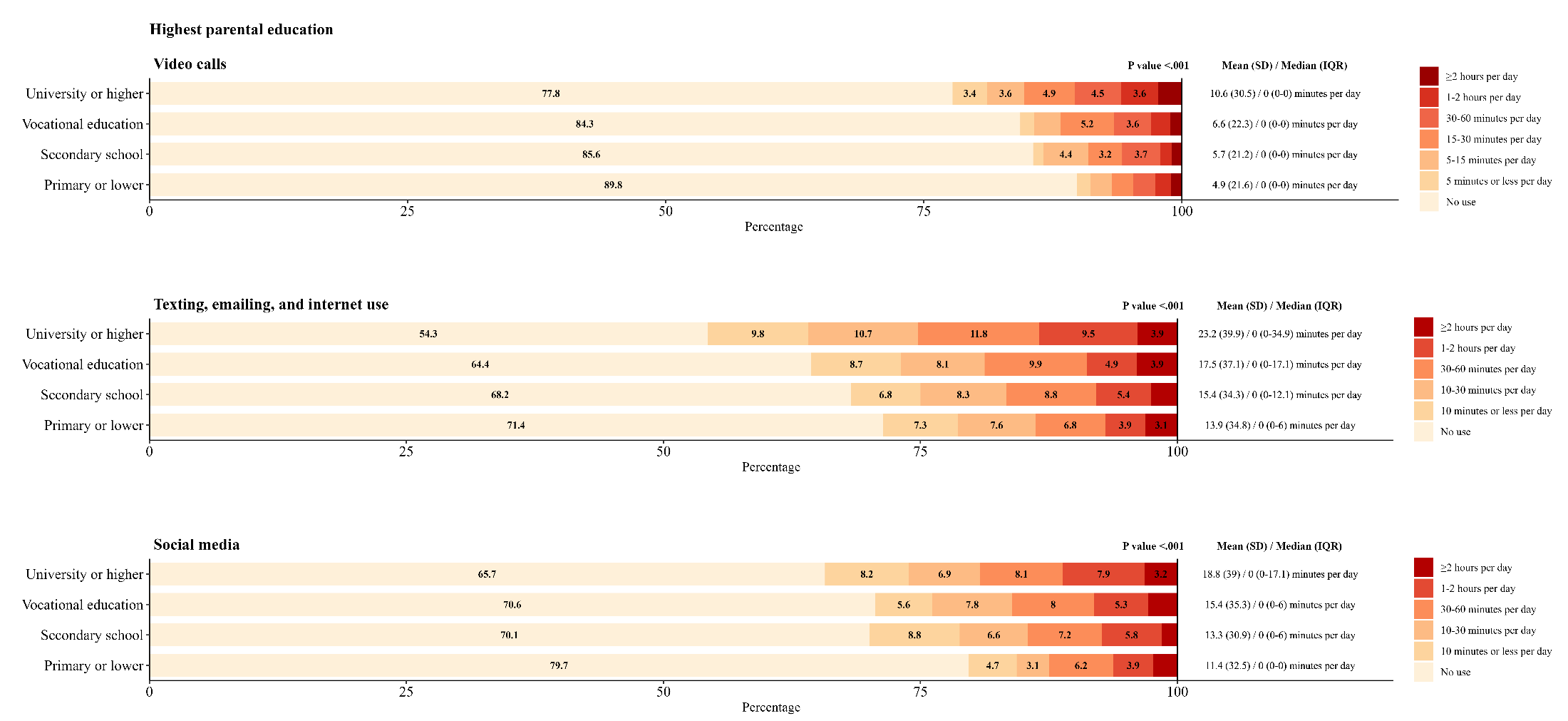


Figure S12. Frequencies of laptop activity duration per day by highest parental education and activity in young adults.

**
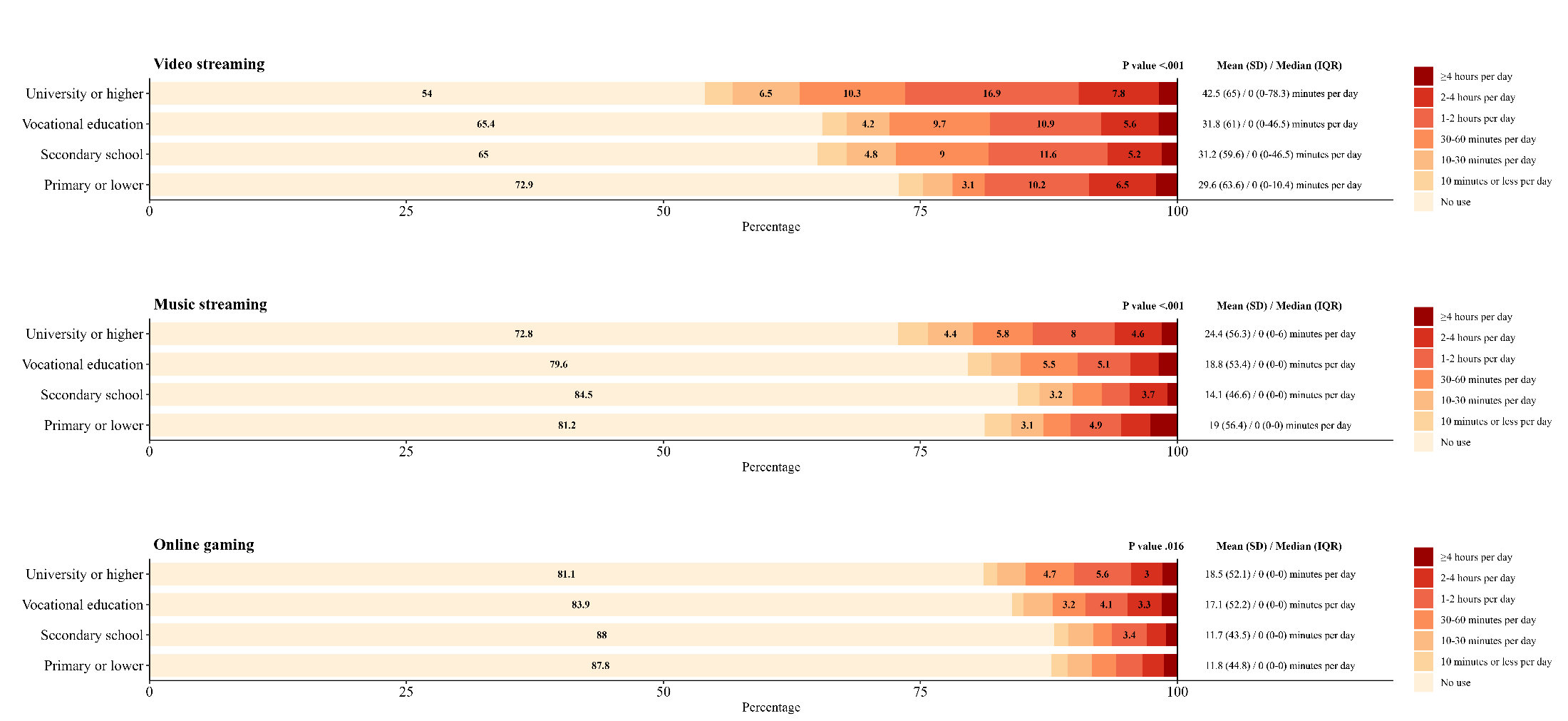
**

Figure S12. (Continuation).


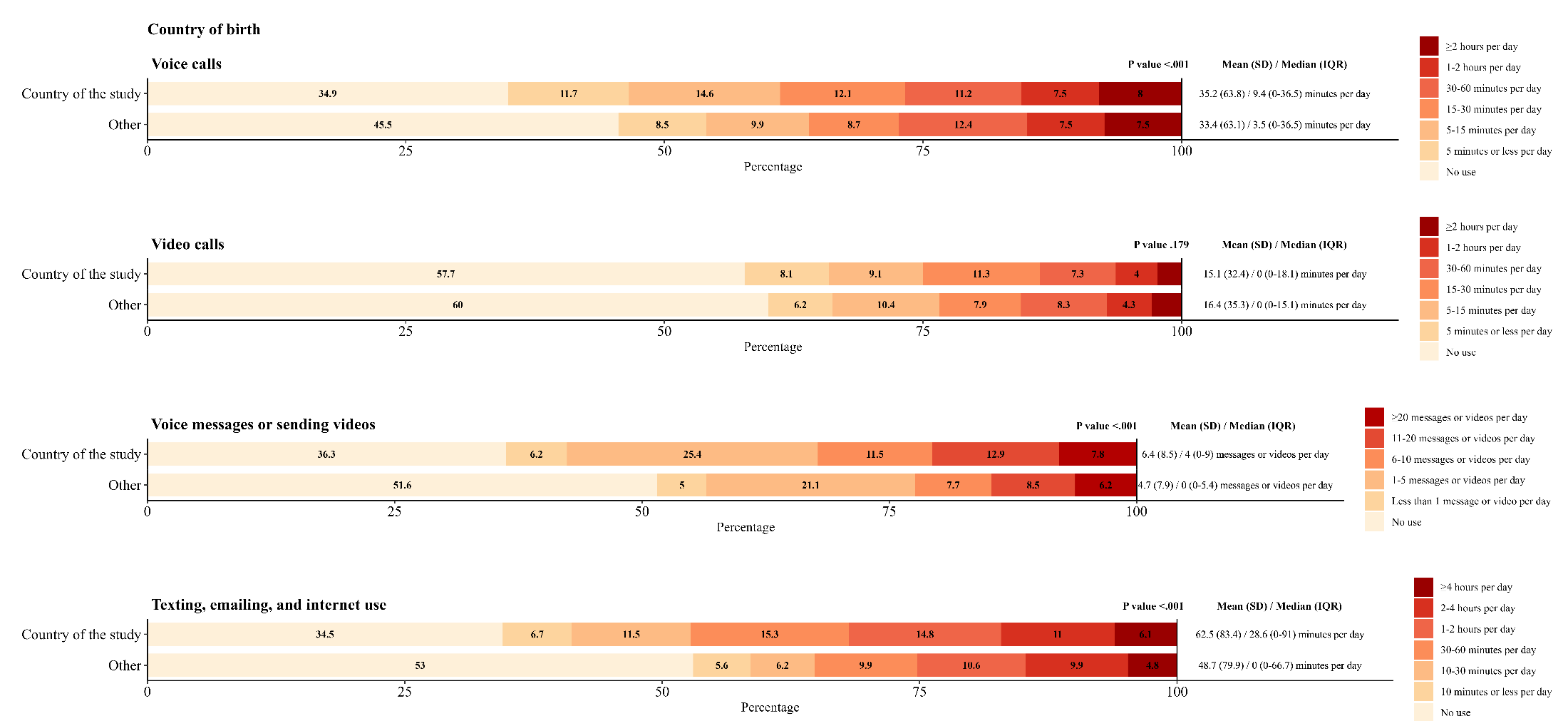


Figure S13. Frequencies of smartphone activity duration per day by country of birth and activity in young adults.

**
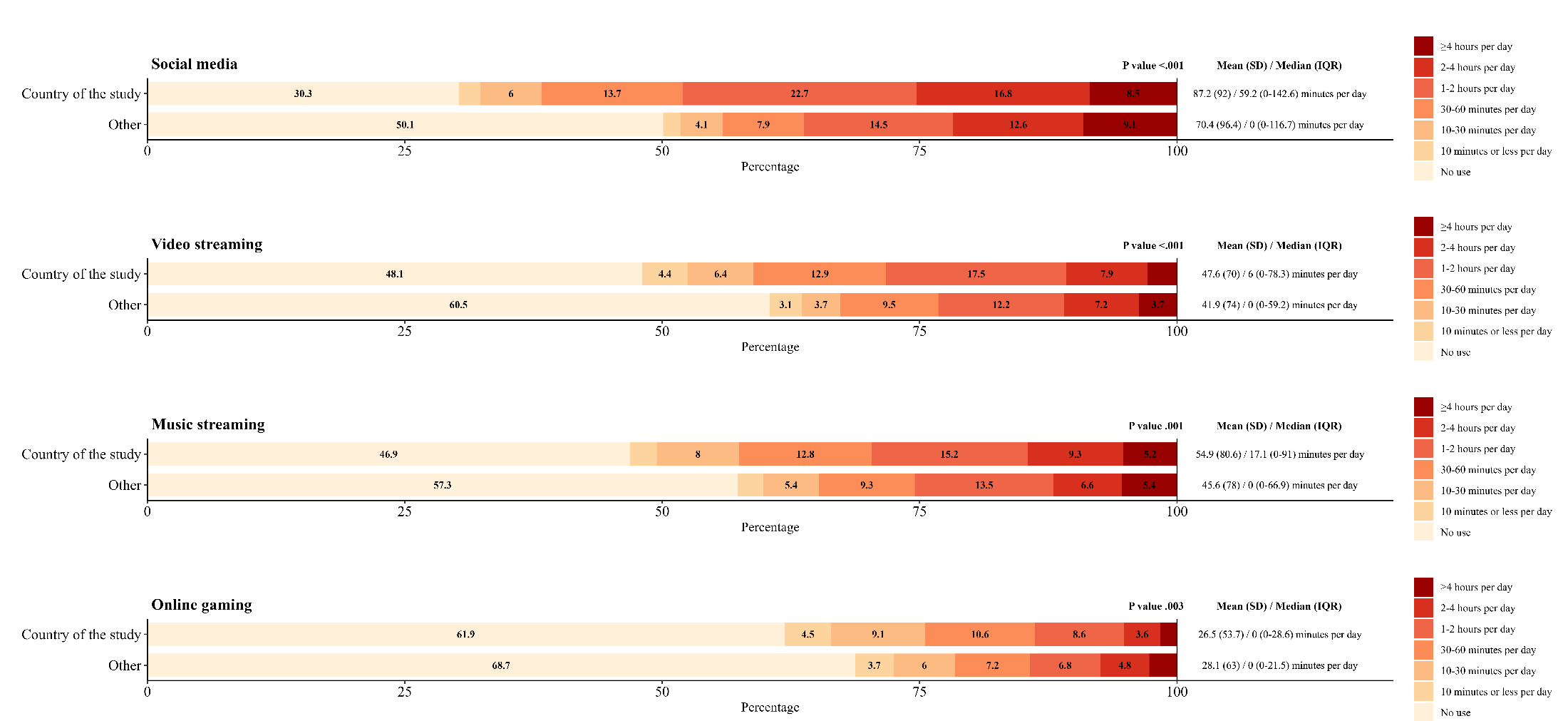
**

Figure S13. (Continuation).


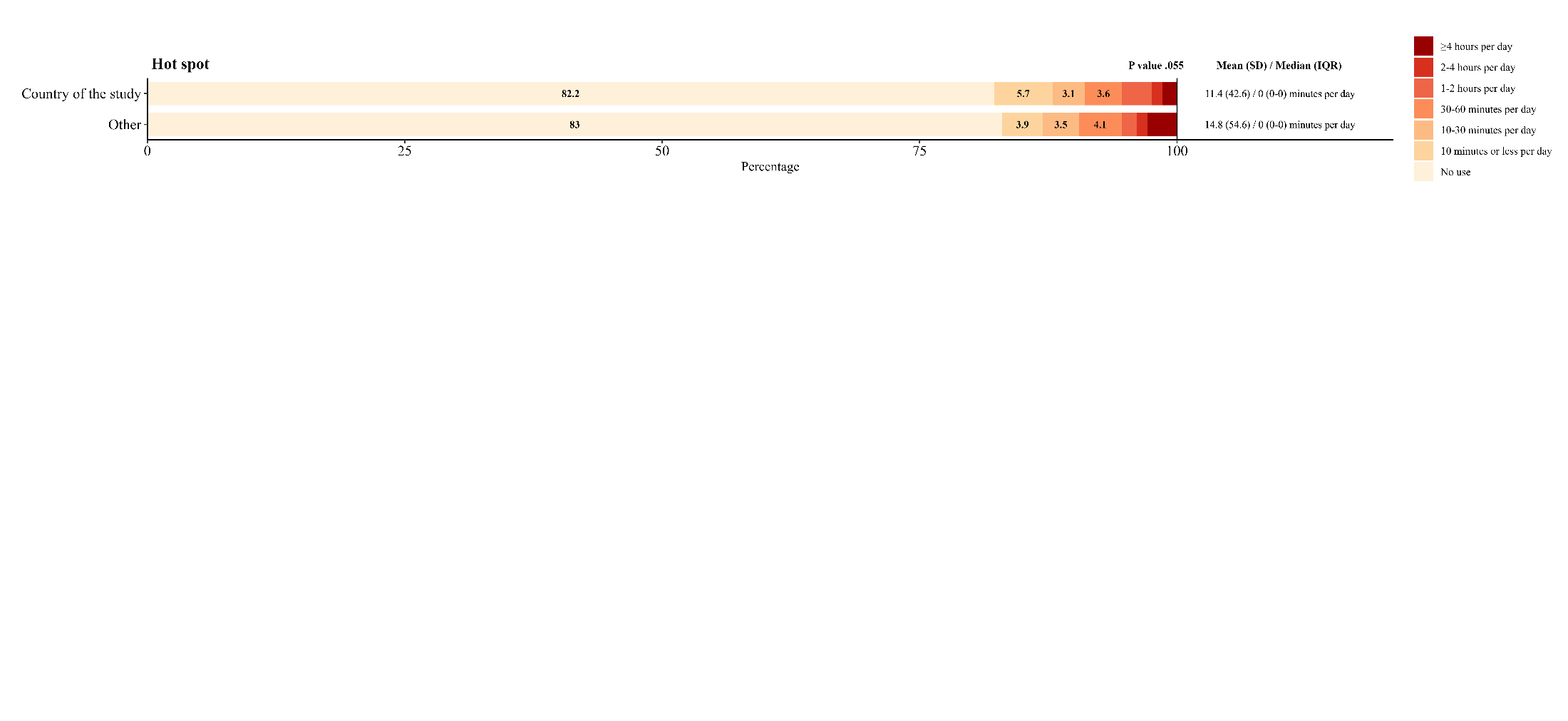


Figure S13. (Continuation).


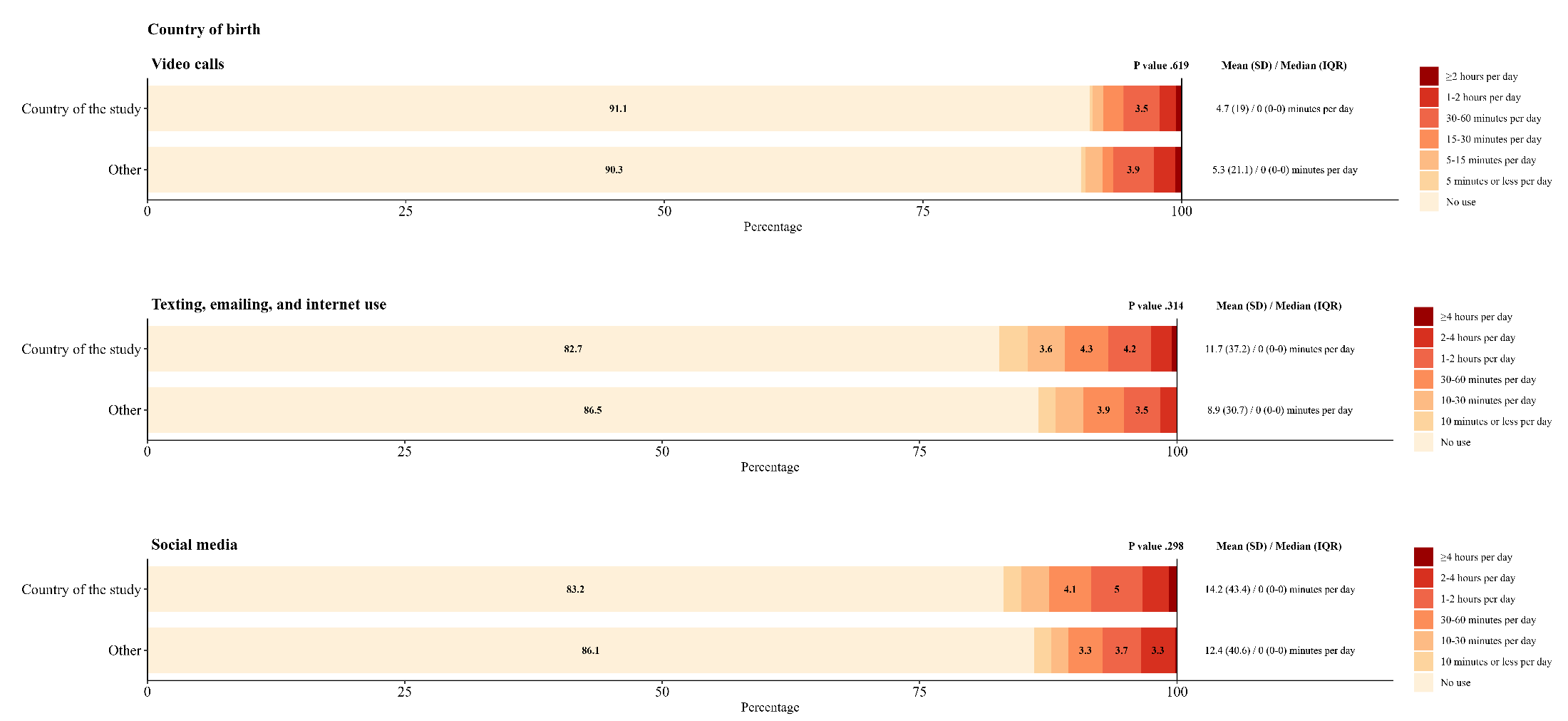


Figure S14. Frequencies of tablet activity duration per day by country of birth and activity in young adults.

**
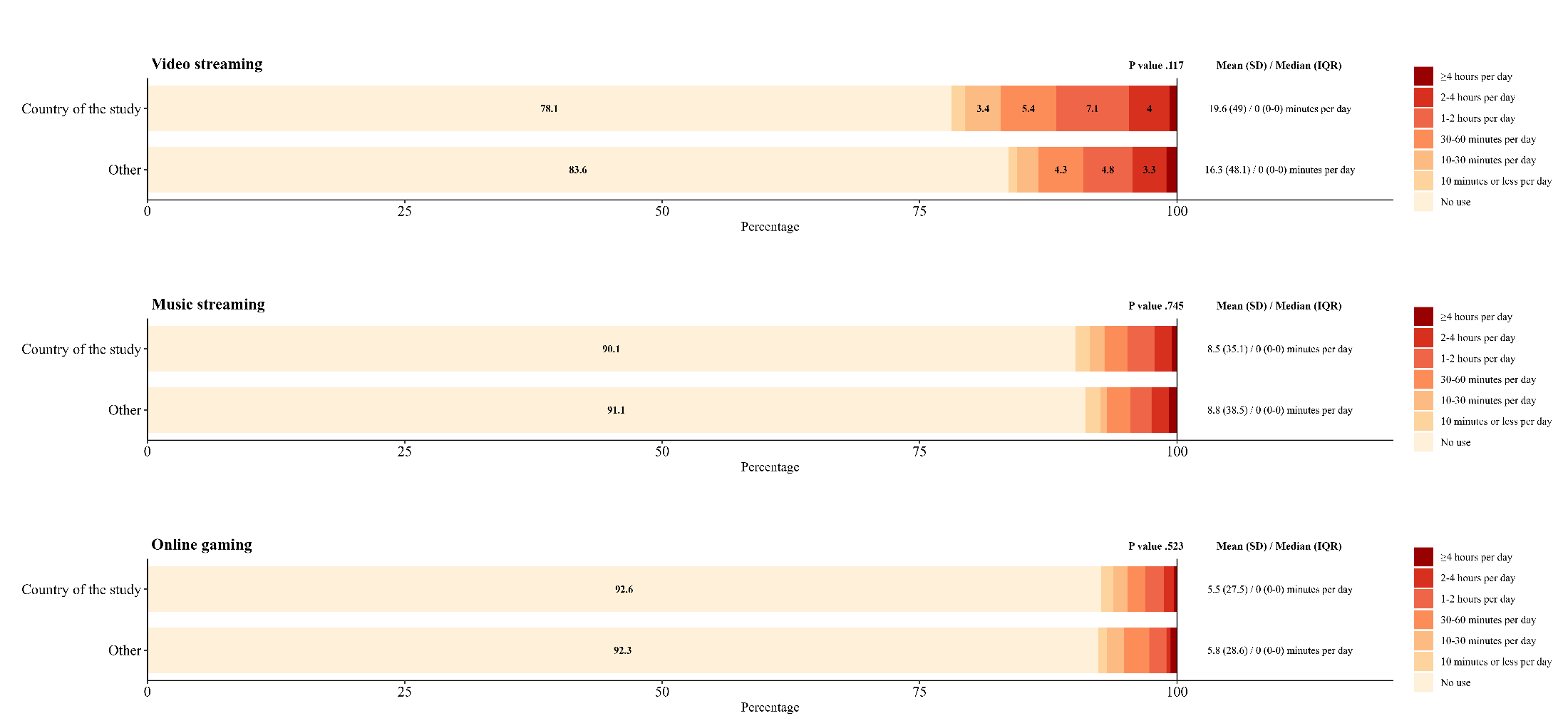
**

Figure S14. (Continuation).


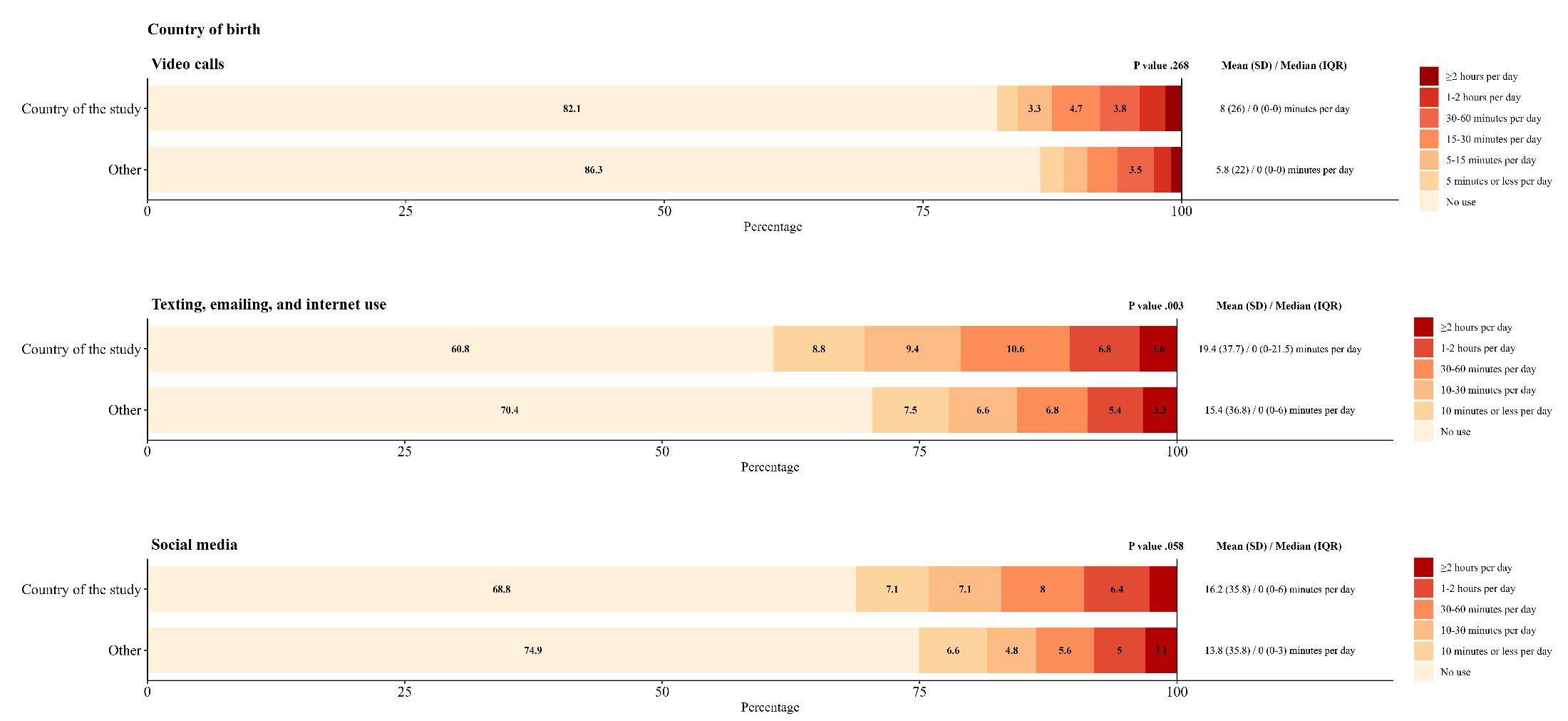


Figure S15. Frequencies of laptop activity duration per day by country of birth and activity in young adults.

**
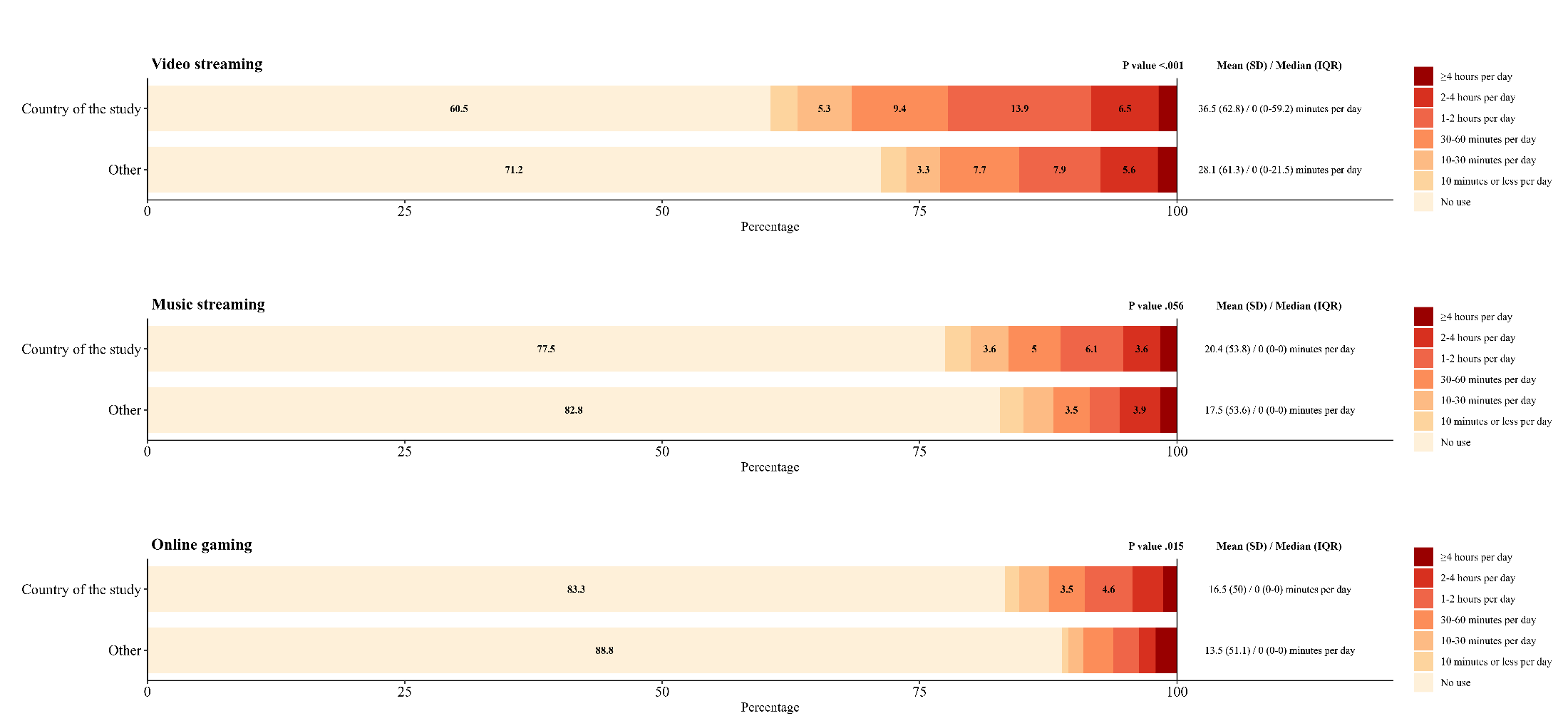
**

Figure S15. (Continuation).


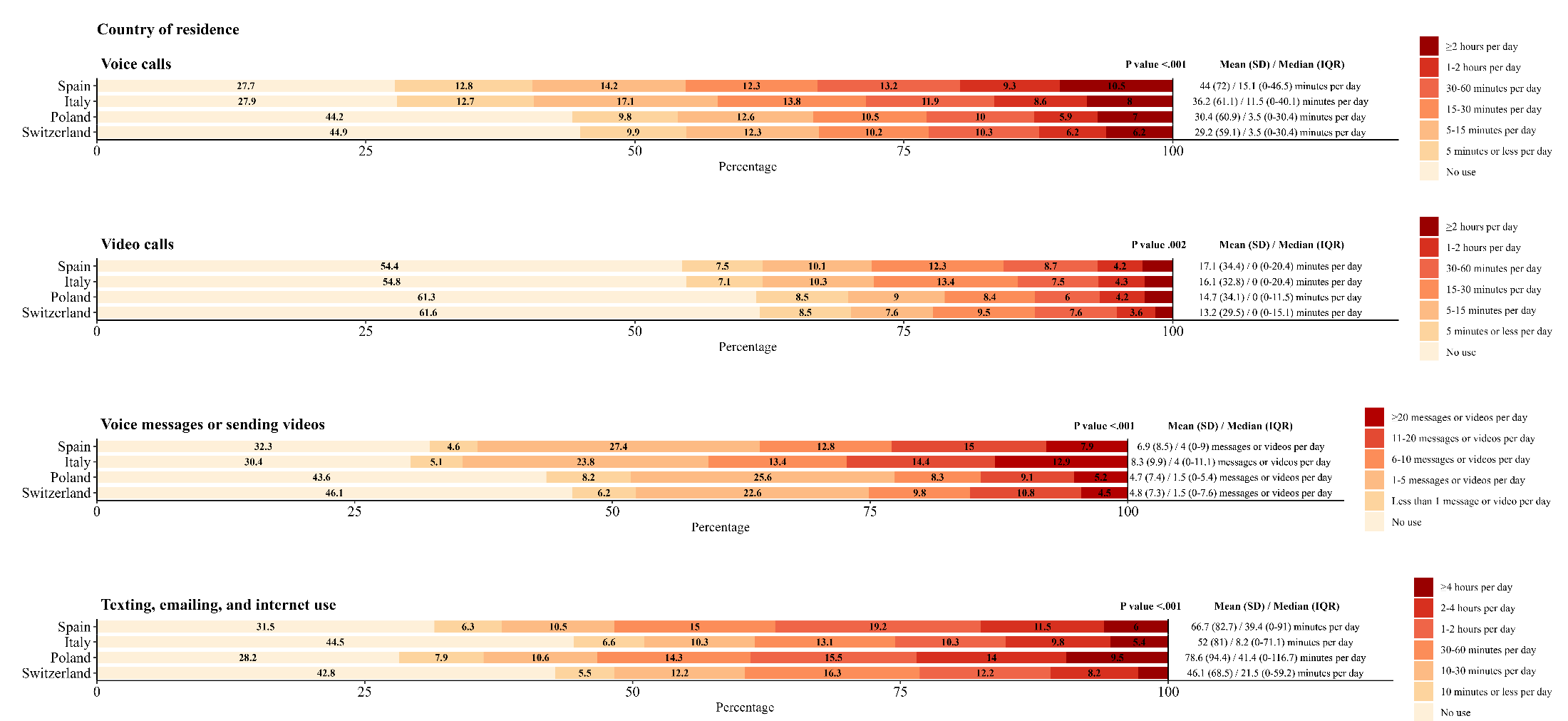


Figure S16. Frequencies of smartphone activity duration per day by country of residence and activity in young adults.

**
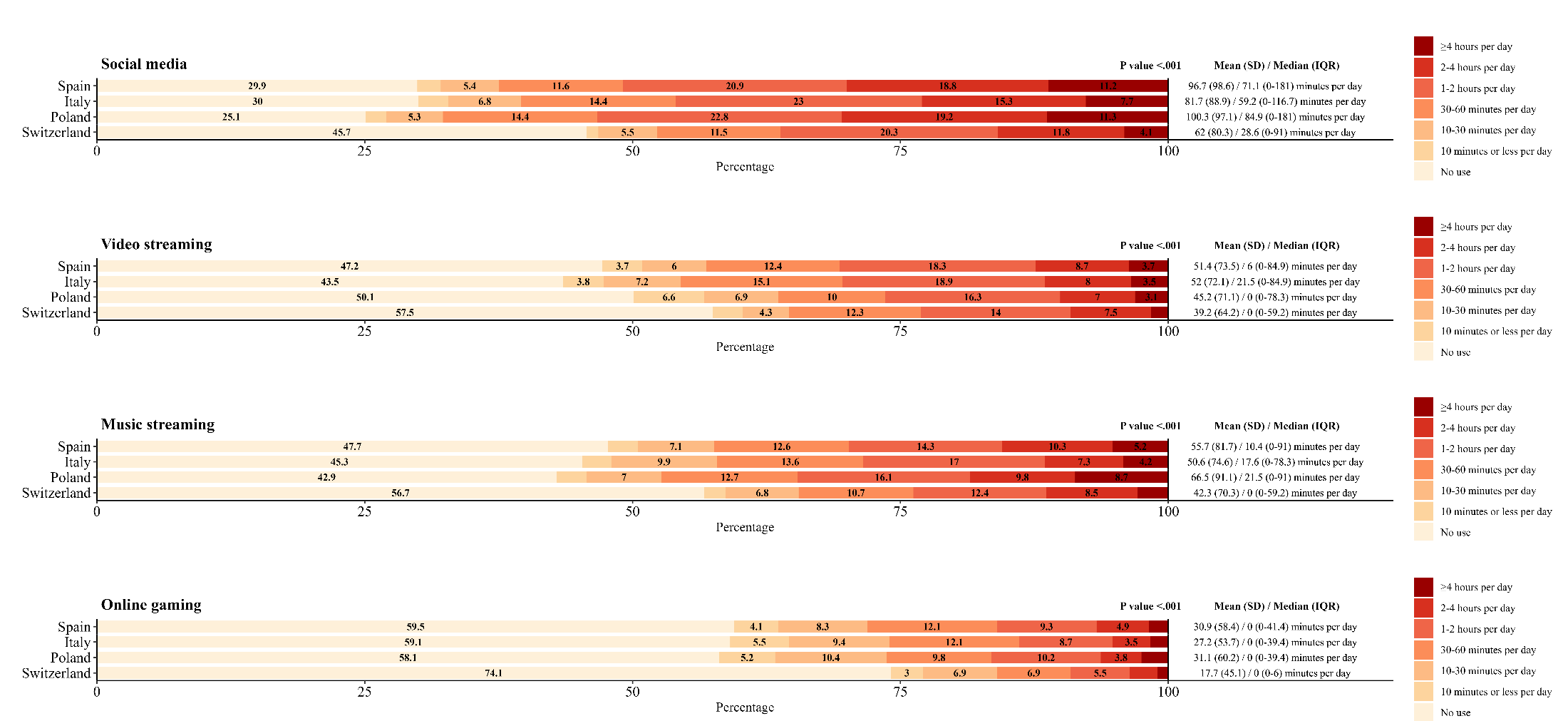
**

Figure S16. (Continuation).


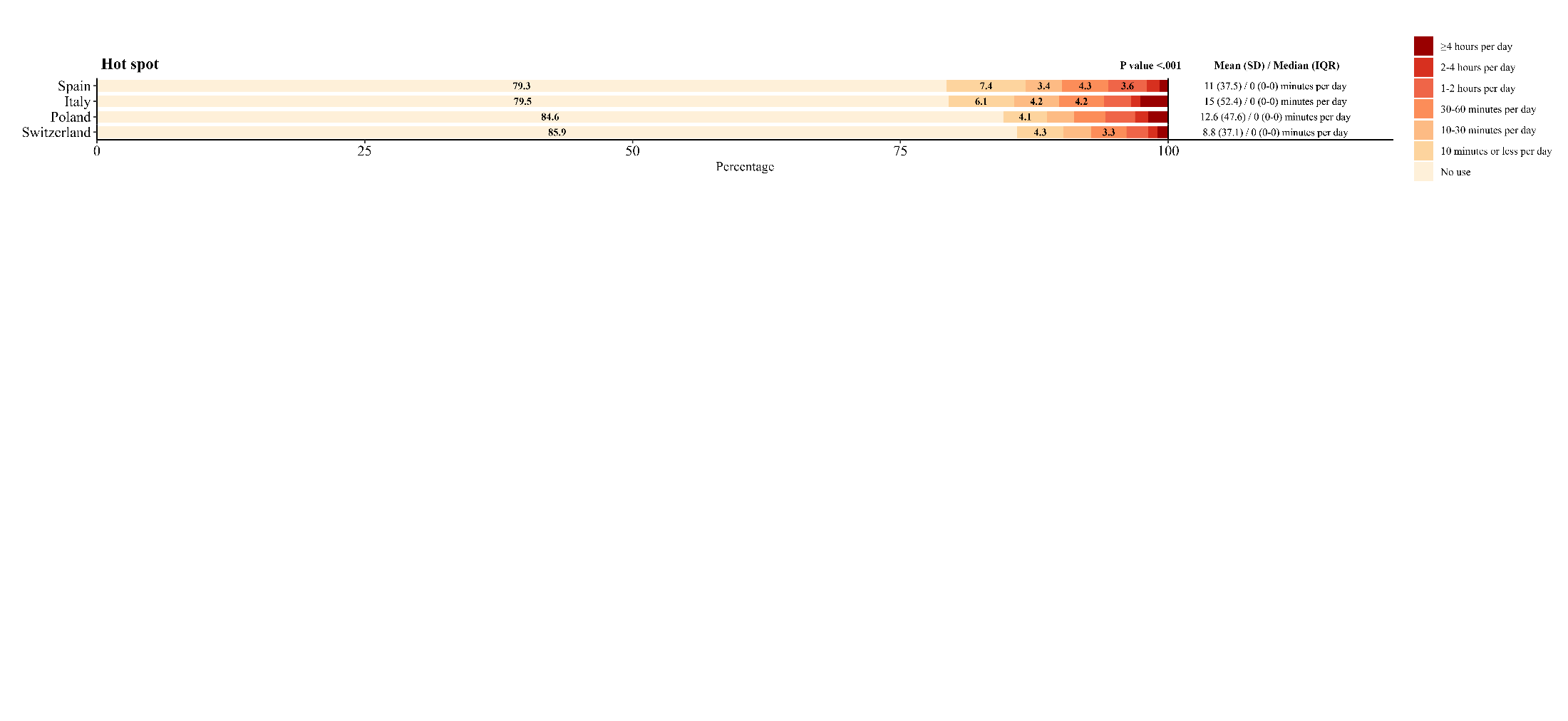


Figure S16. (Continuation).


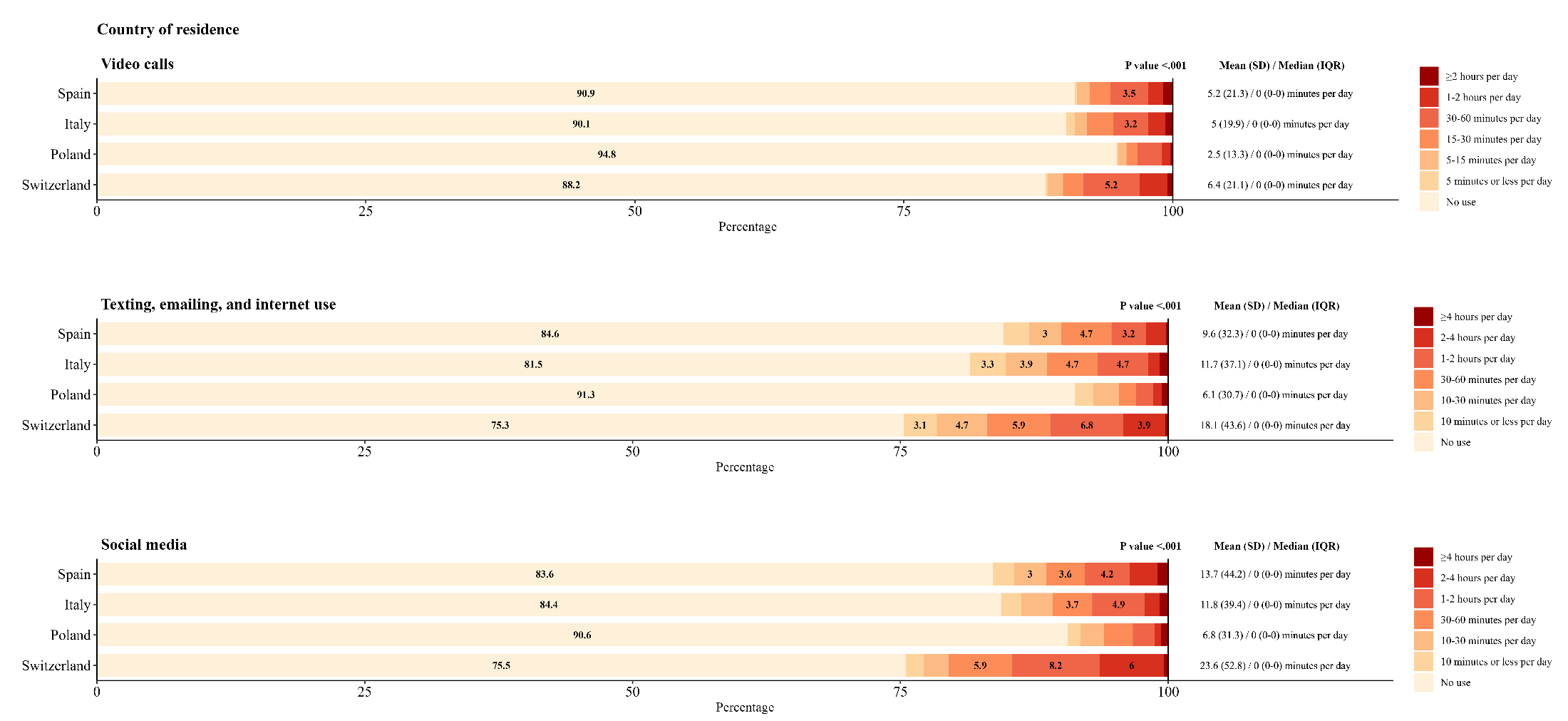


Figure S17. Frequencies of tablet activity duration per day by country of residence and activity in young adults.

**
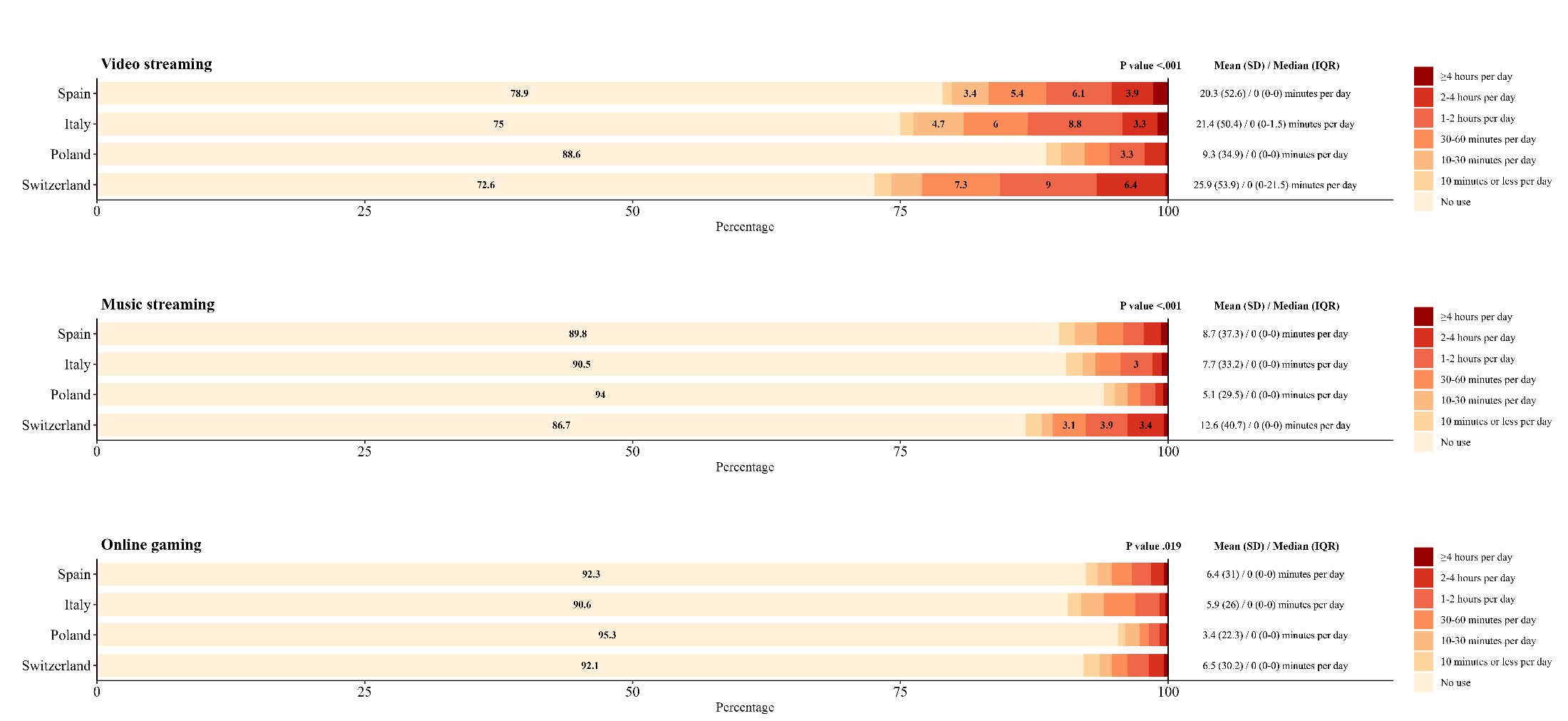
**

Figure S17. (Continuation).


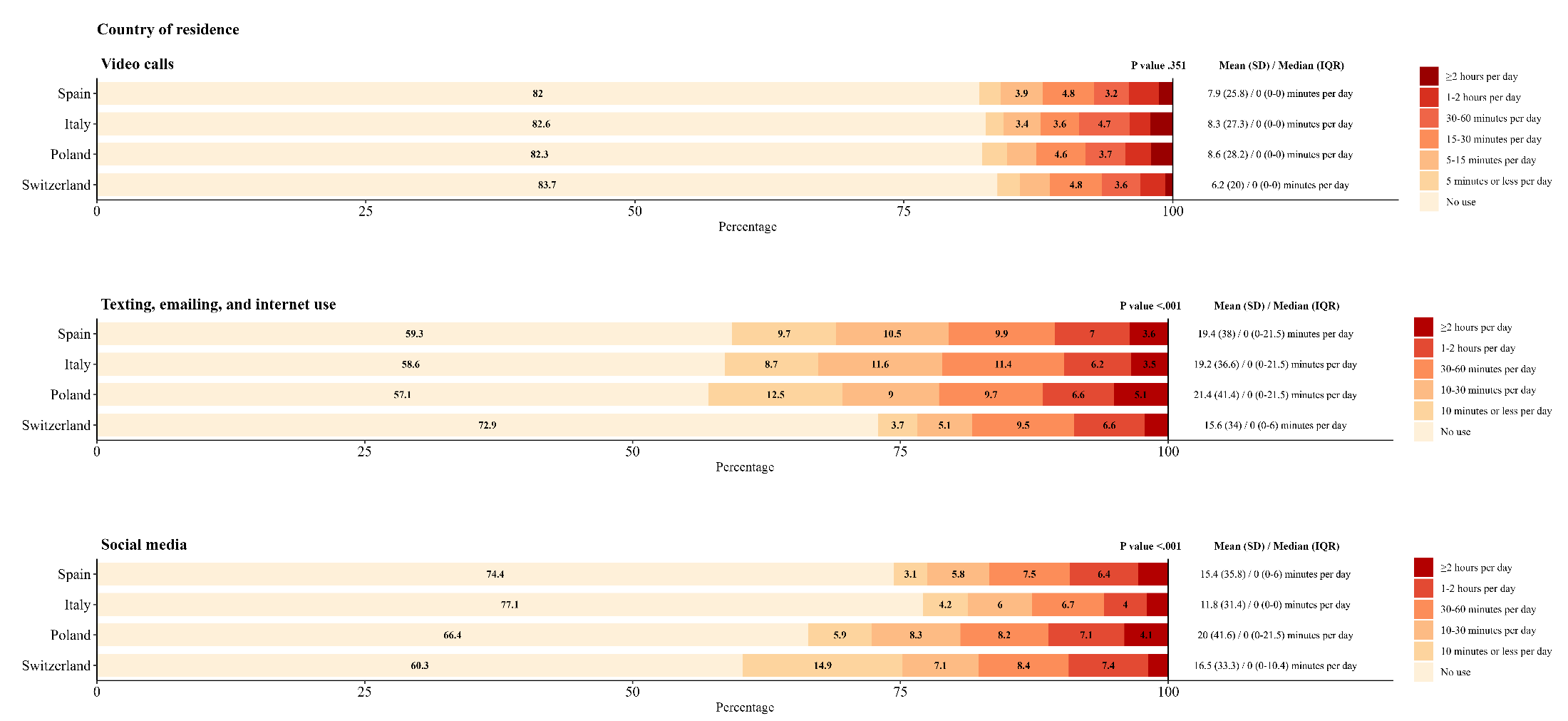


Figure S18. Frequencies of laptop activity duration per day by country of residence and activity in young adults.

**
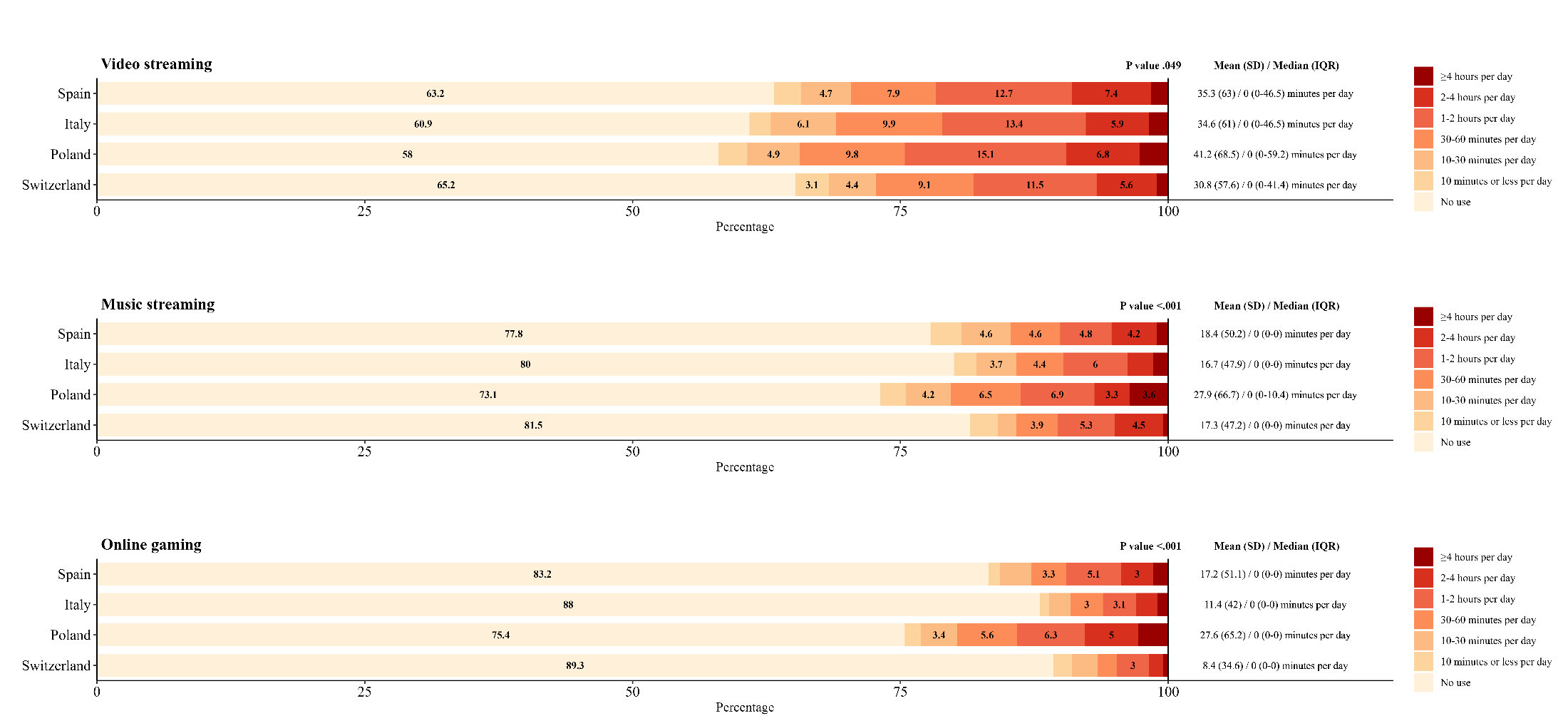
**

Figure S18. (Continuation).


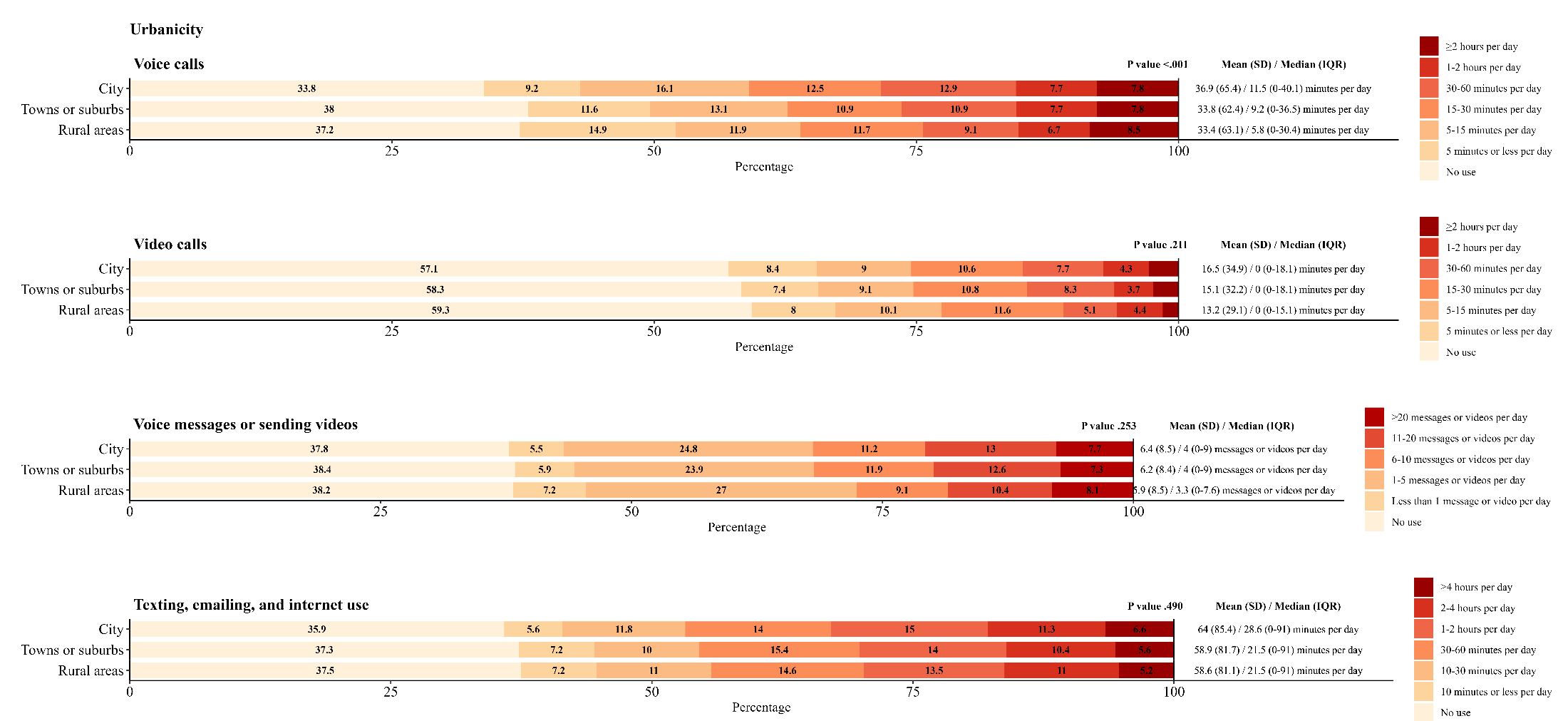


Figure S19. Frequencies of smartphone activity duration per day by urbanicity and activity in young adults.

**
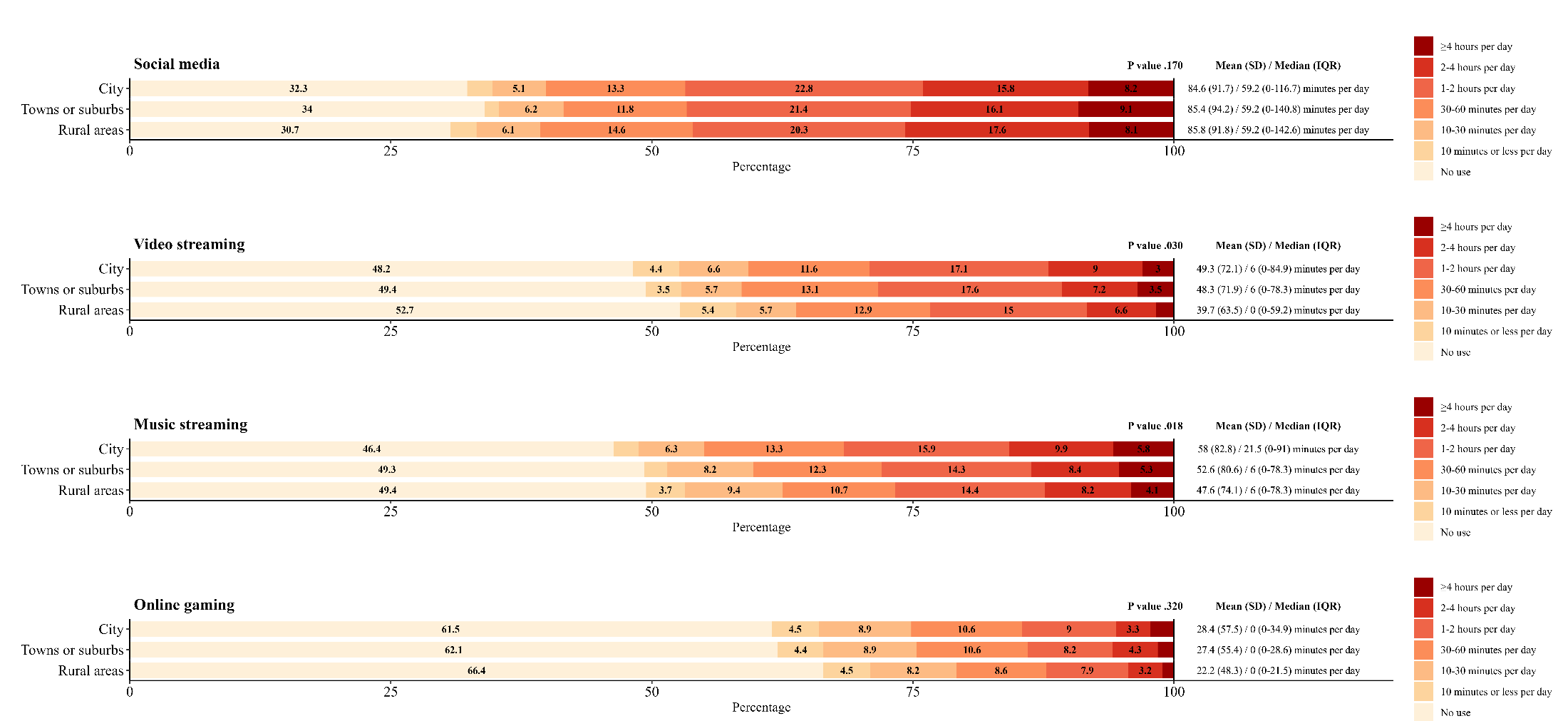
**

Figure S19. (Continuation).


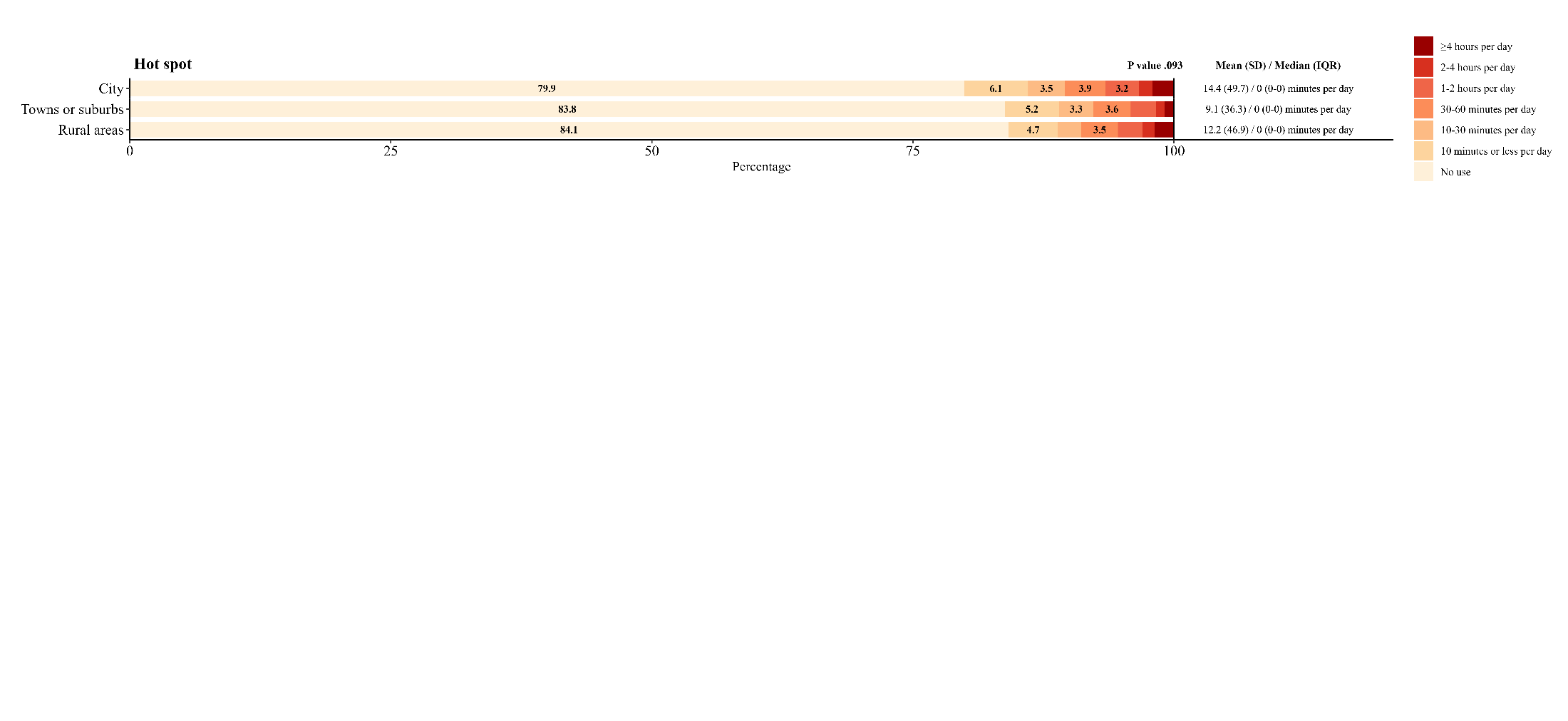


Figure S19. (Continuation).


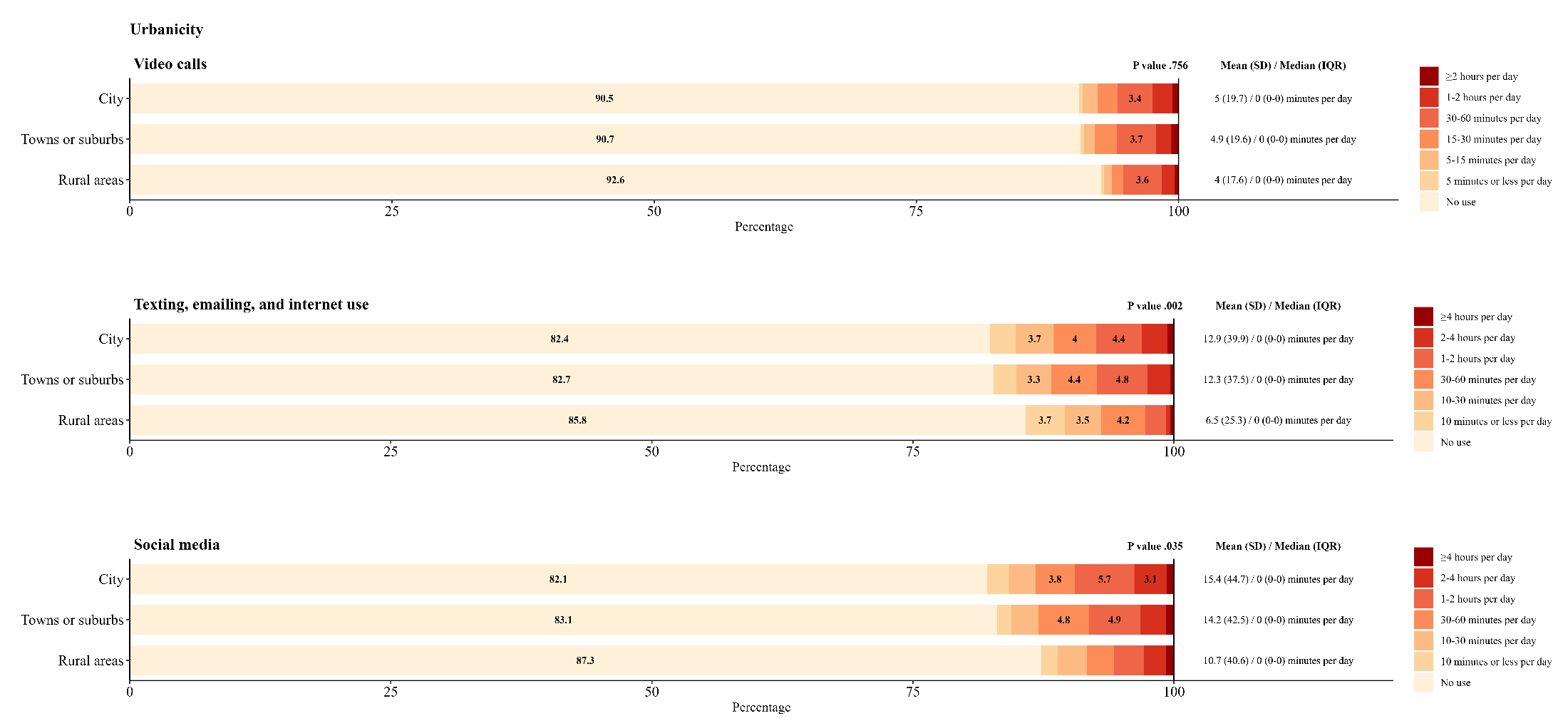


Figure S20. Frequencies of tablet activity duration per day by urbanicity and activity in young adults.

**
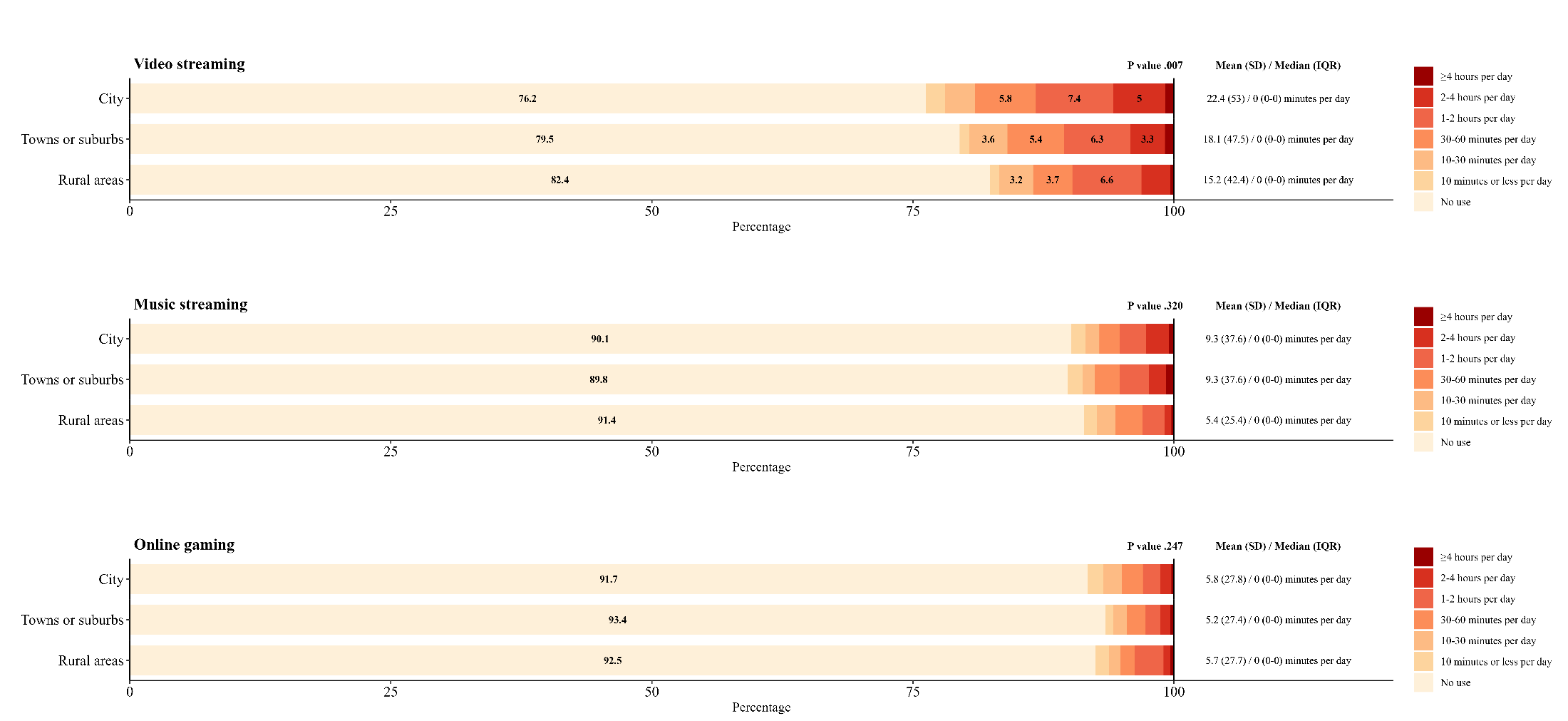
**

Figure S20. (Continuation).


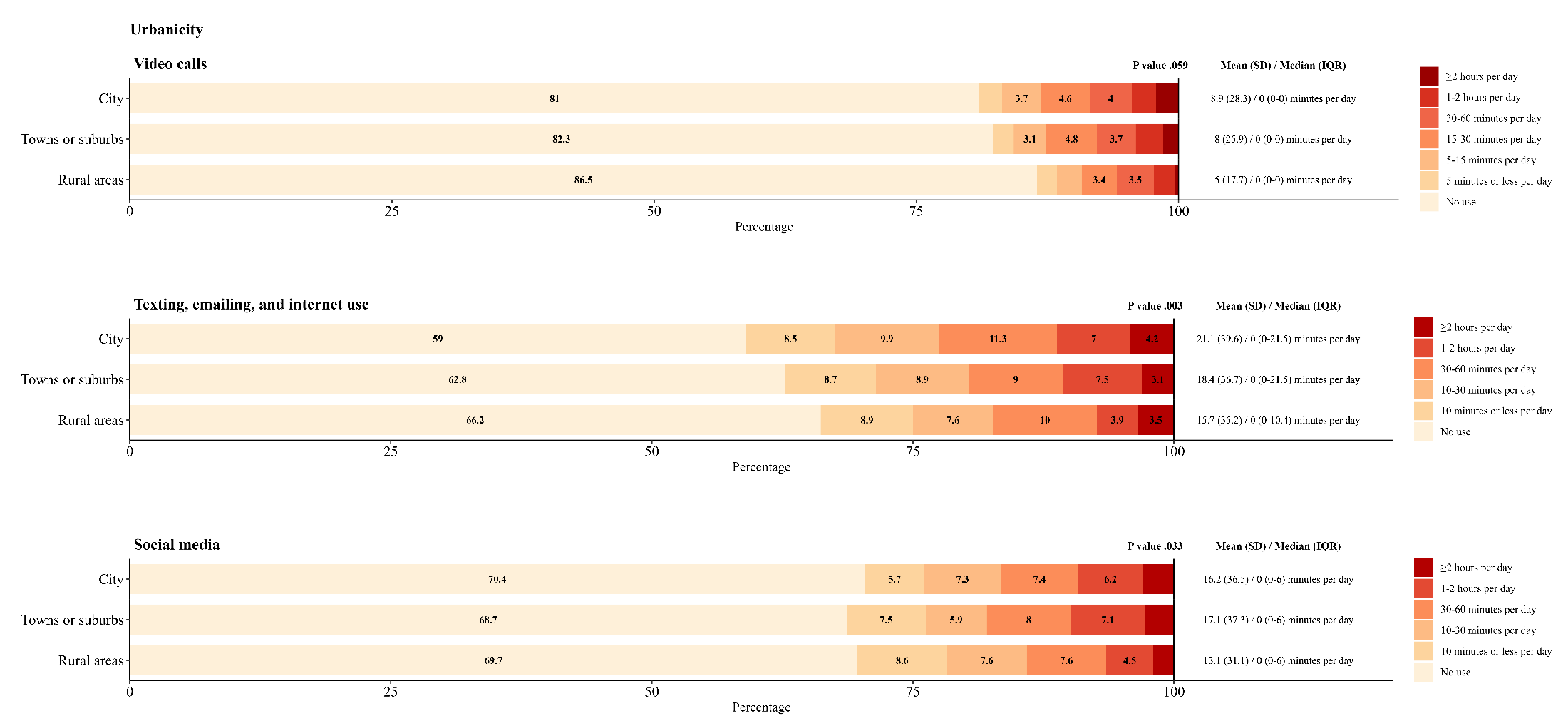


Figure S21. Frequencies of laptop activity duration per day by urbanicity and activity in young adults.

**
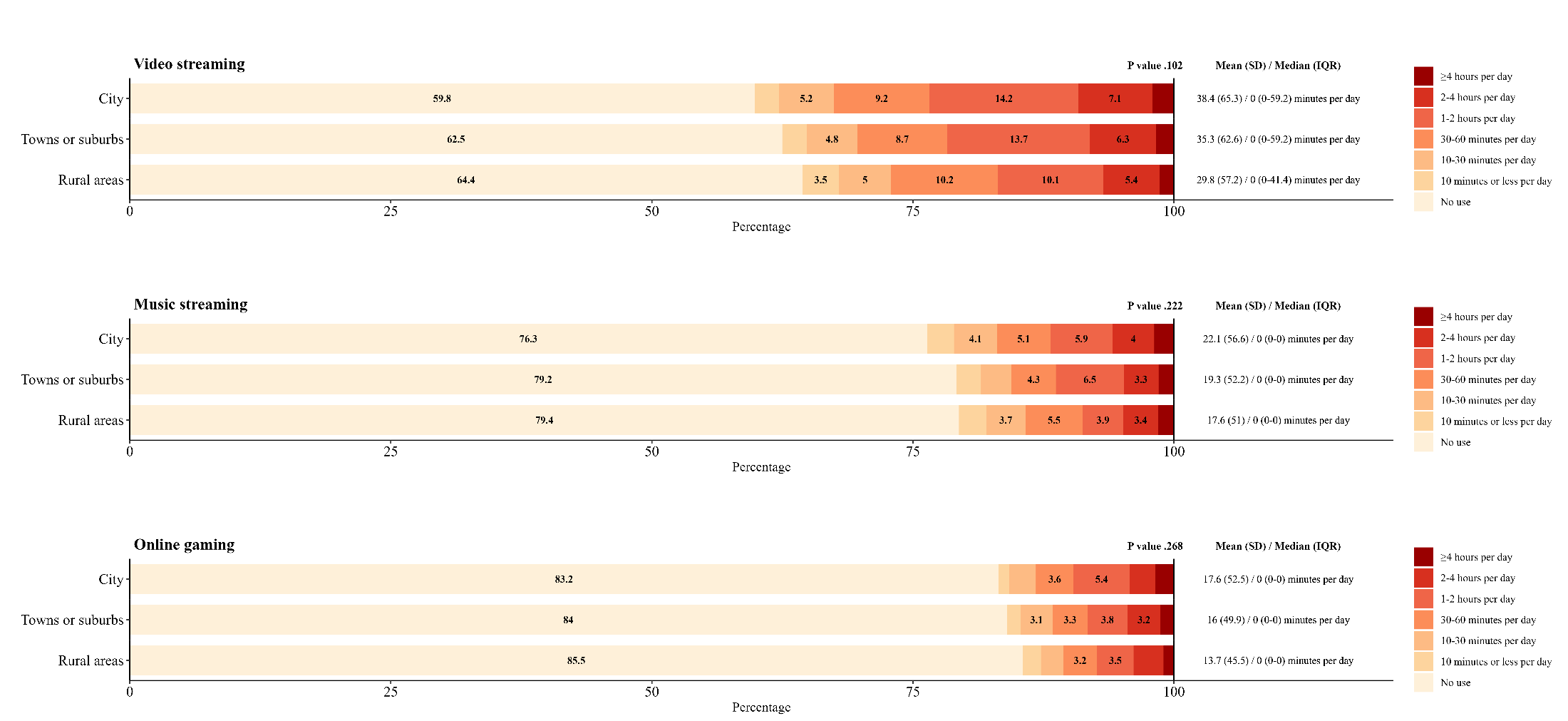
**

Figure S21. (Continuation).
