# Supplementary material for: Characterizing Digital Communication Device Use Among Young People From 4 European Countries: Cross-Sectional Survey Study
Source: J Med Internet Res. 2025 Dec 23;27:e76767. doi: 10.2196/76767 (PMC12724067; doi:10.2196/76767)
Supplement: Checklist 1 [file jmir-v27-e76767-s003.docx]

Checklist for Reporting Results of Internet E-Surveys (CHERRIES)

| ***Item Category*** | ***Checklist Item*** | ***Explanation*** |
| --- | --- | --- |
| **Design** |  |  |
|  | Describe survey design | Our target population consisted of a convenience sample of 4,000 young people (1,000 per country) aged 16-25 in Italy, Poland, Spain, and Switzerland in the year 2023. |
| **IRB (Institutional Review Board) approval and informed consent process** |  |  |
|  | IRB approval | This study was approved by the Research Ethics Committee of the Parc de Salut Mar (2023/10829). |
|  | Informed consent | Participants were informed that they were participating in the GOLIAT research project, and that that one of the aims of this project includes characterizing and monitoring patterns and levels of RF-EMF exposure. Furthermore, they were also informed that the objective of this survey was to investigate the use of mobile and digital communication devices in young people and to better any changes in these technologies. Participants were told that the survey would take roughly between 15 to 20 minutes, and that the researchers would only store their survey responses. This study was completely voluntary and they could withdraw their consent at any time for any reason without explanation. |
|  | Data protection | Responses to the survey were provided anonymized by CINT to ISGlobal. Personal data was never collected nor stored by ISGlobal. For personal data as a participant of surveys responsible by CINT, participants data was protected in accordance with the General Data Protection Regulation (EU) 2016/679 (GDPR) |
| **Development and pre-testing** |  |  |
|  | Development and testing | The usability and technical functionality of the survey was tested beforehand by the research team and on 223 participants. After an initial screening of the survey to resolve any unexpected issues that could have arisen, a full launch was performed. |
| **Recruitment process and description of the sample having access to the questionnaire** |  |  |
|  | Open survey versus closed survey | This was a closed survey. |
|  | Contact mode | Individuals were contacted on the internet through email. |
|  | Advertising the survey | The survey was announced through email to participants that are part of the CINT platform. The announcement contained information on what the study was about, our institution, the estimated duration to answer the questionnaire, and that compensation would be rewarded through CINT at the end of the questionnaire. |
| **Survey administration** |  |  |
|  | Web/E-mail | This survey was sent out as an email, where automatic methods captured the responses through a webpage. |
|  | Context | Not applicable as the survey was not advertised on web sites. |
|  | Mandatory/voluntary | This was a voluntary survey. |
|  | Incentives | Monetary incentives were offered for completion of the questionnaire. |
|  | Time/Date | The data was collected in the month of July 2023. |
|  | Randomization of items or questionnaires | No randomization was used. |
|  | Adaptive questioning | Questions were conditionally displayed based on the responses of whether the participant responded that they used the device on a regular basis (i.e., at least once per week in the last three months). Additionally, questions regarding certain activities on each device were also conditionally displayed on whether they answered they preformed the activity on a regular basis. |
|  | Number of Items | The number of questionnaire items per page was 1. |
|  | Number of screens (pages) | The number of pages depended on the conditional questions for each device and activity. This could range from a minimum of 15 screens to a maximum of 53 screens. |
|  | Completeness check | It was mandatory for the participant to answer all shown questionnaire items. If not, the participant was not allowed to continue to the next item in the questionnaire and was given an error of non-completeness. |
|  | Review step | Respondents were able to review and change answers through a back button, however no summary after completion was displayed to the respondent. |
| **Response rates** |  |  |
|  | Unique site visitor | A unique visitor was an individual that was already signed up to participate in CINT surveys that met the required target population we pre-specified, that is, between 16-25 years old. |
|  | View rate (Ratio of unique survey visitors/unique site visitors) | Not applicable. |
|  | Participation rate (Ratio of unique visitors who agreed to participate/unique first survey page visitors) | 85,598 participants were invited, with 6,180 participants initially agreeing to participate (participation rate of 7.22%) |
|  | Completion rate (Ratio of users who finished the survey/users who agreed to participate) | Of the 6,180 participants who agreed to participate, after screening out participants based on non-completion of the survey, unsubscribing from the platform, or quotas being filled, 4,000 participants were obtained. (Completeness rate of 64.72%) |
| **Preventing multiple entries from the same individual** |  |  |
|  | Cookies used | According to CINT, the survey platform uses a variety of cookies to set unique user IDs to avoid users accessing the survey multiple times. |
|  | IP check | According to CINT, the survey platform tracks the user’s IP address to avoid accessing the survey multiple times. |
|  | Log file analysis | Not applicable. |
|  | Registration | To participate in the study, users needed to first login in through CINT. Once they had completed the survey, users were not allowed to fill in the survey an additional time. |
| **Analysis** |  |  |
|  | Handling of incomplete questionnaires | Only complete questionnaires were analyzed, as incomplete questionnaires were screened out during collection. |
|  | Questionnaires submitted with an atypical timestamp | Due to the fact that the questionnaire was adaptive based on the devices used by individuals, questionnaires below two and a half minutes were excluded and another participant was invited, while participants who answered lower times were carefully examined to determine if the answers made sense in context to their responses (eg. Those who only used smartphones would have significantly reduced time of completion compared to those who used all devices) |
|  | Statistical correction | Non-interlocking quotas were used for: gender, age group, and place of residency of the participant. Gender was defined as male (50%), female (50%), other. Age group was defined as 16-18y (30%), 19-22y (40%), 23-25y (30%). Place of residency was defined as city (> 100,000 residents) (40%), towns or suburbs (10,000-100,000 residents) (40%), rural (< 10.000 residents) (20%). |
